# A Transcription Factor Map as Revealed by a Genome-Wide Gene Expression Analysis of Whole-Blood mRNA Transcriptome in Multiple Sclerosis

**DOI:** 10.1371/journal.pone.0014176

**Published:** 2010-12-01

**Authors:** Carlos Riveros, Drew Mellor, Kaushal S. Gandhi, Fiona C. McKay, Mathew B. Cox, Regina Berretta, S. Yahya Vaezpour, Mario Inostroza-Ponta, Simon A. Broadley, Robert N. Heard, Stephen Vucic, Graeme J. Stewart, David W. Williams, Rodney J. Scott, Jeanette Lechner-Scott, David R. Booth, Pablo Moscato, for the ANZgene Multiple Sclerosis Genetics Consortium

**Affiliations:** 1 Centre for Bioinformatics, Biomarker Discovery & Information-Based Medicine, University of Newcastle, and Hunter Medical Research Institute, Newcastle, Australia; 2 Australian Research Council Centre of Excellence in Bioinformatics, St Lucia, Australia; 3 Westmead Millennium Institute, University of Sydney, Westmead, Australia; 4 School of Medicine, Griffith University, Brisbane, Australia; 5 Department of Neurology, Gold Coast Hospital, Southport, Australia; 6 Hunter Medical Research Institute, Newcastle, Australia; 7 John Hunter Hospital, University of Newcastle, Newcastle, Australia; 8 Department of Computer Engineering, Amirkabir University of Technology, Tehran, Iran; 9 School of Computer Science and Software Engineering, The University of Western Australia, Crawley, Australia; 10 Departamento de Ingeniería Informática, Universidad de Santiago de Chile, Santiago, Chile; University of Manchester, United Kingdom

## Abstract

**Background:**

Several lines of evidence suggest that transcription factors are involved in the pathogenesis of Multiple Sclerosis (MS) but complete mapping of the whole network has been elusive. One of the reasons is that there are several clinical subtypes of MS and transcription factors that may be involved in one subtype may not be in others. We investigate the possibility that this network could be mapped using microarray technologies and contemporary bioinformatics methods on a dataset derived from whole blood in 99 untreated MS patients (36 Relapse Remitting MS, 43 Primary Progressive MS, and 20 Secondary Progressive MS) and 45 age-matched healthy controls.

**Methodology/Principal Findings:**

We have used two different analytical methodologies: a non-standard differential expression analysis and a differential co-expression analysis, which have converged on a significant number of regulatory motifs that are statistically overrepresented in genes that are either differentially expressed (or differentially co-expressed) in cases and controls (e.g., V$KROX_Q6, p-value <3.31E-6; V$CREBP1_Q2, p-value <9.93E-6, V$YY1_02, p-value <1.65E-5).

**Conclusions/Significance:**

Our analysis uncovered a network of transcription factors that potentially dysregulate several genes in MS or one or more of its disease subtypes. The most significant transcription factor motifs were for the Early Growth Response EGR/KROX family, ATF2, YY1 (Yin and Yang 1), E2F-1/DP-1 and E2F-4/DP-2 heterodimers, SOX5, and CREB and ATF families. These transcription factors are involved in early T-lymphocyte specification and commitment as well as in oligodendrocyte dedifferentiation and development, both pathways that have significant biological plausibility in MS causation.

## Introduction

Multiple Sclerosis (MS) [1] is an autoimmune disease characterised by chronic inflammatory demyelination of the central nervous system. The disorder affects millions of people worldwide and is the most common chronic disease of the central nervous system that begins in early to middle adult life [2] (its prevalence in the USA is 0.9 per 1,000 [3]). MS is a multifactorial and heterogeneous disease, and although its causes are currently unknown it is increasingly clear that both genetic and environmental factors contribute to susceptibility [2], [4]–[6]. Evidence for genetic risk includes familial aggregation [5], [7] and differences in prevalence between ethnic groups [8]–[10] (caucasians of Northern European ancestries appear to be particularly susceptible). It is now well established that certain genes influence susceptibility to MS – human leukocyte antigen HLA DRB1*1501 [11], IL2RA, IL7R, CLEC16A [12]–[16], and others [17]–[23].

There are three principal clinical subtypes of MS, the most prevalent being: relapsing remitting (RRMS), where unpredictable relapsing episodes alternate with periods of remission; primary progressive (PPMS), where there is steady progression of the illness from onset; and secondary progressive (SPMS), where after an initial relapsing remitting pattern, there is progressive neurological decline with or without relapses. The question of whether some MS subtypes are associated with particular susceptibility genes is open and merits further examination. Currently there is only modest evidence for any association [24], [25], and these have been challenged [26].

Previous research has demonstrated the potential of differential gene expression profiling in elucidating complex traits, including disease susceptibility, when expression is measured in tissue related to the trait under study [27]. A companion study [28] to the work presented here applied traditional statistical analysis to whole blood mRNA transcriptome of all known genes in a gene expression study of MS. The results were combined with results from the genome-wide association study published by the ANZgene GWAS[21]. The expression of 48,000 gene probe sequences in human HT-12 beadarray chips (Illumina Inc., CA, USA) hybridized with cDNA from 99 untreated MS patients (36 RRMS, 43 PPMS, and 20 SPMS) and 45 age-matched healthy controls, identifying over 19,000 gene probes expressed in the blood cells with a large number that were differentially expressed between the controls and MS sample groups. Functional profiling of the differentially expressed genes revealed that the cells in whole blood are more active in MS, being engaged in translation and energy production through oxidative phosphorylation to a greater extent. Additionally, T cells accounted for the greatest percentage of differentially expressed (in controls versus MS collectively) immune cell “tagging” genes. Together, these results are consistent with the prevailing view that autoreactive T cells play a key role in the pathogenesis of MS.

Evidence suggests that transcription factors are involved in the pathogenesis of MS and other autoimmune diseases [29], [30]. For example, it has previously been observed that members of the NF-kappaB, STAT, AP-1 and E2F families [30]–[56], IRF-1 [36], [46], [47], [57], IRF-2 [58], IRF-5 [59], IRF-8 [20], CREB [36], [46], PPARgamma and PPARalpha [60]–[68], SP1 [59], SP3 [69]–[71], RORC [72], NR4A2, TCF2 [73], ETS-1 [74] and FOXP3 [75]–[85] may be implicated in MS and its disease subtypes. This paper aims to complement the companion study by uncovering, thanks to combinatorial optimization and differential co-expression methods and a different focus in the analysis, transcription factors that potentially dysregulate many genes in MS. The working hypothesis is that, given consistent sets of genes exhibiting differential expression or co-expression patterns between two classes, this change is attributed to a set of transcription factors. Ultimately, our goal is to piece together relationships and infer a *network* of transcription factors that are implicated in MS and its subtypes as inferred from the differential expression and co-expression of several hundreds of genes.

We are aware that a cautionary note is required to our study and all others that aim to correlate variations in the transcriptome of whole-blood with gene expression and its regulation mechanisms and their consequences in the brain (for instance, those of oligodendrocyte de-differentiation). There exist few studies in the area. In a recent study [86], the consistency between expression patterns between brain and blood was analysed. Although important differences were observed (around 90% of all transcripts examined presented variations in the alternative splicing index in brain and blood), a large number of brain transcripts (4,100) co-expressed in blood samples. This means, on the biomarker identification phase, that they can be further explored in experimental studies of either blood or cell lines from patients with MS.

As the effects of processing whole blood may also contribute to changes of these gene expression patterns, our cautionary note remains until we could better narrow the valid consistencies. In some sense, the diagnostic need of a simple blood test for MS, as well as our present atlas of expression changes with this technology will certainly motivate useful studies like the one in [86] to bridge this gap.

The data discussed in this publication have been deposited in NCBI's Gene Expression Omnibus [87], and are accessible through GEO Series accession number GSE17048 (http://www.ncbi.nlm.nih.gov/geo/query/acc.cgi?acc=GSE17408).

## Results

Two different analyses were used to define potentially relevant transcription factors. Gene expression using microarray detection techniques utilize short sequences (“probes”) that target specific genes, and even specific protein isoforms (particularly true in Illumina technology). All differential expression or co-expression analysis here are performed on individual probe identifiers, while transcription factor overrepresentation are performed based on the target genes identifiers. The first identified gene probes differentially expressed between the four different clinical groups in the study's sample set; the second, groups of gene probes pair-wise differentially *co-expressed* between control and MS samples. The first analysis is better at revealing differences in gene expression between the sample groups, while the second aims to find specific groups of genes which have lost their normal pattern of co-expression and are affected by MS irrespective of disease subtype. Overrepresented binding motifs, and their corresponding transcription factors, were obtained for the groups of genes identified under both approaches.

Seven molecular genetic signatures were derived from the whole blood mRNA expression data collected in [28] (our companion study) and then profiled for overrepresented TRANSFAC motifs. Each signature contained a set of gene probe sequences that are differentially expressed between a pair of sample groups specified by the signature's denotation (C/MS, C/PP, C/RR, C/SP, PP/RR, RR/SP, or PP/SP); for example, C/PP contained genes differentially expressed between control and PPMS samples. The signatures were obtained using a three-step filtering process that discards the probes that are least relevant for distinguishing between the pair of sample groups according to formal criteria: Standard detection p-value initial filtering, then an entropy-based selection procedure [88], and finally, an *(α,β)-k-feature set* selection method [89], [90], which removes probes based on statistical significance, information content, and predictive ability respectively. More details are provided in the Materials and Methods section. This methodology has been used to identify robust gene sets associated to a variety of diseases, including Alzheimer's [89], [91], Parkinson's [92], and prostate cancer [93]. Gene sets obtained by this methodology maximise intra-class coherency and inter-class discriminatory power with the minimum number of genes[94].

**Figure 1:**
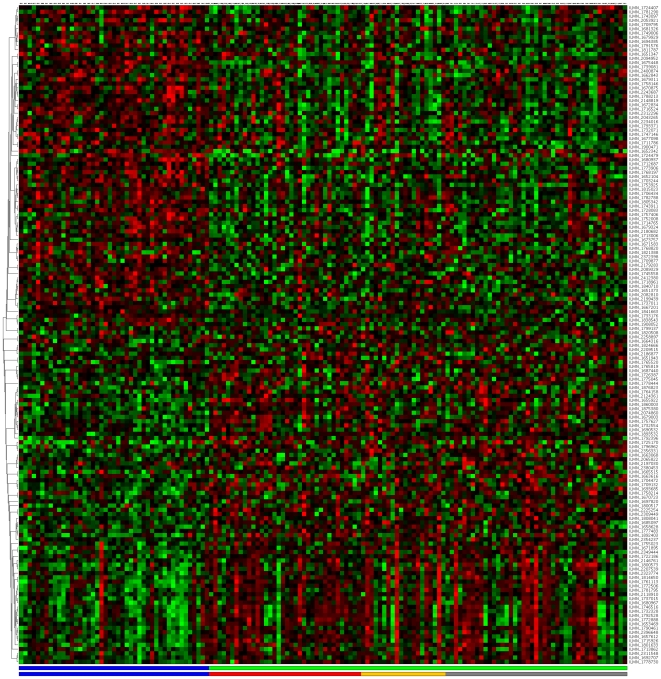
mRNA expression heatmap for the C/MS signature. Samples are placed along the horizontal axis and mRNA probe sequences along the vertical axis. Samples are colour-coded in the bottom horizontal bar (controls: blue; MS: green, as RR: red, SP: yellow, PP: grey). Clustering with a high-performance memetic algorithm (described in [285]) has ordered the samples and probe sequences, revealing four quadrants that are primarily up- (red) or down- (green) regulated. Note that division into up/down regulated is more coherent in the control samples than the MS samples.

**Figure 2 pone-0014176-g002:**
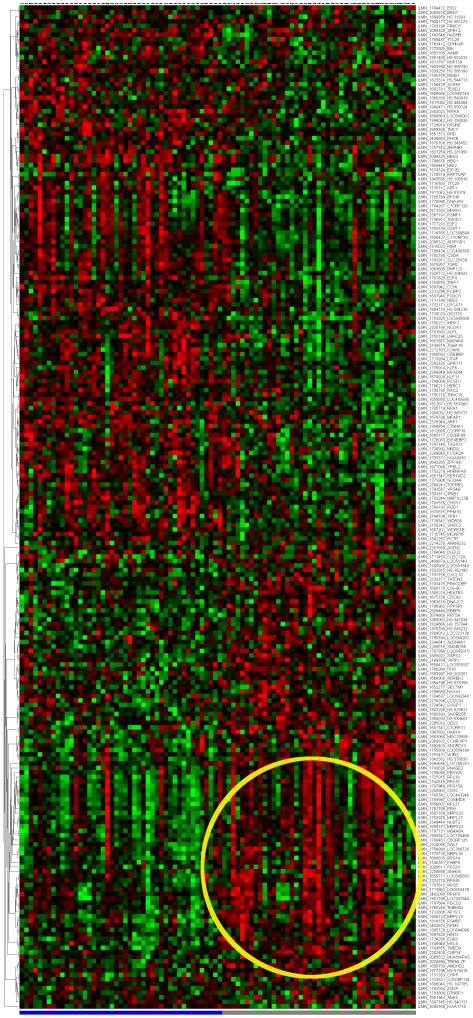
mRNA expression heatmap for the C/PP signature. Control samples (blue), PPMS (grey). Note the pattern of ‘bars’ near the bottom right of the heatmap (circled) where expression broadly alternates between up- (red) and down- (green) regulated in disease. This regular pattern could indicate that PPMS contains distinct subgroups with respect to gene expression.

**Figure 3 pone-0014176-g003:**
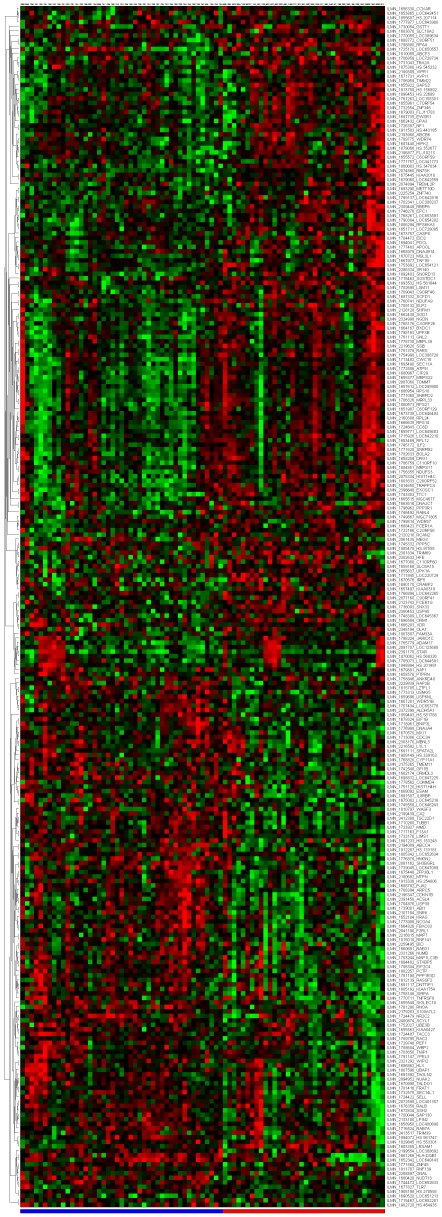
mRNA expression heatmap for the C/RR signature. Control samples (blue), RRMS (red).

**Figure 4 pone-0014176-g004:**
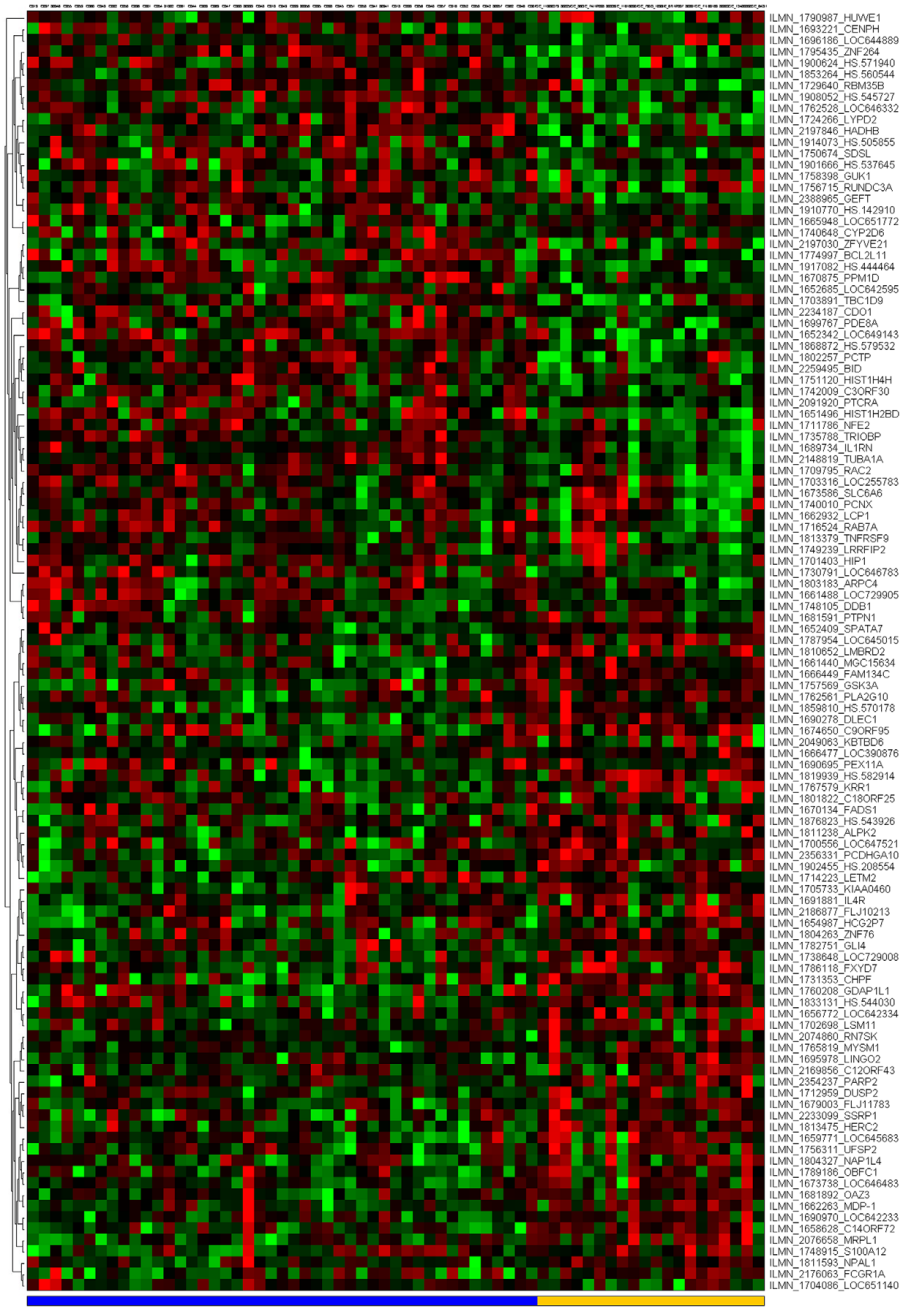
mRNA expression heatmap for the C/SP signature. Control samples (blue), SPMS (yellow).

**Figure 5 pone-0014176-g005:**
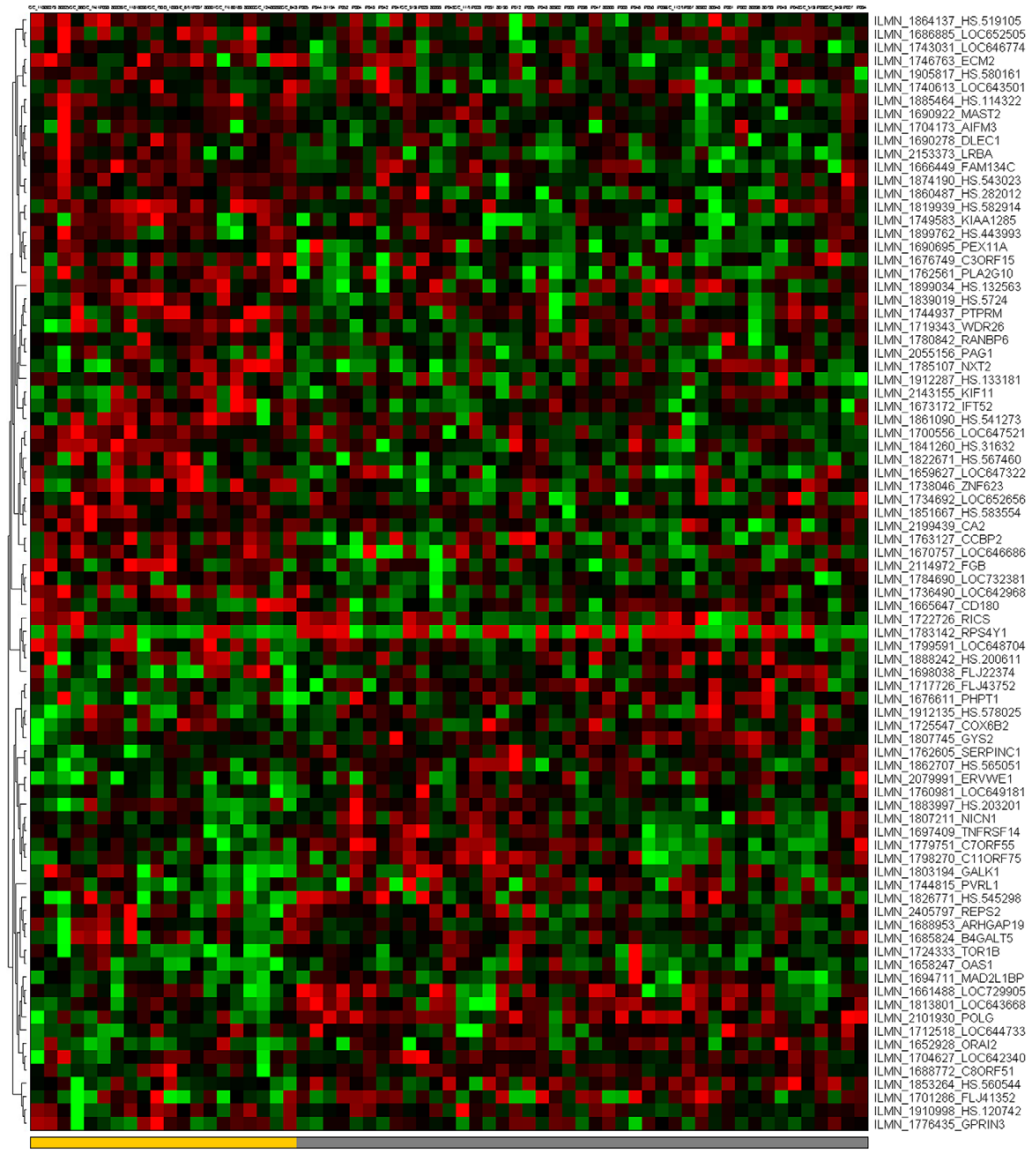
mRNA expression heatmap for the PP/SP signature. SPMS samples (yellow), PPMS (grey).

**Figure 6 pone-0014176-g006:**
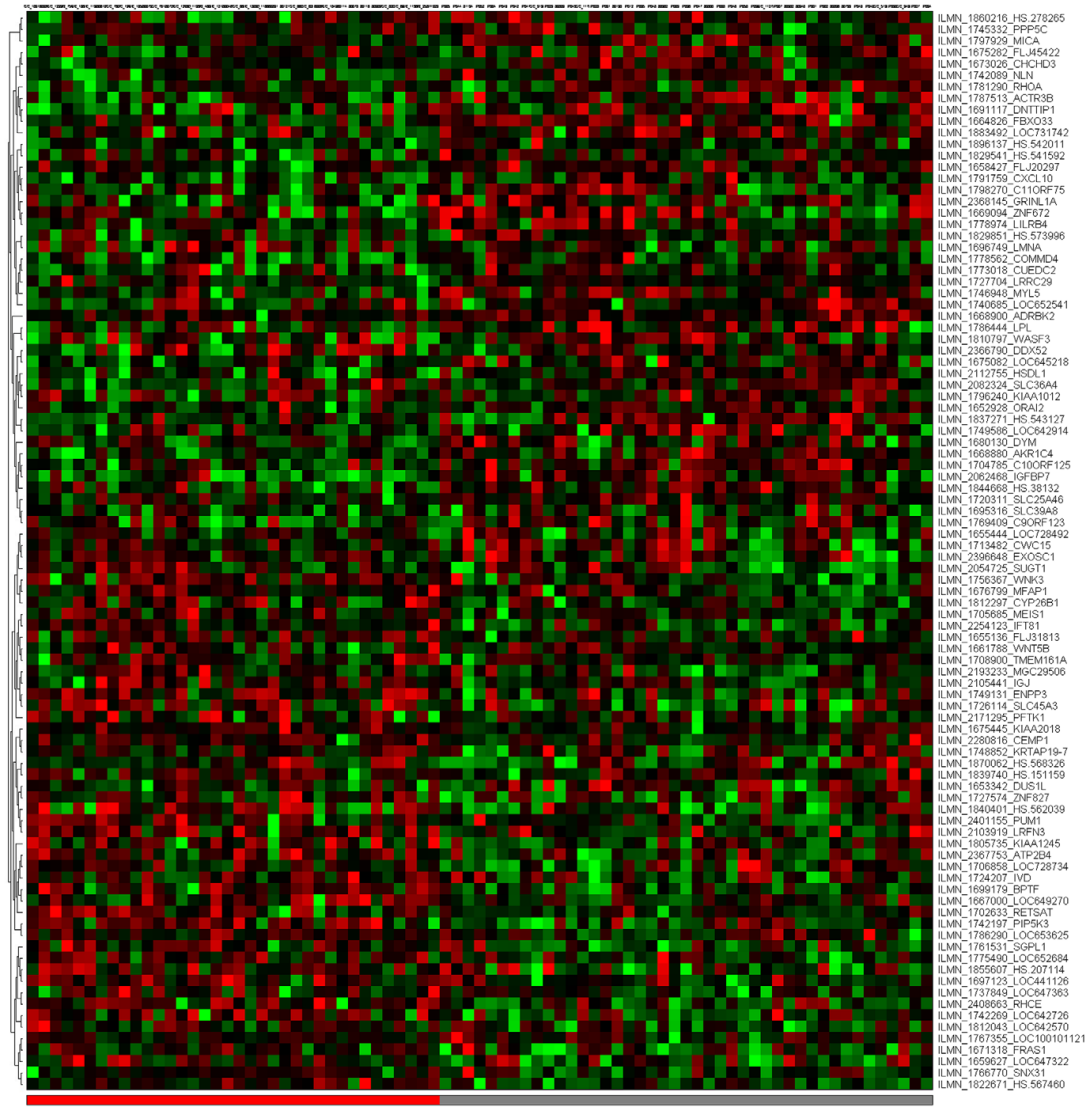
mRNA expression heatmap for the PP/RR signature. RRMS samples (red), PPMS (grey).

**Figure 7:**
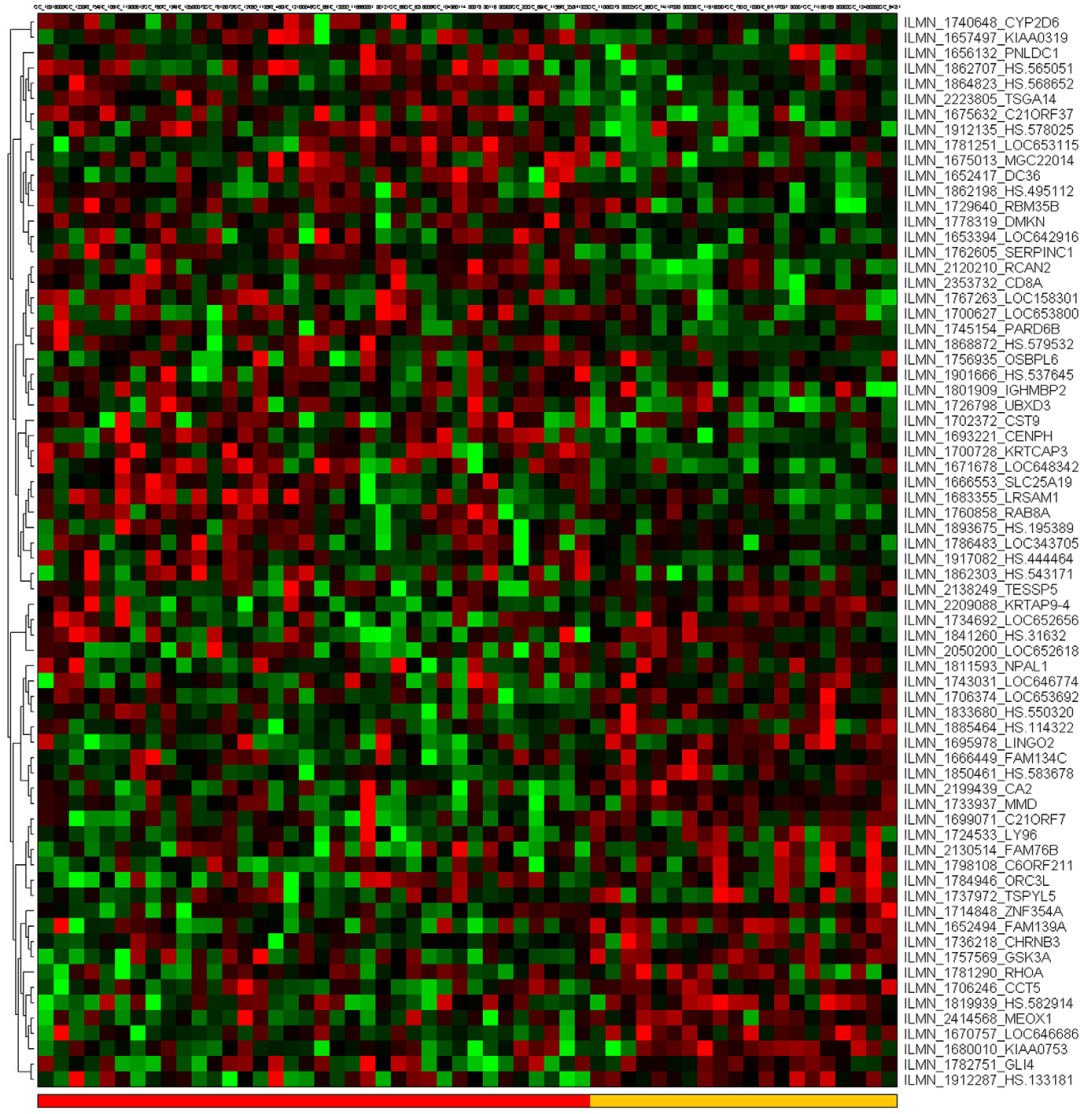
mRNA expression heatmap for the RR/SP signature. RRMS samples (red), SPMS (yellow).

Figures 1–7 depict the signatures and their corresponding clustered heatmaps, while detailed listings are provided in supporting information Table S1, Table S2 and Table S3, with combined summary, listing and overlap with the companion study signatures in supporting information Table S4. We note that the heatmap for C/MS (Figure 1) exhibits a lack of coherence in the MS samples, indicating that gene expression is heterogeneous over the MS group as a whole. Coherence increases when the MS subtypes are considered separately (C/PP: Figure 2, C/RR: Figure 3, C/SP: Figure 4, PP/SP: Figure 5, PP/RR: Figure 6, and RR/SP: Figure 7), suggesting a correlation between disease subtype and gene expression. The heatmaps containing SPMS samples (Figures 4, 5, and 7) are less coherent overall than those for PPMS (Figures 2, 5, and 6) and RRMS (Figures 3, 6, and 7), suggesting that SPMS is the most heterogeneous MS subtype with respect to gene expression in our study. A noticeable pattern of “bars” does appear in the PPMS samples in C/PP (Figure 2), though, which could be evidence of distinct PPMS subtypes with contrasting gene expression for the genes in the bars.

### Analysis of Differential Gene Expression

**Figure 8:**
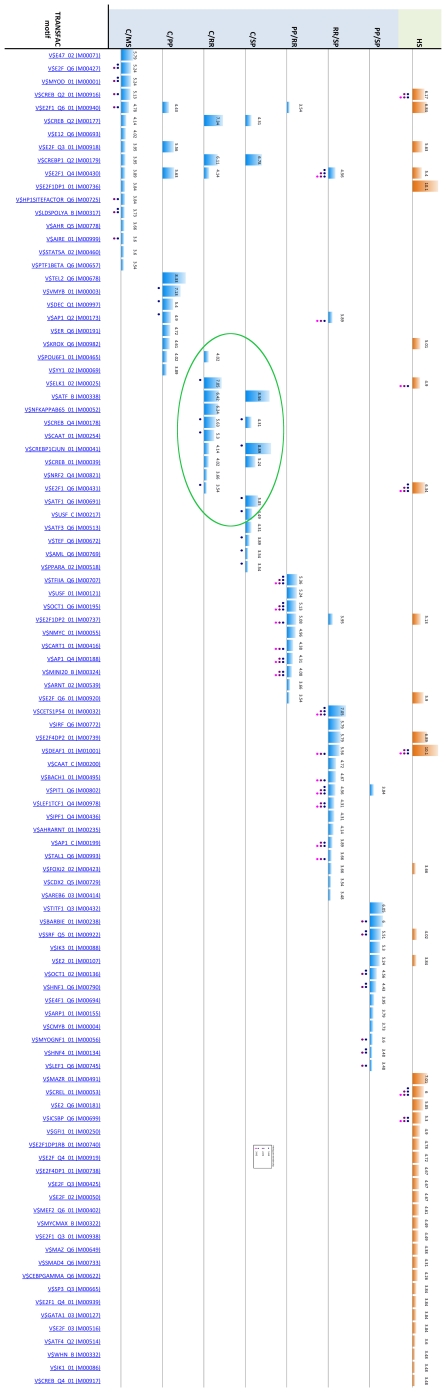
TRANSFAC motif profiles for the seven signatures (blue bars). Only significant motifs (*p*-value ≤0.05) are shown. Bar size is proportional to the negative natural logarithm of *p*-value, which is also given as a number. Note that, in contrast to other pairs of profiles, there is a sizeable intersection between the C/RR and C/SP profiles (green ring). “Key” regulators of each profile, which are found by hitting set analysis, are indicated by dots underneath a bar (see figure legend). The TRANSFAC profile for the set containing all the key regulators' coding genes is shown (orange bars); this profile in effect contains “higher level” regulators – i.e. a hierarchy with a next level of putative regulators of regulators.

A profile of overrepresented regulatory sequences was obtained for each of the seven signatures using the profiling tool GATHER [95] (See supporting information Table S5). The profiles contain TRANSFAC binding motifs occurring in the regulatory regions of the genes for each signature, which appear overrepresented with *p*-value <0.05. The complete profiles for all signatures are given in Figure 8. Inspection of the profiles reveals that, with the exception of C/RR and C/SP, there is only a modest intersection between any pair of profiles. In contrast, nearly half of the motifs in each of the C/RR and C/SP TRANSFAC profiles (six motifs) occur in the intersection of the pair. The C/RR and C/SP signatures share only 12 gene probes (the signatures contain 267 and 113 probes respectively), which is not enough to account for the amount of agreement between their TRANSFAC profiles. In fact, the composition of the TRANSFAC profiles was unchanged when the 12 probes were removed from C/RR and C/SP and the profiles recomputed. These observations suggest that any pathways of transcriptional dysregulation occurring in MS are likely to be largely distinct in the PPMS subtype, while overlapping considerably in RRMS and SPMS. (We note a similar finding in the companion study [28] using a different methodology: RRMS and SPMS share more dysregulated gene ontology pathways –principally immune regulation pathways– with each other than with PPMS.)

Additionally, we profiled the above TRANSFAC profiles themselves to reveal higher-level overrepresented regulatory sequences. These higher-level regulatory sequences – which effectively identify ‘regulators of regulators’ – were obtained by TRANSFAC profiling the coding genes of transcription factors corresponding to key sequences in the above TRANSFAC profiles. We considered the identification of the key sequences to be a problem of combinatorial optimization (specifically, a generalization of the Hitting Set problem) as described under Materials and Methods. The resulting higher-level sequences are given in Figure 8 (HS column) and Table 1.

**Table 1:** Regulators of regulators: binding motifs/transcription factors appearing in the higher level regulation network.

	Binding motif	Transcription factor	HS of	In which HS	*p-*value
1.	V$E2F1DP1_01 (M00736)	E2F-1/DP-1 heterodimer	HS-Clust	3-hs	3.31E-06
2.	V$E2F4DP2_01 (M00739)	E2F-4/DP-2 heterodimer	HS-Clust	1-, 3-hs	3.31E-06
3.	V$DEAF1_01 (M01001)	DEAF1	HS-Sig	2-, 3-hs	4.28E-05
4.	V$CREB_Q2_01 (M00916)	CREB	HS-Clust	1-, 2-, 3-hs	6.57E-05
5.	V$E2F1_Q6_01 (M00940)	E2F-1	HS-Clust	2-hs	7.87E-05
6.	V$KROX_Q6 (M00982)	KROX/EGR family	HS-Clust	1-, 3-hs	2.03E-04
7.	V$CREB_Q4_01 (M00917)	CREB family	HS-Clust	3-hs	7.25E-04
8.	V$HNF3B_01 (M00131)	HNF3beta	HS-Clust	2-, 3-hs	7.62E-04
9.	V$E2F1_Q6 (M00431)	E2F-1	HS-Sig	1-, 2-, 3-hs	1.76E-03
10.	V$CREB_Q2_01 (M00916)	CREB	HS-Sig	2-, 3-hs	2.09E-03
11.	V$CREL_01 (M00053)	c-Rel	HS-Sig	1-, 2-, 3-hs	2.48E-03
12.	V$ZTA_Q2 (M00711)	Zta	HS-Clust	1-, 2-, 3-hs	3.21E-03
13.	V$ICSBP_Q6 (M00699)	ICSBP	HS-Sig	2-, 3-hs	4.99E-03
14.	V$ELK1_02 (M00025)	ELK1	HS-Sig	3-hs	7.45E-03
15.	V$MAZ_Q6 (M00649)	MAZ	HS-Clust	2-, 3-hs	8.92E-03
16.	V$E2F_Q6 (M00427)	E2F	HS-Clust	3-hs	1.41E-02

**Figure 9:**
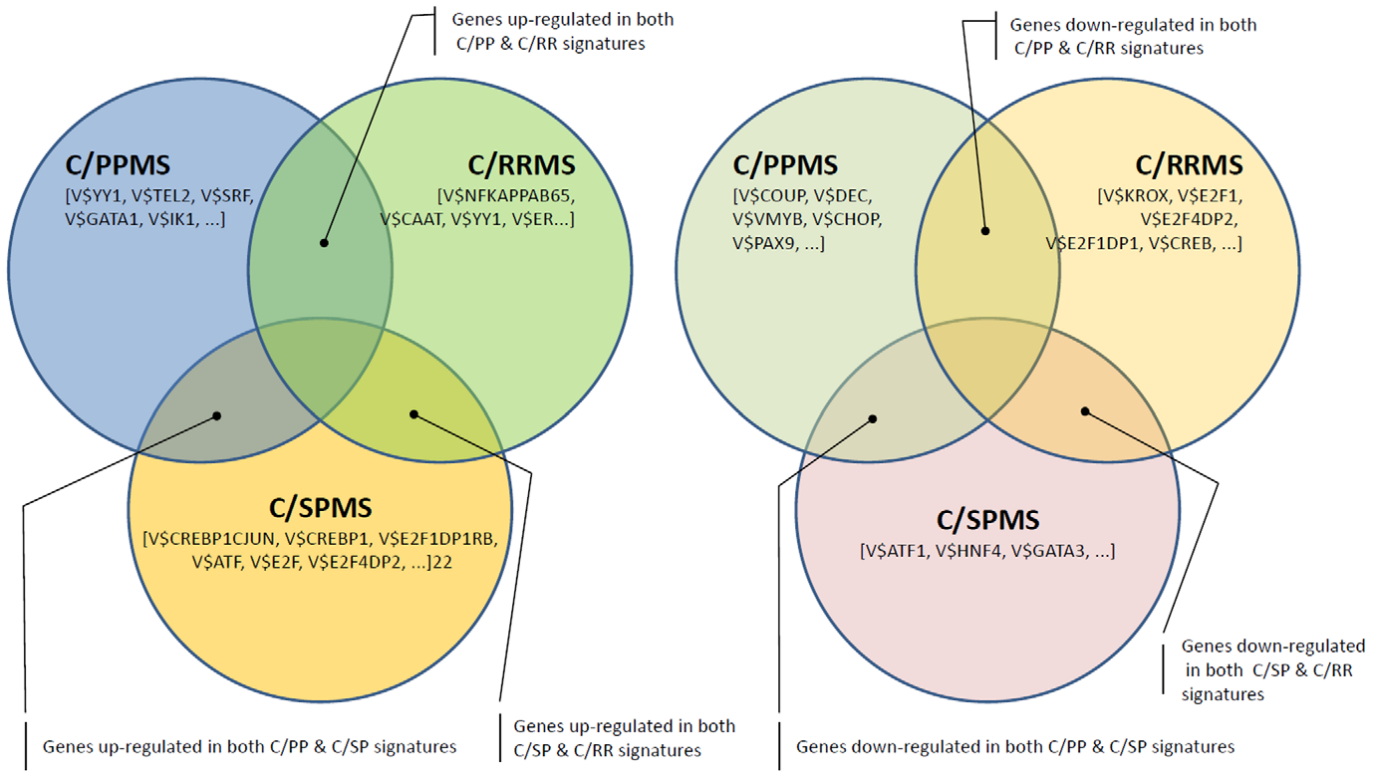
The A) up- and B) down-regulated signature subsets. (Note that when two groups were combined, the genes in the intersection are also included in the analysis). The most significant transcription factor motifs associated with a group are shown in square brackets.

**HS of** identifies the origin of the binding motif: ‘HS-Clust’ indicates the hitting set of hitting sets of star cluster profiles, ‘HS-Sig’ indicates the hitting set of hitting sets of signature profiles (see Figure 11 and Figure 9). **In which HS** identifies in which of the (1-, 2- or 3-) hitting sets does the binding motif appear.

#### Binding Motifs for KROX/EGR Family, ATF2, and YY1 are Significantly Overrepresented in MS Subtypes

We highlight the appearance of V$E2F1DP1_01 complex (p-value <4.28E-5), V$DEAF1_01 (p-value <4.28E-5), V$MAZR_01 (p-value <1.0E-3) and V$E2F4DP2_01 (p-value <1.1E-3). V$DEAF1_01 is a binding motif for the DEAF1 (deformed epidermal autoregulatory factor 1 homolog/NUDR/Supressin) transcription factor, which has been related to negative regulation of T-lymphocytes [96], and recently linked to altered tissue-specific antigen expression in the lymphoid system and autoimmune diabetes [97], [98].

**Figure 10:**
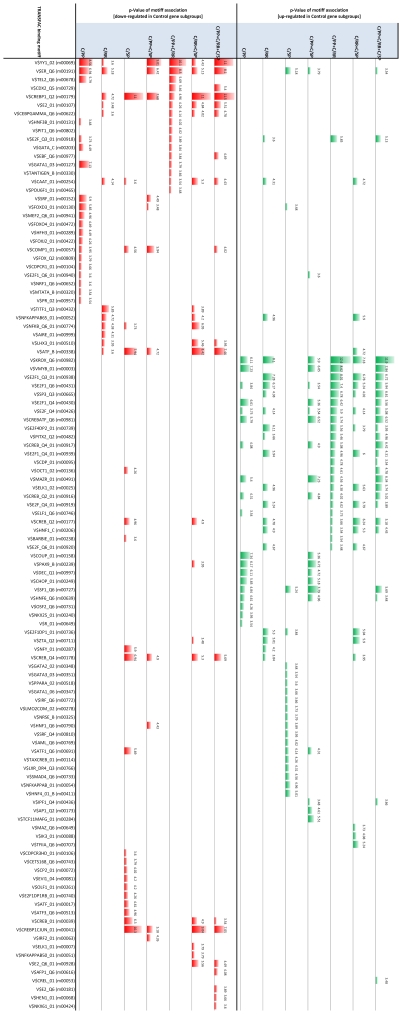
TRANSFAC motif profiles for the up- (red bars) and down- (green bars) regulated signature subsets. Profiles for subset unions are also shown; for instance C/PP+C/RR contains the up (red) or down (green) regulated genes from the union of the C/PP and C/RR signatures (Figure 8 depicts all the signature subsets and subset unions.) The bottom row (blue bars) is the profile for all genes in the C/PP, C/RR and C/SP signatures. Size of bar is proportional to the negative natural logarithm of *p*-value, which is also given as a number. Only significant motifs (*p*-value ≤0.05) are shown.

Finally, we profiled up-/down regulated subsets of the signatures to reveal any regulatory sequences associated with increased/decreased expression only. The intersection of the signatures is depicted in Figure 9, while Figure 10 shows the complete profiles for each signature subset. We note the transcription factor motifs for the KROX/EGR family (V$KROX_Q6, p-Value <3.31E-6), for ATF2 (V$CREBP1_Q2, p-Value <1.0E-5) and for YY1 (V$YY1_02, p-Value <1.65E-5) as the most significantly overrepresented (see Table 2, second block).

**Table 2:** The most significantly overrepresented TRANSFAC motifs found in this study.

	Binding motif	Transcription Factor	Profile	*p*-value
	*Via profiling the signatures*			
1.	V$CREBP1CJUN_01 (M00041)	ATF2/c-Jun heterodimer	C/SP	1.38E-4
2.	V$ATF_B (M00338)	ATF family	C/SP	1.92E-4
3.	V$TEL2_Q6 (M00678)	TEL2	C/PP	2.41E-4
4.	V$CREB_Q2 (M00177)	CREB	C/RR	6.49E-4
5.	V$VMYB_01 (M00003)	V-MYB	C/PP	8.01E-4
6.	V$ELK1_02 (M00025)	ELK1	C/RR	8.67E-4
7.	V$CETS1P54_01 (M00032)	ETS1	RR/SP	8.67E-4
8.	V$CREBP1_Q2 (M00179)	ATF2	C/SP	1.14E-3
9.	V$ATF_B (M00338)	ATF family	C/RR	1.63E-3
10.	V$NFKAPPAB65_01 (M00052)	NF-KappaBeta (p65)	C/RR	1.95E-3
	*Via profiling the up/down regulated signature subsets*			
1.	V$KROX_Q6 (M00982)	KROX/EGR family	C/PP+C/RR (-)	3.31E-6
2.	V$KROX_Q6 (M00982)	KROX/EGR family	C/PP+C/RR+C/SP (-)	6.59E-6
3.	V$CREBP1_Q2 (M00179)	ATF2	C/PP+C/RR+C/SP (+)	9.93E-6
4.	V$CREBP1_Q2 (M00179)	ATF2	C/RR+C/SP (+)	1.65E-5
5.	V$CREBP1_Q2 (M00179)	ATF2	C/SP (+)	1.65E-5
6.	V$YY1_02 (M00069)	YY1	C/PP+C/RR+C/SP (+)	1.65E-5
7.	V$CREBP1CJUN_01 (M00041)	ATF2/c-Jun heterodimer	C/SP (+)	3.30E-5
8.	V$YY1_02 (M00069)	YY1	C/PP+C/RR (+)	4.28E-5
9.	V$CREBP1CJUN_01 (M00041)	ATF2/c-Jun heterodimer	C/RR+C/SP (+)	1.19E-4
10.	V$YY1_02 (M00069)	YY1	C/PP+C/SP (+)	1.22E-4
	*Via profiling the star groups*			
1.	V$SOX5_01 (M00042)	SOX5	final_1	6.59E-6
2.	V$CREB_Q4_01 (M00917)	CREB family	final_2	6.59E-6
3.	V$CREBATF_Q6 (M00981)	CREB and ATF families	final_1	9.93E-6
4.	V$HTF_01 (M00538)		final_12	1.65E-5
5.	V$E2F_Q4 (M00426)	E2F/DP heterodimers	final_2	2.65E-5
6.	V$E2F1_Q6_01 (M00940)	E2F-1	final_2	2.65E-5
7.	V$NRF2_01 (M00108)	NFE2L2 (NRF2)	final_10	2.65E-5
8.	V$CREB_Q2_01 (M00916)	CREB family	final_1	3.30E-5
9.	V$MINI20_B (M00324)		final_1	3.30E-5
10.	V$CREB_Q4_01 (M00917)	CREB family	final_1	6.57E-5

In the Profile column, (+) and (−) indicate up- and down-regulated subsets respectively. The ‘+’ in names like C/PP+C/RR (−) indicates the union of the named signatures.

#### Remarks on the presence of some probes in the genetic signature that discriminates Controls from MS patients

The heterogeneity of the C/MS signature makes it relatively difficult to talk about individual probes, as it is unlikely that they will have the same pattern of expression across all subtypes of MS. Nevertheless, we highlight the following for future discussion. First, a probe for LOC649143 (similar to HLA class II histocompatibility antigen, DRB1-9 beta chain precursor (MHC class I antigen DRB1*9) (DR-9) (DR9)) has a tendency to be consistently upregulated in controls but not in MS samples. Second, a probe for LOC650557 (similar to HLA class II histocompatibility antigen, DQ(W1.1) beta chain precursor (DQB1*0501)) has the opposite behaviour, a tendency to be downregulated in controls. CASP8 (caspase 8, apoptosis-related cysteine peptidase) appears upregulated in MS and RRMS groups; the protective effect of its inhibition to oligodendrocytes has been suggested in [99]. Finally, several MS samples have higher expression for a probe of KIR2DS5 (Homo sapiens killer cell immunoglobulin-like receptor) [100], [101] than in controls (KIR2DS5 was also part of a study of KIRs in [102]).

#### Remarks on the presence of some probes in the genetic signature that discriminates Controls from PPMS patients

FOXO3 [103]–[106] is downregulated in many of the PPMS samples. Deficiency in FOXO3 leads to spontaneous lymphoproliferation, associated with inflammation of several organs, in the absence of overt apoptotic defects [107]. FOXO3 regulates, by inhibiting NF-kappaB activity [108], the tolerance and activation of T cells and is proposed as an inhibitor of inflammatory transcriptional activity [107].

An interesting finding in this signature is the presence of a probe for HAR1A (highly accelerated region 1A (non-protein coding)). HAR1A was first identified in 2006 by Katherine Pollard et al. as part of a novel “RNA gene” expressed specifically in Cajal-Retzius neurons in the developing human neocortex from 7 to 19 weeks of gestation [109] and co-expressed with reelin (which is an extracelluar matrix protein that is considered essential in the laminar organization of structures in the brain during development). Panteri et al. have reported that reelin is secreted by Schwann cells in the developing peripheral nerve, is dowregulated in adult stages, and induced following sciatic never injury. They have proposed reelin as a factor in determining the final calibre of myelinated axons and the absolute number of fibers per unit area in the peripheral nervous system [110]. What makes HAR1A somewhat unique is that it is one of the “highly accelerated regions” [111], where evolutionary mechanisms have created pressure for the emergence of “human-specific” brain features. HAR1A has been found by whole genome bioinformatics comparisons and is at centre stage of research on the functional genomic implications of these new non-coding RNA structures [109], [112]. In the present study, HAR1A is one of two highly accelerated regions that we point to as interesting putative biomarkers; the other is in a protein-coding region and is discussed later. We highlight, however, the presence of SNHG5 (small nucleolar RNA host gene 5 (non-protein coding)), located on chromosome 6q15 [113], [114], in this signature. Both non-protein-coding probes warrant further investigation into the role that they may have in neurological diseases, especially MS.

Other notable biomarkers include CD52, RNASE2, and EGR3 (upregulated in PPMS in comparison to Controls). The CD52 molecule (Cambridge pathology 1 antigen, CAMPATH-1 antigen) [115]–[123] is targeted by Alemtuzumab and there are recent reports that indicate that it may be an effective treatment for early MS [124]. RNASE2 (ribonuclease, RNase A family, 2 (liver, eosinophil-derived neurotoxin)) [125], [126] is linked to selective neuronal and axonal damage to white matter of cerebellum and spinal cord of experimental animals when injected intrathecally (the “Gordon phenomenon”) [127]. The upregulation of EGR3 has apparently never been previously reported – neither in PPMS nor in other clinically defined subtypes of MS – and thus could be an important finding. EGR3 is a member of the KROX/EGR family of genes that code for immediate-early transcription factors [128], [129], which regulate thymocyte proliferation during the transition from CD4−CD8− to CD4+CD8+ [130], and with EGR2 is a negative regulator of T-cell activation [131] and development (see [132]–[134]). Experiments in mice show that together with EGR1/KROX-24 and SOX10, it cooperates to activate the Myelin Protein Zero (Mpz) intron element by EGR2 [135]. A common pathway for developmental regulation of Mpz and other peripheral myelin genes has been proposed [135]. EGR3 was also linked to myelination in Schwann cells, hence dysregulation of these concerted pathways may have consequences for neuropathies [136].

#### Remarks on the presence of some probes in the genetic signature that discriminates Controls from RRMS patients

We again note the presence of a probe for LOC649143 (similar to HLA class II histocompatibility antigen, DRB1-9 beta chain precursor (MHC class I antigen DRB1*9) (DR-9) (DR9)). Another probe that seems to be consistently upregulated in controls but not in MS samples is for HLA-DQB1 (major histocompatibility complex, class II, DQ beta 1). This is of potential interest in view of the strong evidence that the expression of the MHC class II Allele HLA-DRB1*1501 is regulated by vitamin D [137]. The analysis involved sequencing the vitamin D response element (VDRE) in more than 1,000 chromosomes from HLA-DRB1 homozygotes; they localised a single MHC (VDRE) to the promoter region of HLA-DRB1, while there was an important variation in non-MS-associated haplotypes [137]. We also observe a number of upregulated ribosomal protein genes (including RPS10, RPS14, RPL12, RPL24, etc) in this signature.

With a different behaviour, we should cite a probe for VDR (Vitamin D (1,25- dihydroxyvitamin D3) receptor) and, as in the C/MS signature, a probe for LOC650557 (HLA class II histocompatibility antigen, DQ(W1.1) beta chain precursor (DQB1*0501)), which are upregulated in MS. Other interesting biomarkers include CA2 (Carbonic anhydrase II) (downregulated in RRMS), IRF5 (Interferon Regulatory Factor 5) and CD3D (CD3d molecule, delta (CD3-T-cell receptor complex)), both upregulated in RRMS. CA2 is localized to oligodendrocytes, myelin, and choroid plexus epithelium in the human brain [138]. In 1996, Cammer reported delays on oligodendorcyte maturation in mutant mice deficient in CA2 [139], [140].

IRF5, SOD1, NF1 (neurofibromin 1), CD3D, ADAM-17 (ADAM metallopeptidase domain 17) and ILF2/NF45 (interleukin enhancer binding factor 2, 45 kDa), on the contrary, have a tendency to be upregulated in RRMS samples. An allele in IRF5 has been reported as conferring an increased risk for inflammatory bowel diseases, systemic lupus erythematosus and MS, suggesting that there is a link between IRF5 and several autoimmune diseases [59], [141]. SOD1 and NF1 and their homologues have appeared in our gene ontology search as linked with several types of myelopathies and familial amyotrophic lateral sclerosis [142]–[151]. NF1 is expressed in cortical neurons and oligodendrocytes and sensory neurons and Schwann cells in the peripheral nervous system [152]–[154]. There are anecdotal clinical reports of neurofibromatosis type 1 and MS co-ocurring in patients [155], [156]. OMgp, the oligodendrocyte-Myelin glycoprotein gene, is within an intron of NF1 [157]. CD3D has a tendency to be upregulated in RRMS samples and is involved in TCR activation [158] and T-cell development [159]–[161]. ADAM-17 has been observed to be upregulated in MS lesions in brain tissue [162].

#### Viral Pathways are Significantly Overrepresented in the genetic signature that discriminates between Controls and PPMS patients

Each signature was also functionally profiled using g:PROFILER [163], which integrates several profiling resources, including Gene Ontology, KEGG and Reactome pathways, and TRANSFAC sequence motifs. Only three of the signatures, C/MS, C/RR, and C/PP, had profiles containing significant terms. However, g:PROFILER's significance test is considered to be very conservative, so profiles derived under a less stringent significance criterion, *p*-value <0.005, are provided (supporting information Table S6).

There is a group of nine ribosomal genes (RPL27, RPL35, RPL35A, RPL39, RPS5, RPS14, RPS15A, RPS27A, and RPLP0P2) in the C/PP signature that are responsible for almost all the terms in its profile (see Table S6). Most of these terms relate to influenza or translation Reactome pathways – principally translation initiation, translation elongation and influenza viral RNA transcription and replication. Although influenza itself is considered unlikely to be involved in MS, other viruses have been linked to autoimmune diseases. One viral theory of MS relates genetic sequences for viruses to similar sequences that code for the myelin sheath [164]. T cells adapted for the protein products of the virus sequence could mistakenly recognize myelin proteins as antigens and, were they to penetrate the blood-brain barrier, attack them in the brain [165], [166]. Some viruses, including influenza, do contain genetic sequences that mimic human myelin protein coding sequences [164]. Furthermore, several studies have linked exacerbation of MS lesions to viral infection [167]–[171]; however, the studies usually target the relapsing forms of the disease rather than the primary progressive form, which is where we find the connection to these ribosomal proteins linked to a viral pathway.

### Analysis of Differential Gene Co-Expression

**Figure 11:**
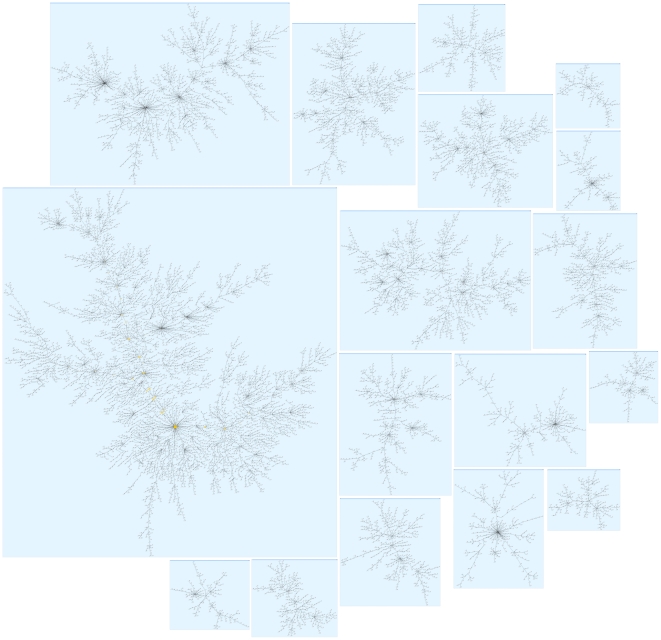
The seventeen largest clusters. The clustering results from using a distance measure based on changes in correlation of gene expression between classes.

**Figure 12:**
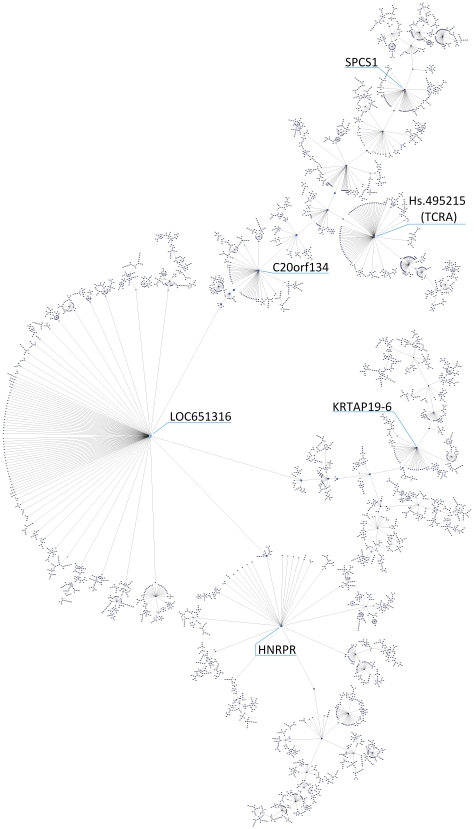
The largest cluster from **Figure 11** with a different layout. Nodes represent probes, colour and size indicate its centrality in the graph (number of connected nodes). Some of the prominent “stars” have been labelled.

In this section, we report on the results of applying our second method of differential gene (co-)expression analysis. A *kNN-MST* clustering algorithm [172], [173] was applied to the detection p-value prefiltered whole genome mRNA expression data set (19,000 probes) to produce a clustering of nodes. More formally, the clustering result is a forest, a collection of disjoint graphs such that each component is a tree (a connected graph with no cycles). Each cluster corresponds to a particular tree in the forest. Each node corresponds to an mRNA probe sequence. Nodes are connected to its nearest neighbour according to a distance metric based on pair-wise differential co-expression (see Materials and Methods section for details). A pair of nodes whose expression is perfectly correlated in control samples and perfectly anti-correlated in MS samples (or vice versa) has a distance of zero; the distance increases as expression in controls and MS correlates better. In total, 448 clusters were found, 22 of which contain more than one hundred nodes. Figure 11 shows the seventeen largest clusters, one of which is depicted in greater detail in Figure 12 (Figure 11 contents are provided as a graphics markup language file in supporting information Figure S1).

**Figure 13:**
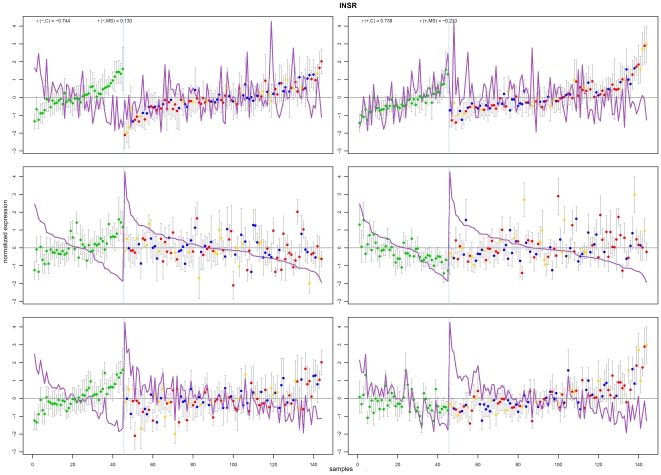
Correlation plot for the insulin receptor star centre (INSR). The figure shows the correlation of scaled expression values of INSR (insulin receptor, purple line) with the average of negatively (left column) and positively (right column) correlated neighbour probes (coloured dots). The light blue vertical line separates control samples to the left and MS to the right. The figures show three different orderings: by the harmonic mean of neighbours (top row), by the star centre expression value (middle row) and by the difference between star centre and neighbours average (bottom row). The class correlation values of star centre with neighbours are shown in the top figure; for example, r(−,C) is correlation coefficient of negatively correlated probes with control samples and r(+,MS) is the correlation of positively correlated probes with MS samples. Dot colours indicate sample class: green – control, blue – PPMS, red – RRMS, yellow – SPMS. This graph illustrate patterns of differential co-expression in Control versus MS samples. In particular, on the left it can be observed that some neighbours are strongly anti-correlated with the star centre in controls (correlation coefficient −0.744) but weakly correlated in MS (0.130); on the right, the remaining neighbours are strongly correlated with the centre in controls (0.739) but weakly anti-correlated in MS (−0.233).

Of particular interest are nodes with many immediate neighbours – a configuration that we call a ‘*star*’. Each star contains a designated *centre* node and its immediate neighbours; a high cardinality star has a centre with at least twenty neighbours. A star indicates that a group of genes exhibit differential co-expression between two different class labelling; in this case to the control and MS groups only. The co-expression of the centre with each neighbour (inversely) correlates over one sample group but not the other. To illustrate, a plot of the correlation behaviour of one star centre (a probe for INSR, Insulin Receptor) with its neighbours is shown in Figure 13. In total, there were 39 high cardinality stars; the members of these stars and their correlation plots are given in supporting information Table S7 and Figure S2, respectively.

**Figure 14:**
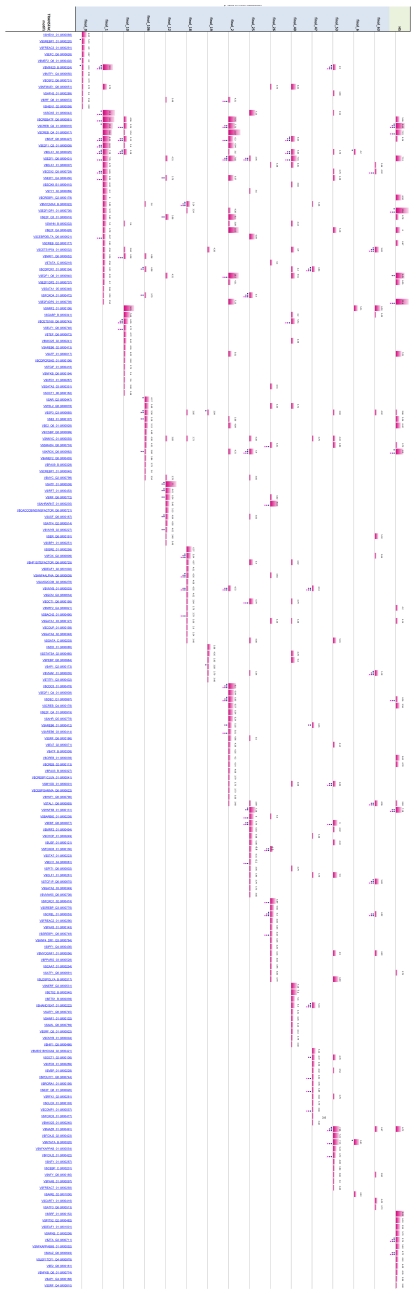
TRANSFAC motif profiles for the fifteen clusters found by correlation clustering (cyan bars). Only “key” regulators of each profile, which are found by hitting set analysis (see figure legend), are shown. The TRANSFAC profile for the set containing all the key regulators' coding genes is shown (orange bars); this profile in effect contains “higher level” regulators – regulators of regulators. Size of bar is proportional to the negative natural logarithm of *p*-value, which is also given as a number. Only significant motifs (p-value ≤0.05) are shown.

Profiles containing overrepresented regulatory sequences were obtained for the 39 high cardinality stars. The members of the stars were assigned to one of 15 groups according to the cluster containing the star, which were profiled for overrepresented TRANSFAC binding motifs using GATHER [95]. Figure 14 shows the complete profiles for the 15 groups. The three most significant transcription factor motifs are for SOX5 (V$SOX5_01), the CREB family (V$CREB_Q4_01), and the CREB and ATF families (V$CREBATF_Q6), all with similar p-Value <6.6E-6. Again, we also obtained higher-level regulatory sequences (Figure 14, HS column, and Table 1); the two most significant were for the E2F-1:DP-1 heterodimer (V$E2F1DP1_0) and the E2F-4:DP-2 heterodimer (V$E2F4DP2_01), with p-Values <3.31E-6.

**Figure 15:**
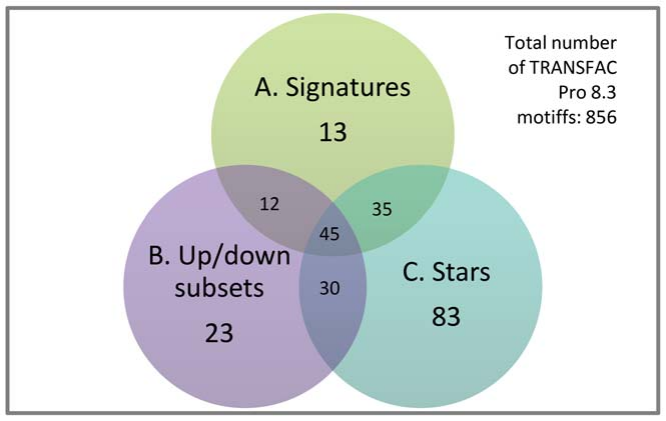
Number of motifs found in common between the different analytical approaches. Number of motifs occurring in the A. signature profiles, B. up/down regulated signature subset profiles, and C. cluster profiles.

A significantly large proportion of these motifs intersect with those in the TRANSFAC profiles of the signatures (see Figure 15). In particular, 80 motifs – or 78.4% of the signature motifs and 41.5% of the star motifs – occur in at least one star profile and one signature profile (the expected likelihood of this event under uniform random selection of motifs is less than one in 10^9^, i.e. *p-*value <10^−9^). A similar proportion occurs in the intersection between the star and up/down regulated signature subset profiles: 75 motifs, or 77.3% of the up/down signature subset motifs and 38.9% of the star motifs (again: *p-*value <10^−9^).

The significant agreement between the two approaches is notable because patterns of differential expression and differential co-expression are unlikely to contain a large number of the same genes. This is indeed the case; there are 790 and 1,256 unique probes in the signatures and stars respectively, only 53 of these (6.7% and 4.2% respectively) occur in the intersection. The agreement in the regulatory motifs between both approaches could be explained by the occurrence of transcriptional dysregulation in MS, mediated by the transcription factors associated with these particular regulatory sequences.

#### Remarks on the Composition of the set of “stars”

We now highlight some of the probes that correspond to “stars”, seem to indicate genes that are differentially co-expressed in MS patients.

ASPM (ASP (abnormal spindle) homolog, microcephaly associated (Drosophila)) is a centrosome protein [174] which initially was considered to be a major determinant of cortical size [175], but its function is now revisited [176]–[179], however its relatively recent positive selection status remain unquestioned. ASPM is also a highly accelerated region that holds key features that make us human [112], including perhaps language [180]–[182]. Deficits in ASPM's functions has been consistently associated with neurodevelopmental disorders, primary in microencephaly [183]–[188].

PTPRN (protein tyrosine phosphatase, receptor type, N), appears in the literature with different names (IA2, IA-2, IA-2/PTP, ICA3, ICA512, ICA 512, Islet cell antigen 512, Islet cell autoantigen 3). The literature reports associations with insulin dependent diabetes mellitus [189]–[194] and influences not only the secretion of hormones but also of neurotransmitters [195], brain development [196]. Disruption of PTPRN results in alterations in insulin secretion and glucose tolerance [197].

A probe for INSR, the Insulin Receptor (probe ILMN_1670918), has lost its pattern of co-expression with a group of 20 other probes. If the INSR has lost co-expression in MS with some other partner probes we should expect downstream effects on the insulin signaling pathway. Indeed, MAPK3 also belongs to the insulin signaling pathway and has also been identified at the centre of a star with 23 neighbours. It is noteworthy that GATHER brings *Insulin signalling* pathway (KEGG hsa04910, p-Value <1E-4, Bayes 5) as the most significant when all neighbours of the 39 stars are searched; 17 out of the 21 probes that appear in the insulin signalling pathway (including INSR and MAPK3 themselves) have V$KROX_06 as a binding motif, and all 21 have V$E2F_Q6, pointing again to the KROX family of proteins and E2F, now linked to insulin signalling regulation.

The star corresponding to UniGene HS.495215 (T-cell antigen receptor alpha (TCRA), probe ILMN_1820392) has 56 neighbours. Of these 56, 15 have also appeared in one of our seven genetic signatures. With the exception of only one, they have appeared in either the signatures C/PP or C/RR. There is a relative big number of ribosomal proteins associated (RPL24, RPL27, RPL35, RPS10, RPS14, RPS17 (Ribosomal proteins L24, L27, L35, S10, S14, S25, S17), LOC441246 (ribosomal protein L35 pseudogene 5), LOC285900 (ribosomal protein L6 pseudogene 20), LOC646483 (ribosomal protein L6 pseudogene 19). We have also found CD3D (CD3d molecule, delta (CD3-TCR complex)), GNL3/Nucleostemin (guanine nucleotide binding protein-like 3 (nucleolar)) SNRPD2 (small nuclear ribonucleoprotein D2 polypeptide 16.5 kDa), SEC11A/Endopeptidase SP18 (SEC11 homolog A (S. cerevisiae)), TOMM7 (translocase of outer mitochondrial membrane 7 homolog (yeast)). The only downregulated gene (in a signature SP/PP) is MAD2L1BP (MAD2L1 binding protein). From this group, it is perhaps CD3D and its the relationship with the TCRA locus that caught our immediate attention, since progress in the understanding of CD3D's functions come from studies on its deficiency, defects and/or inactivation [160], [198]–[204]. CD3D is a receptor involved in TCR activation [158] which has been recently found to be a biomarker for chronic graft-versus-host disease in mononuclear cell samples from allogeneic hematopoietic stem cell transplantation recipients [205] and primary Sjögren's syndrome [206]. CD3D promotes the progression of early thymocytes towards the double positive-stage [160], [207], [208]. GNL3/Nucleostemin, is a gene implicated in cell cycle/growth/progression [209], [210], neural progenitor activity [211], which has also been linked to bipolar disorder [212]. However, upregulation of GNL3 may also lead to apoptosis [213].

Interestingly, of the genes present in the MS signature only three, two of which are ribosomal proteins, occur in any of the 39 highest cardinality stars (all three are neighbours rather than star centres). Of the 790 genes differentially expressed in some signature, three are also star centres (PTPRN, underexpressed in C/RR; C20orf134, an uncharacterized protein overexpressed in C/PP, and Hs.443993, another uncharacterized transcribed locus overexpressed in PP/SP), while fifty appear as neighbours of star centres. Of these 53 genes, sixteen (30%) appear in a single star, the star with centre Hs.495215 (T-cell antigen receptor alpha locus, TRAV38-1), most of which come from C/PP and C/RR. The TRAV38-1 star is strongly associated with ribosomal proteins.

## Discussion

In this paper we have analysed gene expression data with a variety of computational methods with the aim of uncovering transcription factors that potentially dysregulate in MS or one or more of its disease subtypes. Table 1, Table 2 and Table 3 summarise the most significant transcription factors uncovered by our methods. Of these, the most significant are KROX/EGR family members, ATF2, YY1 (Yin and Yang 1), E2F-1/DP-1 and E2F-4/DP-2 heterodimers, SOX5, DEAF1, and CREB and ATF families.

**Table 3:** The 25 most significantly overrepresented binding motifs/transcription factors that have coding genes involved in T cell specification.

	Binding motif	Transcription factor	Profile	Gene(s)	*p*-value
1.	V$KROX_Q6 (M00982)	EGR family	C/P+C/R(−)	EGR1, EGR2, EGR3	3.31E-06
2.	V$YY1_02 (M00069)	YY1	C/P+C/R(+)	YY1	1.65E-05
3.	V$KROX_Q6 (M00982)	EGR family	C/R(−)	EGR1, EGR2, EGR3	6.25E-05
4.	V$KROX_Q6 (M00982)	EGR family	HS	EGR1, EGR2, EGR3	2.03E-04
5.	V$YY1_02 (M00069)	YY1	C/P(+)	YY1	2.41E-04
6.	V$KROX_Q6 (M00982)	EGR family	C/R+C/S(−)	EGR1, EGR2, EGR3	4.62E-04
7.	V$ER_Q6 (M00191)	ER	All genes	ESR1	4.62E-04
8.	V$CETS1P54_01 (M00032)	C-ETS-1(P54)	RR/SP	ETS1	8.67E-04
9.	V$E2F_Q3_01 (M00918)	E2F	All genes	TFDP1	1.84E-03
10.	V$CDP_01 (M00095)	CUTL1	C/P+C/R(−)	ETS1	2.09E-03
11.	V$YY1_02 (M00069)	YY1	C/P+C/S(+)	YY1	2.22E-03
12.	V$EBF_Q6 (M00977)	EBF	final_53	EBF	2.48E-03
13.	V$ER_Q6 (M00191)	ER	C/P+C/R(+)	ESR1	2.63E-03
14.	V$KROX_Q6 (M00982)	EGR family	final_25	EGR1, EGR2, EGR3	2.74E-03
15.	V$ER_Q6 (M00191)	ER	C/MS	ESR1	2.74E-03
16.	V$OCT1_Q6 (M00195)	OCT1	final_25	POU2F1	3.06E-03
17.	V$EBF_Q6 (M00977)	EBF	final_25	EBF	3.21E-03
18.	V$KROX_Q6 (M00982)	EGR family	HS	EGR1, EGR2, EGR3	3.21E-03
19.	V$CETS1P54_01 (M00032)	C-ETS-1(P54)	final_63	ETS1	3.38E-03
20.	V$TCF1P_Q6 (M00670)	TCF1	final_63	YY1	3.38E-03
21.	V$KROX_Q6 (M00982)	EGR family	C/P(−)	EGR1, EGR2, EGR3	3.38E-03
22.	V$IK3_01 (M00088)	IK-3	PP/SP	AIOLOS	4.99E-03
23.	V$KROX_Q6 (M00982)	EGR family	All genes	EGR1, EGR2, EGR3	5.92E-03
24.	V$E2F_Q3_01 (M00918)	E2F	C/P+C/R(−)	TFDP1	5.92E-03
25.	V$ER_Q6 (M00191)	ER	C/S(−)	ESR1	6.22E-03

**Profile** identifies the TRANSFAC profile (‘HS’ is the hitting set profile for the signatures, see Figure 9; ‘All genes’ is the profile for the union of all genes in the C/PP, C/RR, C/SP signatures. **Gene(s)** gives the corresponding T cell specification regulatory genes identified in [275], [276].

### The YY1 transcription factor (Yin and Yang 1)

The zinc finger protein YY1 [214] can be a transcriptional activator or repressor of several genes (including those for ribosomal and nuclear-encoded mitochondrial proteins [215], [216]) and has known roles in replication, cell proliferation, differentiation, and developmental processes [214], [217]–[220]. It also regulates T-cell cytokine gene expression [221] and the immune response [222], [223] and its relationships with viral replicating processes [224]. Experiments in mice seem to indicate that the T-cell specific expression of CD3delta is regulated, in part, by YY1 which represses the promoter activity strongly in non-T cells [225]. The disruption of Epstein-Barr virus latency in B cells is induced by expression of BZLF1 immediate-early protein, and this transcription is negatively regulated by YY1 [226], [227]. A motif for inhibitory activity of YY1 on the PrP gene has been observed. The multiple functions of the YY1 transcription factor in the nervous system have been recently reviewed [226], [228], and enhanced transcription of the proteolipid protein (PLP), one of the most abundantly expressed myelin proteins, has also been observed [229].

YY1 inhibits human papillomavirus (HPV) replication in vitro, and binds the human papillomavirus-6 E1 promoter [230]. It has been suggested as a negative regulator in the differentiation-induced HPV-6 E1 promoter and the HPV life cycle (see also the link between YY1 and the long control region of HPV-16, as reported in [231]). Nearly half of the intermediate and late gene transcriptional promoters of vaccinia virus have a binding site for the YY1 transcription factor that overlaps the initiator elements, acting as a negative regulator [232]. It also seems to be implicated, together with SP1 and SP3, on the establishment or reactivation of varicella-zoster virus latency [233]. The non-structural protein NS of the Rift Valley fever virus interacts with the host protein SAP30 and with YY1, and as a consequence antagonize interferon-beta gene expression [234]. The Hepatitis C virus core interacts with the C-terminal end of the nucleolar phosphoprotein B23, and the recruitment of p300 reducing the repression effect of YY1 [235].

YY1 is considered a critical regulator of early B-cell development [236]. The perturbed expression of YY1 in the development of acute myeloid leukemia has been also highlighted by Erkeland *et al.* who observed that these gene tends to harbour viral integrations of the 1.4 strain of Graffi murine leukemia virus [237].

The role of overexpression of YY1 in the differentiation of oligodendrocyte progenitors has been studied in [238], conditional ablation of YY1 in the oligodencrocyte linage gives phenotypes which have defective myelination, ataxia and tremor. YY1 is then seen as a repressor of the transcriptional inhibitors of myelin gene expression (Tcf4 and Id4), by a mechanism that includes the recruitment of HDAC1 to their promoters during oligodendrocyte differentiation [239].

In summary, YY1 transcription factor seems to be playing a key role in the pathogenesis of MS or its subtypes, given its connection to processes affecting myelin protein generation, viral replication and immune response processes. It appears as one the most significant transcription factors related to the differential expression of genes in MS patients.

### The EGR/KROX family

A motif for the Early Growth Response family (KROX/EGR family motif, V$KROX_Q6) was one of the most significantly overrepresented motifs identified by this study. A member of this family, EGR2/KROX-20 has been previously associated with the regulation of Schwann cells [240] (which in turn are known to be affected in some demyelinating disorders [241] – see also [242], [243]). In Schwann cells, progesterone has also been shown to stimulate KROX-20 expression [244]–[249].

Activation of KROX-20 is an indispensable transcription factor for myelination and a prerequisite for Schwann cell differentiation” [250]. EGR1 and EGR3 (we have already observed the later as upregulated in PPMS in comparison to Controls) provide a balance to EGR2/KROX-20 in Schwann cells. It has been hypothesised that the pair should be expressed prior to start myelination, be downregulated on myelination, but be again upregulated as Schwann cells dedifferentiate after injury [136] (see also [251]–[253]). There exists an antagonist effect of EGR1 and EGR2 in Schwann cells [254]. EGR1 has been previously found disregulated in a genome-wide expression profiling analysis of synchronously differentiating olygodendrocytes generated from pure precursor cells in vitro, and comparing with the program of cells in vivo. In that study, a probe for EGR1 was found to be the most strongly expressed specifically in undifferentiated oligodendrocyte precursor cells [255]. A possible joint dysregulation of members of this family could be explored to see if they have a role in the differentiation of oligodendrocytes [256], [257].

As it was the case of the YY1 transcription factor, this family role in the immune response has to be monitored as there are reports of EGR/KROX being negative regulators of T cell activation [131], having roles in T cell anergy [133], [258]–[261], upregulation of Fas ligand [262] and TNF [263], CD154/CD40 ligand on CD4 T cells [264], autoreactive T cell activation [265], upregulation of cell cycle inhibitors p21(cip1) and p27(kip) (as studied in tolerant T cells following antigen challenge in vivo), interact with NF-kappaB p50 and p65 [266], regulate proinflammatory cytokines [267], as well as thymocyte development [268], [269].

### ATF2 and SOX5

Studies of homozygous shiverer mutant mice have identified ATF2 as a regulator of the expression of MAG expression at a specific stage of shi/she oligodendrocyte differentiation [270]. It was reported in 2005 that infection of macrophages by Theiler's murine encephalomyelitis virus induces a type of demyelination in mice that that that resembles human MS, and also observed induction of ATF2 30 minutes after the infection of macrophages [271]. A study of a mycobacterial antigen suggests that ATF-2 might control expression of CREB and c-Jun during T cell activation and that of expression of IFN-gamma [272].

Another transcription factor motif significantly overrepresented is SOX5 (p-value <6.59E-6). Together with SOX6, SOX5 was identified as cell-autonomously regulating several stages of oligodendrocyte development in the mouse spinal cord (via repression of specification and terminal differentiation and influence migration patterns) [273].

### KROX/EGR Family and YY1 are Further Indicated by Involvement in T Cell Specification

It is widely believed that T cells are central to the pathogenesis of MS; autoreactive T cells are hypothesised to coordinate an attack against central nervous system myelin, triggering an inflammatory response leading to neurological damage. However, the origin of the autoreactive lymphocytes is unknown. We looked for a correspondence between the regulators that we found above and those of T cell specification (the process by which haematopoietic stem cells progress through intermediate progenitor cell types to mature T cells), which may help to identify a defect in the T cell developmental program in MS. Such defects have been linked to many diseases, including a proposed contribution to diabetes susceptibility in non-obese diabetic mice, with CD4+CD8+ blasts that have abnormally high expression of IL-7Ralpha, and c-Kit [274].

Cell specification is well understood for T cells compared to most other cell types and many regulators of the process have been identified. In two recent comprehensive studies, a sizeable network of regulatory relationships involved in T cell specification was constructed [275] and the expression of more than 100 regulatory genes characterized in early T cell progenitors [276]. Table 3 lists the transcription factors that are known to participate in T cell specification [275], [276] and which are indicated by our above analyses of differential expression as potentially playing a role in the pathogenesis of MS. The most significant are the KROX/EGR family and the Yin and Yang 1 transcription factor, which are both indicated in several different signatures and clusters.

### YY1, IKAROS and other “legacy transcription factors” are relatively unchanged during early T-lymphocyte specification and commitment

T-cell lineage differentiation is an ongoing process in mammals with a clear transcription factor “armature” regulation [277] that is currently being thoroughly mapped [275], [278], [279]. Defects on the T-cell developmental program have been linked to many diseases, including a proposed contribution to diabetes susceptibility in non-obese diabetic mice, with CD4+CD8+ blasts that have abnormally high expression of IL-7Ralpha, and c-Kit [274].

To obtain a time-series of early T-cell development for gene expression analysis, David-Fung et al. sorted subsets of immature mouse thymocytes from postnatal, weanling mice. Stages of development were based on the expression of CD44, CD25, and the growth factor receptor c-Kit and markers CD44, CD25 and the heat-stable antigen (CD24) and CD27. In the first stages of T-cell development the cells that enter the thymus are defined as “double negative” (DN), since they do not have the T-cell markers of maturity (CD4 and CD8). David-Fung et al, indexed these stages in terms of increasing maturity as DN1 (or early T-cell precursor, ETP), DN2, DN3a, DN3b, and DN4. Using four independent series of samples (from normal, young adult mice), DN1, DN2, total DN3, and DN4 cells were collected in each of the first two sets (“sets 1 and 2”, Series A). In the second two sets (“sets 4 and 5”, Series B) the DN3 cells were subdivided into DN3a (pre-selection) and DN3b (the first stage past β-selection).

YY1, together with Ikaros (Ikzf1), MYB and other transcription factors have been shown to have minimal changes from DN1 to DN3 stage. The list of other transcription factors that have critical roles in T-cell differentiation and development, and that have minimal changes of gene expression also includes MLL1, Gfi-1, GABPα, Stat5b, Oct1 (Pou2f1), and TOX. More recently, David-Fung et al have included YY1 together with FOG-1 (Zfpm1), Gse1, and Zfp598 to this list of “legacy genes” [276]. This is in sharp contrast with other genes that have large changes in T-lineage gene expression. Examples include Aiolos (Ikzf3), Gata3 [280], [281], and others that have clear patterns of upregulation during DN1 to DN3 progression (for the complete list see [276]).

### A Hypothetical Network of Transcription Factors that Dysregulate genes in MS

If transcription factors do dysregulate genes in MS, it is reasonable to suspect that some are only associated with specific disease subtypes. Based on our data, we propose the following speculative, informal initial model that links transcription factors to MS disease subtype. The model is limited to transcription factors currently known to be involved in T cell specification [275]. It is informally constructed with the transcription factors indicated by the TRANSFAC analysis of the signatures (see Figure 9), which distinguish between disease subgroups or between controls and disease subgroups.

**Figure 16:**
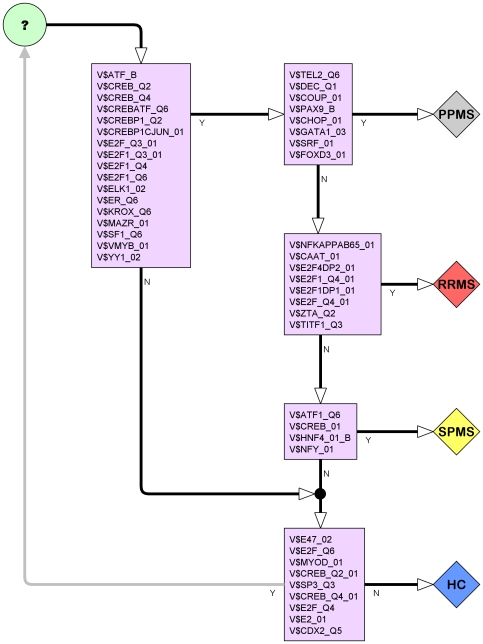
Speculative initial sketch for a model of transcription factors currently known to be involved in T cell specification and which may also be involved in the pathogenesis of MS, showing links to disease subtype. Other transcription factors that also need to be added to this model are those which we have found very relevant in our analysis such as the Early Growth response EGR/KROX family, ATF2, YY1 (Yin and Yang 1), E2F-1/DP-1 and E2F-4/DP-2 heterodimers, SOX5, and CREB and ATF families.

The model is illustrated in Figure 16. Each node in the model lists transcription factors that dysregulate in one of the node's outcomes. Under the model, dysregulation by CREB/ATF, E2F families, ELK-1, MAZR and principally by KROX, V-MYB and YY1 characterises MS overall. Dysregulation by CHOP, COUP-TF, HNF4 heterodimer, DEC, FOXD3, GATA-1, PAX9-B, SRF and TEL-2 characterises PPMS. CCAAT box, E2F-1/DP-1 and E2F-4/DP-2 heterodimers, NF-kappaB (p65), TTF-1 and Zta characterises RRMS. Finally, ATF-1, HNF-4alpha1 and NF-Y are the transcription factors associated with SPMS. Lack of evidence of dysregulation by the afore mentioned groups, but dysregulation by CDX-2, E2, E47, MyoD and SP3 might indicate that exclusion from MS cannot be asserted. Note that under this model SPMS has the least characteristic group of transcription factors, and there is some overlap with MS as a whole.

The model is intended as a starting point to motivate further research for identifying transcription factors involved in the pathogenesis of MS, as it relies on simplifying and biologically naïve assumptions – for instance, T cell specification does not occur in peripheral blood, where the samples were collected, but in the thymus, but effects on peripheral blood are still measurable. Thus, there is room for refinement and it could be extended also by considering other transcription factors such as the key regulators identified by the hitting set analyses. However, our hope is that this model can be used to corroborate other findings, single out transcription factors for more detailed research, and participate in unravelling some of the heterogeneity in MS.

### Conclusions

Using state-of-the-art bioinformatics methods, our analysis uncovered a network of transcription factors that putatively contribute to the dysregulation of several genes in MS or one or more of its disease subtypes. Separate analytical methods were used on the same dataset, involving sophisticated mathematical algorithms to solve combinatorial optimization problems. We noted a convergence of findings, leading to a set of transcription factors which are involved in the early T-lymphocyte specification and commitment as well as in oligodendrocyte dedifferentiation and development. The most significant transcription factors motifs were for the Early Growth response EGR/KROX family, ATF2, YY1 (Yin and Yang 1), E2F-1/DP-1 and E2F-4/DP-2 heterodimers, SOX5, and CREB and ATF families.

## Materials and Methods

### Ethics Statement

Institutional Human Research Ethics Committees approval to conduct this study was obtained by the Chief Investigators of the ANZgene Consortium, that included: E08/910 from Eastern Health Research and Ethics Committee (VIC), Research Application 105/07 from Flinders Medical Centre Research Ethics Committee (SA), GU Protocol HSC/09/03/HREC from Griffith University (QLD), MEC/07/12/176 from Multi-Region Ethics Committee (NZ), 05/04/13/3.09 from Hunter New England Human Research Ethics Committee (NSW), H-505-0607 from the University of Newcastle Human Research Ethics Comitee (NSW), HREC #1980 from The University of Sydney Human Research Ethics Committee (NSW) and H0009782 from the Human Research Ethics Committee (Tasmania) Network (TAS).

Written informed consent was obtained from all subjects prior to participation in this project.

### Patients, Phenotyping, and Inclusion Criteria

A cohort of 99 people with MS and 45 healthy controls was recruited from Sydney, Newcastle (NSW) and Gold Coast (QLD). Diagnosis of MS was confirmed using the revised McDonald criteria [282]. Further separation into RRMS, SPMS and PPMS cases was done according to published criteria [283], [284]. Patient demographics are given in Table 4. All patients had no immunomodulatory therapy for at least three months prior to sample collection.

**Table 4:** Demographics of patient and control cohorts.

Sample	N	M∶F	Age	Age at diagnosis	Duration	EDSS
**Controls**	45	16∶29	48.5 (23–77)	–	–	–
**MS**	99	33∶66	54 (28–97)	35.5 (16–59)	16.5 (1–54)	4.5 (0–9)
**PPMS**	43	21∶22	55.3 (30–87)	42 (21–58)	13 (5–31)	5.6 (2–8)
**RRMS**	36	7∶29	48.5 (29–65)	33 (19–56)	15 (1–36)	2.7 (0–6.5)
**SPMS**	20	5∶15	57.5 (34–73)	35.5 (16–59)	22 (2–54)	6.5 (4–9)

**M∶F** male:female; **Age, Age at Diagnosis**: Mean and range; **EDSS** (expanded disability scale): mean and range.

### RNA Extraction, Microarray Protocols

The peripheral blood samples were collected between 9AM and 1PM and total RNA was extracted from the whole blood in PAXgene tubes using PAXgene™ Blood RNA kit (Qiagen, Germany) according to the manufacturer's instruction unless otherwise stated. Total RNA quality was assessed and its concentration was measured using Agilent RNA 6000 series II Nano kit (Agilent Technologies, CA, USA). Minimum RNA Integrity Number (RIN) of >7 was employed for each sample to pass the quality assessment. 250 ng total RNA from each sample was biotinylated and amplified using Illumina® TotalPrep RNA Amplification Kit (Ambion, TX, USA). Total RNA was reverse transcribed to synthesize first strand of cDNA followed by second strand synthesis. Double stranded cDNA was then transcribed and amplified in vitro to synthesize biotin labelled complementary mRNA (cRNA). The cRNA yield was measured at 260 nm using NanoDrop 1000A Spectrophotometer. 750 ng of cRNA sample was hybridized on a human HT-12 expression beadchip (Illumina Inc., CA, USA) profiling 48,804 transcripts per sample. The chips were stained with streptavidin-Cye3 conjugate and scanned using an Illumina BeadArray Reader (Illumina Inc.).

The dataset is available in Gene Expression Omnibus under the reference GSE17048.

### Normalisation and Initial Pre-Filtering

The average signal intensity for each gene was measured using Beadstudio v3. The sample signals were normalized with cubic spline in order to minimize variation due to non-biological factors. Approximately 19,000 genes were selected for differential expression analysis with detection *p*-value <0.01 for at least one of all samples. The detection *p*-value measures the probability of observing signal without specific probe-target hybridization.

### Analysis of Differential Expression

A genetic ‘signature’ was obtained from the detection *p-value* filtered dataset for each pair of sample groups by applying an additional two-step filtering process, which removed probes lacking sufficient discriminative power according to information content and predictive ability respectively.

1. An entropy-based selection procedure employing Fayyad and Irani's multi-class, multi-interval discretisation algorithm [88] was applied. For each probe sequence, the algorithm finds the threshold that minimizes the class-information entropy of the samples, which discretises the dataset (greater than threshold – 1; less – 0). It then discards the genes that do not sufficiently discriminate between the sample groups according to the minimum description length principle.
2. Following, an (α,β) *k*-feature set selection method [89], [90] was applied. The (α,β) *k*-feature set problem asks, in this context, for the smallest subset of probe sequences containing: i) at least α probes differentially expressed (after discretisation) between every pair of samples in the Cartesian product of the two sample groups (a probe is differentially expressed between a pair if their values are 0 and 1), and ii) at least β probes with equal (discretised) value for all pairs over the Cartesian product of the sample groups. Intuitively, α and β specify the degree of inter-class differentiation and intra-class similarity respectively. In practise, we use α maximum and β maximal to facilitate computation of a solution; Table 5 gives the (α,β) values (and various summary statistics) for each signature. We refer the reader to [89], [91]–[93] for mathematical details and applications of the approach.

**Table 5:** Summary of (α,β) *k-*feature set selection results for each signature.

							0-Centered			1-Centered		
	#0	#1	# EntF	Nalfa	A_max_	#ABK	Nbeta	B_max_	Bmxml	Nbeta	B_max_	Bmxml
C/MS	45	99	369	4455	40	145	990	232	79	4851	107	58
C/PP	45	43	360	1935	66	221	990	209	113	903	170	113
C/RR	45	36	366	1620	73	260	990	235	154	630	160	131
C/SP	45	20	317	900	40	113	990	245	67	190	124	67
PP/SP	43	20	133	860	23	77	903	90	48	190	83	53
RR/PP	36	43	126	1548	25	86	630	82	55	903	75	49
RR/SP	36	20	113	720	24	66	630	66	32	190	80	48

**#0, #1** are the number of samples in class 0 and class 1 (for example, in the C/MS signature, class 0, Controls, has 45 samples and class 1, MS, has 99 samples). **#EntF** is the number of features (probes) satisfying the entropy filtering criterion. **Nalfa** is the number of alpha pairs. **A-max** is the maximum number of features for all alpha-pairs. **#ABK** is the size of the optimum solution (signature size). **Nbeta** is the number of beta pairs, considering only one class. **B-max** is the maximum number of features for all beta-pairs. **Bmxml** is the maximum number of features per beta-pair before the solution increases in size. **0-Centered/1-Centered r**efers to the search for in-class coherency for class 0 and class 1 respectively.

### Analysis of Differential Co-expression

A cluster map was constructed for the approximately 19,000 probe sequences found to be expressed in whole blood, using a *kNN-MST* graph clustering analysis [172], [173]. The map connects each probe to its nearest neighbour under the distance metric 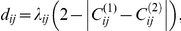
where *d_ij_* is the distance between probes *i* and *j*, *C_ij_* ^(*k*)^ ∈ [-1,1] is the correlation between *i* and *j* in class *k* (class 1 is controls and class 2 is MS, all subtypes), and



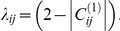
Correlation values are computed as a normalized sum of the Pearson and Spearman correlation coefficients, which is robust against outliers. The minimum distance between a pair of probe sequences is *d_ij_* = 0, which occurs when the expression of *i* and *j* are perfectly correlated over controls and perfectly anti-correlated over disease (or vice versa). A distance of *d_ij_* = 1, which is relatively short, occurs between a pair of probe sequences correlated (or anti-correlated) over controls and uncorrelated over disease.

With this definition of distance, high cardinality stars are particularly important since all (or almost all) the immediate neighbours of the centre have *changed or lost correlation with the centre in disease.* Under our working hypothesis, this effect is attributed to dysregulation of the transcription factor(s) associated with the centre *and not* with the neighbours.

### Hitting Set Filter

Our hitting set filter is formally defined as follows. Consider a set *S* (of TRANSFAC motifs), a collection *C* of *n* subsets *C*_1_,…,*C_n_* ⊆ *S*, and a positive integer *m*. The elements of *S* have positive weights *w_s_* (*p*-values), µ*s* ∈ *S*. We seek a subset *HS* ⊆ *S* such that |*HS* ∩ *C_i_*| ≥ *m, i  =  1,*…,*n*, and Σ_s_ *w_s_* is minimal. Such a set *HS*, if it exists, is a weight-minimal *hitting set* because it ‘hits’ all subsets *C_i_* at least *m* times.

To illustrate, consider the hitting set instance given in the matrix shown in Table 6 (left). Each subset *C_i_* is defined by *C_i_*  =  {*s_j_* | *M*(*i*, *j*)  =  ‘x’ }, where *M*(*i*,*j*) is the *i*,*j*th entry in the matrix with the row for *w* removed; for example, *C*_3_  =  {*s*_1_, *s*_3_, *s*_5_}.

**Table 6:** Hitting Set Example.

		*S*						
		*s*_1_	*s*_2_	*s*_3_	*s*_6_	*s*_5_	*m*	*HS*
	*w*_s_	5	6	6	7	8	1	*s*_1_
*C*	*C*_1_	x	x	x	x	x	2	*s*_1_, *s*_5_
	*C*_2_	x	x		x	x	3	*s*_1_, *s*_2_, *s*_3_, *s*_5_
	*C*_3_	x		x		x	4	No solution

On the right, weight minimal solutions are shown for *m*  =  1,…,4. Note that there is a two-fold effect as *m* grows: first, |*HS*| tends to increase with *m*; but second, beyond a certain point no satisfying *HS* exists. Throughout this paper, *m*  =  1, 2, and 3.

We employed CPLEX, an integer linear programming tool, to efficiently compute solutions to the hitting set instances. The hitting set problem can be formulated as an integer linear programming problem as follows
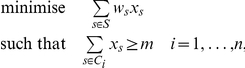
where *x_s_*  =  1 if *s* ∈ *HS*, *x_s_*  =  0 otherwise.

## Supporting Information

Table S1 Signature Control - MS. Heatmap and annotation for the C/MS signature. Probe Ids, expression values, hitting set annotation, and gene annotations are given.
(1.08 MB XLS)

Table S2 Signatures Control - RRMS and Control - SPMS. For each signature, the probe Ids, expression values, heatmap, hitting set annotation, and gene annotations are given.
(1.88 MB XLS)

Table S3 Signatures Control - PPMS, PPMS - SPMS, RRMS - PPMS and RRMS - SPMS. For each signature, the probe Ids, expression values, heatmap, hitting set annotation, and gene annotations are given.
(2.27 MB XLS)

Table S4 (α,β) k-feature set summary statistics. Summary statistics of the (α,β) k-feature set selection process for each of the signatures and combined listing of all signatures, showing over or under expression and signature overlap. Overlap with the signatures in the companion study are also given.
(0.52 MB XLS)

Table S5 TRANSFAC Profiles. Excel file containing the TRANSFAC profiles for the 7 signatures, 14 up/down-regulated signature subsets, and 15 star clusters.
(0.30 MB XLS)

Table S6 g:Profiler functional profiles for signatures. Functional profiles for each signature. Profiling was performed with g:PROFILER [163], which integrates several profiling resources, including Gene Ontology, KEGG and Reactome pathways, and TRANSFAC sequence motifs. Note that a group of six ribosomal protein genes (RPL10A, RPL12, RPL39, RPS17, RPS21, and RPS25P8) are responsible for over half of the significant terms (17 of the 30) in the C/MS profile, and are linked to viral and translation pathways. Similarly, there is a group of nine ribosomal genes (RPL27, RPL35, RPL35A, RPL39, RPS5, RPS14, RPS15A, RPS27A, and RPLP0P2) that are responsible for almost all the significant Reactome pathway terms in the profile for C/PP; most of these terms relate to influenza or translation pathways - principally translation initiation, translation elongation and influenza viral RNA transcription and replication.
(0.47 MB PDF)

Table S7 Members of high cardinality stars. Complete member listing of all 39 high cardinality stars.
(0.29 MB XLS)

Figure S1 Stars correlation plots. Correlation plots for all high cardinality stars.
(8.18 MB PDF)

Figure S2 Largest clusters. The seventeen largest clusters found by analysis of genes differentially co-expressed over controls and MS. Figure is in GML format.
(0.44 MB ZIP)
