# Supplementary material for: A Transcription Factor Map as Revealed by a Genome-Wide Gene Expression Analysis of Whole-Blood mRNA Transcriptome in Multiple Sclerosis
Source: PLoS One. 2010 Dec 1;5(12):e14176. doi: 10.1371/journal.pone.0014176 (PMC2995726; doi:10.1371/journal.pone.0014176)
Supplement: Table S6 — g:Profiler functional profiles for signatures. Functional profiles for each signature. Profiling was performed with g:PROFILER [163], which integrates several profiling resources, including Gene Ontology, KEGG and Reactome pathways, and TRANSFAC sequence motifs. Note that a group of six ribosomal protein genes (RPL10A, RPL12, RPL39, RPS17, RPS21, and RPS25P8) are responsible for over half of the significant terms (17 of the 30) in the C/MS profile, and are linked to viral and translation pathways. Similarly, there is a group of nine ribosomal genes (RPL27, RPL35, RPL35A, RPL39, RPS5, RPS14, RPS15A, RPS27A, and RPLP0P2) that are responsible for almost all the significant Reactome pathway terms in the profile for C/PP; most of these terms relate to influenza or translation pathways - principally translation initiation, translation elongation and influenza viral RNA transcription and replication. (0.47 MB PDF) [file pone.0014176.s006.pdf]

C-MS

Sig.	*p*-value	T	Q	T&Q	T&Q/Q	T&Q/T	T&Q/*p*	Term	Type	Description
!	6.91E-8	9273	175	85	48.6%	0.9%	20.93	GO:0043229	CC	intracellular organelle
!	7.04E-8	9276	175	85	48.6%	0.9%	20.91	GO:0043226	CC	organelle
!	2.21E-7	10946	175	93	53.1%	0.8%	19.86	GO:0044424	CC	intracellular part
!	1.33E-6	11457	175	94	53.7%	0.8%	18.07	GO:0005622	CC	intracellular
!	4.08E-6	2383	175	33	18.9%	1.4%	15.91	GO:0043228	CC	non-membrane-bounded organelle
!	4.08E-6	2383	175	33	18.9%	1.4%	15.91	GO:0043232	CC	intracellular non-membrane-bounded organelle
!	8.73E-6	7449	175	69	39.4%	0.9%	15.88	GO:0005737	CC	cytoplasm
!	1.61E-5	8208	175	73	41.7%	0.9%	15.33	GO:0043231	CC	intracellular membrane-bounded organelle
!	1.64E-5	8211	175	73	41.7%	0.9%	15.31	GO:0043227	CC	membrane-bounded organelle
	4.73E-5	8099	175	71	40.6%	0.9%	14.22	GO:0005515	MF	protein binding
!	4.35E-5	3681	175	41	23.4%	1.1%	13.76	GO:0019222	BP	regulation of metabolic process
!	9.75E-6	223	175	9	5.1%	4.0%	13.74	GO:0030036	BP	actin cytoskeleton organization
	7.07E-5	4993	175	50	28.6%	1.0%	13.47	GO:0034960	BP	cellular biopolymer metabolic process
!	1.64E-5	238	175	9	5.1%	3.8%	13.22	GO:0030029	BP	actin filament-based process
	8.37E-5	3520	175	39	22.3%	1.1%	13.05	GO:0031323	BP	regulation of cellular metabolic process
	1.29E-4	5392	175	52	29.7%	1.0%	12.91	GO:0005634	CC	nucleus
	1.39E-4	5118	175	50	28.6%	1.0%	12.79	GO:0044260	BP	cellular macromolecule metabolic process
	5.49E-5	496	175	12	6.9%	2.4%	12.29	GO:0051246	BP	regulation of protein metabolic process
	3.02E-4	5562	175	52	29.7%	0.9%	12.06	GO:0043283	BP	biopolymer metabolic process
	7.29E-4	13348	175	97	55.4%	0.7%	11.80	GO:0005488	MF	binding
	4.65E-5	105	175	6	3.4%	5.7%	11.77	GO:0006414	BP	translational elongation
	7.33E-4	12017	175	90	51.4%	0.7%	11.72	GO:0009987	BP	cellular process
	1.04E-4	450	175	11	6.3%	2.4%	11.57	GO:0006412	BP	translation
!	5.79E-5	88	175	6	3.4%	6.8%	11.55	REAC:192704	re	Synthesis of PB1-F2
	3.57E-4	3362	175	36	20.6%	1.1%	11.52	GO:0080090	BP	regulation of primary metabolic process
	2.99E-4	2564	175	30	17.1%	1.2%	11.52	GO:0044267	BP	cellular protein metabolic process
!	6.18E-5	89	175	6	3.4%	6.7%	11.48	REAC:72673	re	Release of 40S and 60S subunits from the 80S ribosome
!	6.18E-5	89	175	6	3.4%	6.7%	11.48	REAC:192823	re	Viral mRNA Translation
!	6.18E-5	89	175	6	3.4%	6.7%	11.48	REAC:192841	re	Viral Protein Synthesis
!	6.18E-5	89	175	6	3.4%	6.7%	11.48	REAC:156912	re	Peptide transfer from P-site tRNA to the A-site tRNA
	3.46E-4	2966	175	33	18.9%	1.1%	11.47	GO:0032502	BP	developmental process
!	6.58E-5	90	175	6	3.4%	6.7%	11.42	REAC:72672	re	The 60S subunit joins the translation initiation complex
!	6.58E-5	90	175	6	3.4%	6.7%	11.42	REAC:156907	re	Aminoacyl-tRNA binds to the ribosome at the A-site
!	6.58E-5	90	175	6	3.4%	6.7%	11.42	REAC:156923	re	Hydrolysis of eEF1A:GTP
!	6.58E-5	90	175	6	3.4%	6.7%	11.42	REAC:72671	re	eIF5B:GTP is hydrolyzed and released
	6.91E-4	6952	175	60	34.3%	0.9%	11.37	GO:0044238	BP	primary metabolic process

Sig.	*p*-value	T	Q	T&Q	T&Q/Q	T&Q/T	T&Q/*p*	Term	Type	Description
!	7.01E-5	91	175	6	3.4%	6.6%	11.36	REAC:156915	re	Translocation of ribosome by 3 bases in the 3' direction
!	7.01E-5	91	175	6	3.4%	6.6%	11.36	REAC:156902	re	Peptide chain elongation
!	7.01E-5	91	175	6	3.4%	6.6%	11.36	REAC:72764	re	Eukaryotic Translation Termination
!	7.01E-5	91	175	6	3.4%	6.6%	11.36	REAC:141673	re	GTP Hydrolysis by eRF3 bound to the eRF1:mRNA:polypeptide:80S Ribosome complex
!	7.01E-5	91	175	6	3.4%	6.6%	11.36	REAC:141671	re	Polypeptide release from the eRF3-GDP:eRF1:mRNA:80S Ribosome complex
!	7.01E-5	91	175	6	3.4%	6.6%	11.36	REAC:141691	re	GTP bound eRF3:eRF1 complex binds the peptidyl tRNA:mRNA:80S Ribosome complex
	6.18E-4	5715	175	52	29.7%	0.9%	11.34	GO:0043170	BP	macromolecule metabolic process
!	8.42E-5	94	175	6	3.4%	6.4%	11.17	REAC:156842	re	Eukaryotic Translation Elongation
	8.92E-5	118	175	6	3.4%	5.1%	11.12	GO:0006417	BP	regulation of translation
	8.83E-4	6702	175	58	33.1%	0.9%	11.09	GO:0044237	BP	cellular metabolic process
	2.46E-4	671	175	13	7.4%	1.9%	10.88	GO:0042981	BP	regulation of apoptosis
!	1.19E-4	100	175	6	3.4%	6.0%	10.83	REAC:72689	re	Formation of a pool of free 40S subunits
	2.80E-4	680	175	13	7.4%	1.9%	10.75	GO:0043067	BP	regulation of programmed cell death
	2.84E-4	681	175	13	7.4%	1.9%	10.73	GO:0010941	BP	regulation of cell death
	7.98E-4	3370	175	35	20.0%	1.0%	10.69	GO:0060255	BP	regulation of macromolecule metabolic process
	1.65E-4	153	175	7	4.0%	4.6%	10.66	REAC:168273	re	Influenza Viral RNA Transcription and Replication
	2.48E-4	416	175	10	5.7%	2.4%	10.60	GO:0007010	BP	cytoskeleton organization
	2.06E-4	260	175	8	4.6%	3.1%	10.57	GO:0003924	MF	GTPase activity
	1.94E-4	157	175	7	4.0%	4.5%	10.49	REAC:168255	re	Influenza Life Cycle
	3.17E-4	429	175	10	5.7%	2.3%	10.36	GO:0032268	BP	regulation of cellular protein metabolic process
	2.36E-4	162	175	7	4.0%	4.3%	10.30	REAC:168254	re	Influenza Infection
	2.13E-4	111	175	6	3.4%	5.4%	10.25	REAC:156827	re	L13a-mediated translational silencing of Ceruloplasmin expression
	2.13E-4	111	175	6	3.4%	5.4%	10.25	REAC:157279	re	3' -UTR-mediated translational regulation
	1.43E-4	40	175	4	2.3%	10.0%	10.24	TF:M00771_0	tf	ANNCACTTCCTG:0
	2.24E-4	112	175	6	3.4%	5.4%	10.20	REAC:72706	re	GTP hydrolysis and joining of the 60S ribosomal subunit
	1.02E-3	2375	175	27	15.4%	1.1%	10.18	GO:0016043	BP	cellular component organization
	3.12E-4	119	175	6	3.4%	5.0%	9.86	REAC:72737	re	Cap-dependent Translation Initiation
	3.12E-4	119	175	6	3.4%	5.0%	9.86	REAC:72613	re	Eukaryotic Translation Initiation
	1.12E-4	3	175	2	1.1%	66.7%	9.79	GO:0004045	MF	aminoacyl-tRNA hydrolase activity
	1.96E-3	3136	175	32	18.3%	1.0%	9.70	GO:0019538	BP	protein metabolic process
	1.63E-3	2325	175	26	14.9%	1.1%	9.68	GO:0034961	BP	cellular biopolymer biosynthetic process
	1.63E-3	2325	175	26	14.9%	1.1%	9.68	GO:0043284	BP	biopolymer biosynthetic process
	4.26E-4	126	175	6	3.4%	4.8%	9.55	REAC:72766	re	Translation
	2.47E-3	3047	175	31	17.7%	1.0%	9.44	GO:0031326	BP	regulation of cellular biosynthetic process
	2.60E-3	3057	175	31	17.7%	1.0%	9.39	GO:0009889	BP	regulation of biosynthetic process
	2.78E-4	21	175	3	1.7%	14.3%	9.29	GO:0008633	BP	activation of pro-apoptotic gene products

Sig.	*p*-value	T	Q	T&Q	T&Q/Q	T&Q/T	T&Q/*p*	Term	Type	Description
	3.25E-3	3646	175	35	20.0%	1.0%	9.28	GO:0003676	MF	nucleic acid binding
	1.50E-3	1023	175	15	8.6%	1.5%	9.21	GO:0044085	BP	cellular component biogenesis
	1.94E-3	1271	175	17	9.7%	1.3%	9.08	GO:0006996	BP	organelle organization
	9.19E-4	326	175	8	4.6%	2.5%	9.07	GO:0043065	BP	positive regulation of apoptosis
	1.40E-3	709	175	12	6.9%	1.7%	9.06	GO:0003723	MF	RNA binding
	1.75E-3	1039	175	15	8.6%	1.4%	9.06	GO:0005829	CC	cytosol
	2.99E-3	2123	175	25	14.3%	1.2%	9.03	TF:M00801_0	tf	CGTCAN:0
	9.75E-4	329	175	8	4.6%	2.4%	9.01	GO:0043068	BP	positive regulation of programmed cell death
!	6.18E-4	87	175	5	2.9%	5.7%	9.00	KEGG:03010	ke	Ribosome
	9.94E-4	330	175	8	4.6%	2.4%	8.99	GO:0010942	BP	positive regulation of cell death
	9.11E-4	202	175	7	4.0%	3.5%	8.95	REAC:392499	re	Metabolism of proteins
	8.16E-4	178	175	6	3.4%	3.4%	8.90	GO:0010608	BP	posttranscriptional regulation of gene expression
	3.81E-3	2732	175	28	16.0%	1.0%	8.90	GO:0007275	BP	multicellular organismal development
	5.01E-3	2521	175	26	14.9%	1.0%	8.55	GO:0034645	BP	cellular macromolecule biosynthetic process
	3.87E-4	4	175	2	1.1%	50.0%	8.55	REAC:139906	re	Activation of BAD by calcineurin
	7.37E-4	29	175	3	1.7%	10.3%	8.31	GO:0007569	BP	cell aging
	3.79E-3	1127	175	15	8.6%	1.3%	8.28	GO:0050793	BP	regulation of developmental process
	1.77E-3	281	175	7	4.0%	2.5%	8.28	GO:0005730	CC	nucleolus
	2.60E-3	630	175	10	5.7%	1.6%	8.25	MI:hsa-miR-15b*	mi	MI:hsa-miR-15b*
	8.15E-4	30	175	3	1.7%	10.0%	8.21	GO:0042990	BP	regulation of transcription factor import into nucleus
	1.82E-3	192	175	6	3.4%	3.1%	8.10	TF:M00136_2	tf	NNGAATATKCANNNN:2
	2.80E-3	391	175	9	5.1%	2.3%	8.08	REAC:74160	re	Gene Expression
	3.45E-3	766	175	11	6.3%	1.4%	8.07	MI:hsa-miR-483-5p	mi	MI:hsa-miR-483-5p
	6.42E-4	5	175	2	1.1%	40.0%	8.04	REAC:110029	re	RAF activation
	6.42E-4	5	175	2	1.1%	40.0%	8.04	REAC:109803	re	Transient dissociation of 14-3-3 upon Ras binding
	6.42E-4	5	175	2	1.1%	40.0%	8.04	REAC:109829	re	Stabilisation of RAF by further phosphorylation
	6.42E-4	5	175	2	1.1%	40.0%	8.04	REAC:109802	re	Stabilisation of RAS:RAF by 14-3-3
	1.30E-3	77	175	4	2.3%	5.2%	8.03	GO:0043523	BP	regulation of neuron apoptosis
	1.96E-3	195	175	6	3.4%	3.1%	8.03	TF:M00136_3	tf	NNGAATATKCANNNN:3
	4.44E-3	924	175	13	7.4%	1.4%	7.98	GO:0016462	MF	pyrophosphatase activity
	4.56E-3	927	175	13	7.4%	1.4%	7.96	GO:0016818	MF	hydrolase activity, acting on acid anhydrides, in phosphorus-containing anhydrides
	2.55E-3	300	175	7	4.0%	2.3%	7.92	GO:0005840	CC	ribosome
	7.72E-4	7	175	2	1.1%	28.6%	7.86	GO:0080010	BP	regulation of oxygen and reactive oxygen species metabolic process
	1.57E-3	81	175	4	2.3%	4.9%	7.84	GO:0022626	CC	cytosolic ribosome
	1.59E-3	75	175	4	2.3%	5.3%	7.83	TF:M00340_2	tf	KRCAGGAARTRNKT:2
	3.78E-3	501	175	9	5.1%	1.8%	7.78	GO:0008134	MF	transcription factor binding

Sig.	*p*-value	T	Q	T&Q	T&Q/Q	T&Q/T	T&Q/*p*	Term	Type	Description
	2.95E-3	308	175	7	4.0%	2.3%	7.77	GO:0043066	BP	negative regulation of apoptosis
	1.27E-3	27	175	3	1.7%	11.1%	7.77	REAC:109606	re	Intrinsic Pathway for Apoptosis
	3.83E-3	502	175	9	5.1%	1.8%	7.76	GO:0051093	BP	negative regulation of developmental process
	3.51E-3	404	175	8	4.6%	2.0%	7.73	GO:0005525	MF	GTP binding
	4.50E-3	681	175	10	5.7%	1.5%	7.71	MI:hsa-miR-568	mi	MI:hsa-miR-568
	4.14E-3	508	175	9	5.1%	1.8%	7.68	GO:0009628	BP	response to abiotic stimulus
	3.29E-3	314	175	7	4.0%	2.2%	7.66	GO:0060548	BP	negative regulation of cell death
	3.29E-3	314	175	7	4.0%	2.2%	7.66	GO:0043069	BP	negative regulation of programmed cell death
	9.58E-4	6	175	2	1.1%	33.3%	7.64	REAC:109841	re	RAF phosphorylates MEK1
	9.58E-4	6	175	2	1.1%	33.3%	7.64	REAC:109852	re	RAF phosphorylates MEK2
	2.42E-3	151	175	5	2.9%	3.3%	7.63	GO:0060537	BP	muscle tissue development
	2.93E-3	229	175	6	3.4%	2.6%	7.62	GO:0010627	BP	regulation of protein kinase cascade
	4.01E-3	413	175	8	4.6%	1.9%	7.60	GO:0019001	MF	guanyl nucleotide binding
	4.01E-3	413	175	8	4.6%	1.9%	7.60	GO:0032561	MF	guanyl ribonucleotide binding
	1.63E-3	38	175	3	1.7%	7.9%	7.52	GO:0042306	BP	regulation of protein import into nucleus
	1.63E-3	38	175	3	1.7%	7.9%	7.52	GO:0006413	BP	translational initiation
	3.10E-3	160	175	5	2.9%	3.1%	7.39	GO:0009314	BP	response to radiation
	1.90E-3	40	175	3	1.7%	7.5%	7.36	GO:0022627	CC	cytosolic small ribosomal subunit
	1.33E-3	7	175	2	1.1%	28.6%	7.32	REAC:110049	re	MEK activation
	1.33E-3	7	175	2	1.1%	28.6%	7.32	REAC:111447	re	Activation of BAD and translocation to mitochondria
	1.33E-3	7	175	2	1.1%	28.6%	7.32	REAC:112407	re	RAF phosphorylates MEK
	1.33E-3	7	175	2	1.1%	28.6%	7.32	REAC:109830	re	Activated RAF complex binds MEK
	4.68E-3	335	175	7	4.0%	2.1%	7.31	GO:0003712	MF	transcription cofactor activity
	3.59E-3	129	175	5	2.9%	3.9%	7.24	KEGG:04360	ke	Axon guidance
	2.50E-3	44	175	3	1.7%	6.8%	7.09	GO:0006446	BP	regulation of translational initiation
	2.50E-3	44	175	3	1.7%	6.8%	7.09	GO:0033157	BP	regulation of intracellular protein transport
	2.84E-3	46	175	3	1.7%	6.5%	6.96	GO:0043524	BP	negative regulation of neuron apoptosis
	4.85E-3	164	175	5	2.9%	3.0%	6.94	TF:M00340_3	tf	KRCAGGAARTRNKT:3
	4.16E-3	106	175	4	2.3%	3.8%	6.87	GO:0007265	BP	Ras protein signal transduction
	2.26E-3	9	175	2	1.1%	22.2%	6.79	REAC:109869	re	MAP kinase cascade
	3.40E-3	49	175	3	1.7%	6.1%	6.78	GO:0046822	BP	regulation of nucleocytoplasmic transport
	4.02E-3	52	175	3	1.7%	5.8%	6.62	GO:0022415	BP	viral reproductive process
	2.80E-3	13	175	2	1.1%	15.4%	6.57	GO:0042992	BP	negative regulation of transcription factor import into nucleus
	3.74E-3	15	175	2	1.1%	13.3%	6.28	GO:0042308	BP	negative regulation of protein import into nucleus
	4.09E-3	12	175	2	1.1%	16.7%	6.19	REAC:112410	re	SHC-mediated signalling
	4.74E-3	13	175	2	1.1%	15.4%	6.04	KEGG:03450	ke	Non-homologous end-joining

Sig.	*p*-value	T	Q	T&Q	T&Q/Q	T&Q/T	T&Q/*p*	Term	Type	Description
	4.80E-3	17	175	2	1.1%	11.8%	6.03	GO:0046823	BP	negative regulation of nucleocytoplasmic transport
	4.81E-3	13	175	2	1.1%	15.4%	6.03	REAC:179812	re	Grb2 events in EGFR signaling
	4.81E-3	13	175	2	1.1%	15.4%	6.03	REAC:112412	re	SOS-mediated signalling
	4.98E-3	16	175	2	1.1%	12.5%	6.00	TF:M00999_3	tf	WTNNNWNNTGGWWNNNWNGGNNWNWN:3

Sig.	*p*-value	T	Q	T&Q	T&Q/Q	T&Q/T	T&Q/*p*	Term	Type	Description
!	7.09E-7	7311	169	94	55.6%	1.3%	18.70	GO:0005737	CC	cytoplasm
!	4.23E-7	304	169	14	8.3%	4.6%	17.31	GO:0005840	CC	ribosome
!	2.72E-7	87	169	9	5.3%	10.3%	17.31	REAC:192704	re	Synthesis of PB1-F2
!	3.01E-7	88	169	9	5.3%	10.2%	17.21	REAC:192823	re	Viral mRNA Translation
!	3.01E-7	88	169	9	5.3%	10.2%	17.21	REAC:156912	re	Peptide transfer from P-site tRNA to the A-site tRNA
!	3.01E-7	88	169	9	5.3%	10.2%	17.21	REAC:72673	re	Release of 40S and 60S subunits from the 80S ribosome
!	3.01E-7	88	169	9	5.3%	10.2%	17.21	REAC:192841	re	Viral Protein Synthesis
!	3.69E-7	151	169	11	6.5%	7.3%	17.21	REAC:168273	re	Influenza Viral RNA Transcription and Replication
!	3.32E-7	89	169	9	5.3%	10.1%	17.12	REAC:72672	re	The 60S subunit joins the translation initiation complex
!	3.32E-7	89	169	9	5.3%	10.1%	17.12	REAC:156923	re	Hydrolysis of eEF1A:GTP
!	3.32E-7	89	169	9	5.3%	10.1%	17.12	REAC:156907	re	Aminoacyl-tRNA binds to the ribosome at the A-site
!	3.32E-7	89	169	9	5.3%	10.1%	17.12	REAC:72671	re	eIF5B:GTP is hydrolyzed and released
!	3.67E-7	90	169	9	5.3%	10.0%	17.02	REAC:156902	re	Peptide chain elongation
!	3.67E-7	90	169	9	5.3%	10.0%	17.02	REAC:72764	re	Eukaryotic Translation Termination
!	3.67E-7	90	169	9	5.3%	10.0%	17.02	REAC:141671	re	Polypeptide release from the eRF3-GDP:eRF1:mRNA:80S Ribosome complex
!	3.67E-7	90	169	9	5.3%	10.0%	17.02	REAC:156915	re	Translocation of ribosome by 3 bases in the 3' direction
!	3.67E-7	90	169	9	5.3%	10.0%	17.02	REAC:141691	re	GTP bound eRF3:eRF1 complex binds the peptidyl tRNA:mRNA:80S Ribosome complex
!	3.67E-7	90	169	9	5.3%	10.0%	17.02	REAC:141673	re	GTP Hydrolysis by eRF3 bound to the eRF1:mRNA:polypeptide:80S Ribosome complex
!	4.83E-7	155	169	11	6.5%	7.1%	16.94	REAC:168255	re	Influenza Life Cycle
!	4.88E-7	93	169	9	5.3%	9.7%	16.73	REAC:156842	re	Eukaryotic Translation Elongation
!	6.68E-7	160	169	11	6.5%	6.9%	16.62	REAC:168254	re	Influenza Infection
!	8.39E-7	99	169	9	5.3%	9.1%	16.19	REAC:72689	re	Formation of a pool of free 40S subunits
!	1.97E-6	130	169	9	5.3%	6.9%	15.33	GO:0033279	CC	ribosomal subunit
!	2.07E-6	110	169	9	5.3%	8.2%	15.29	REAC:156827	re	L13a-mediated translational silencing of Ceruloplasmin expression
!	2.07E-6	110	169	9	5.3%	8.2%	15.29	REAC:157279	re	3' -UTR-mediated translational regulation
!	2.24E-6	111	169	9	5.3%	8.1%	15.21	REAC:72706	re	GTP hydrolysis and joining of the 60S ribosomal subunit
!	3.34E-6	264	169	12	7.1%	4.5%	15.09	GO:0003735	MF	structural constituent of ribosome
!	2.92E-6	102	169	8	4.7%	7.8%	14.82	GO:0006414	BP	translational elongation
!	3.75E-6	118	169	9	5.3%	7.6%	14.69	REAC:72613	re	Eukaryotic Translation Initiation
!	3.75E-6	118	169	9	5.3%	7.6%	14.69	REAC:72737	re	Cap-dependent Translation Initiation
!	3.87E-6	88	169	8	4.7%	9.1%	14.54	KEGG:03010	ke	Ribosome
!	6.08E-6	125	169	9	5.3%	7.2%	14.21	REAC:72766	re	Translation
!	2.34E-5	1019	169	23	13.6%	2.3%	13.80	GO:0005829	CC	cytosol
!	2.14E-5	545	169	16	9.5%	2.9%	13.52	GO:0030529	CC	ribonucleoprotein complex
!	3.82E-5	450	169	14	8.3%	3.1%	12.81	GO:0006412	BP	translation
	1.13E-4	1443	169	27	16.0%	1.9%	12.38	GO:0048518	BP	positive regulation of biological process

Sig.	*p*-value	T	Q	T&Q	T&Q/Q	T&Q/T	T&Q/*p*	Term	Type	Description
	1.51E-4	1308	169	25	14.8%	1.9%	12.02	GO:0048522	BP	positive regulation of cellular process
	7.73E-4	10872	169	114	67.5%	1.0%	11.90	GO:0044424	CC	intracellular part
	6.85E-4	7897	169	89	52.7%	1.1%	11.77	GO:0005515	MF	protein binding
!	4.72E-5	75	169	6	3.6%	8.0%	11.75	GO:0022626	CC	cytosolic ribosome
	7.12E-5	158	169	8	4.7%	5.1%	11.63	GO:0044445	CC	cytosolic part
	2.37E-3	11368	169	116	68.6%	1.0%	10.80	GO:0005622	CC	intracellular
	7.07E-4	808	169	16	9.5%	2.0%	10.03	MI:mmu-miR-350	mi	MI:mmu-miR-350
	2.25E-4	51	169	5	3.0%	9.8%	10.01	REAC:156826	re	Dissociation of L13a from the 60s ribosomal subunit
	1.71E-3	2344	169	38	22.5%	1.6%	10.01	TF:M00104_3	tf	NATCGATCGS:3
	9.34E-4	911	169	18	10.7%	2.0%	9.87	GO:0005198	MF	structural molecule activity
	2.88E-3	4510	169	55	32.5%	1.2%	9.86	GO:0044444	CC	cytoplasmic part
	2.67E-4	66	169	5	3.0%	7.6%	9.84	GO:0015934	CC	large ribosomal subunit
	1.03E-3	837	169	16	9.5%	1.9%	9.65	MI:hsa-miR-513-5p	mi	MI:hsa-miR-513-5p
	1.15E-3	701	169	15	8.9%	2.1%	9.48	GO:0003723	MF	RNA binding
	9.21E-4	401	169	12	7.1%	3.0%	9.47	REAC:74160	re	Gene Expression
	3.62E-3	2952	169	44	26.0%	1.5%	9.41	TF:M00104_4	tf	NATCGATCGS:4
	3.93E-3	3055	169	40	23.7%	1.3%	9.23	GO:0044267	BP	cellular protein metabolic process
	4.48E-3	3078	169	40	23.7%	1.3%	9.10	GO:0019538	BP	protein metabolic process
	2.86E-3	757	169	14	8.3%	1.8%	8.50	MI:hsa-miR-521	mi	MI:hsa-miR-521
	8.47E-4	49	169	4	2.4%	8.2%	8.46	GO:0005761	CC	mitochondrial ribosome
	8.47E-4	49	169	4	2.4%	8.2%	8.46	GO:0000313	CC	organellar ribosome
	4.42E-4	4	169	2	1.2%	50.0%	8.42	GO:0010310	BP	regulation of hydrogen peroxide metabolic process
	4.42E-4	4	169	2	1.2%	50.0%	8.42	GO:0047238	MF	glucuronosyl-N-acetylgalactosaminyl-proteoglycan 4-beta-N-acetylgalactosaminyltransferase activity
	4.42E-4	4	169	2	1.2%	50.0%	8.42	GO:0050510	MF	N-acetylgalactosaminyl-proteoglycan 3-beta-glucuronosyltransferase activity
	1.32E-3	76	169	5	3.0%	6.6%	8.24	KEGG:04520	ke	Adherens junction
	3.77E-3	781	169	14	8.3%	1.8%	8.22	MI:hsa-miR-105	mi	MI:hsa-miR-105
	4.86E-3	1068	169	17	10.1%	1.6%	8.16	MI:mmu-miR-466a-3p	mi	MI:mmu-miR-466a-3p
	4.36E-3	794	169	14	8.3%	1.8%	8.07	MI:hsa-miR-549	mi	MI:hsa-miR-549
	7.32E-4	5	169	2	1.2%	40.0%	7.91	GO:0080010	BP	regulation of oxygen and reactive oxygen species metabolic process
	3.73E-3	421	169	10	5.9%	2.4%	7.89	GO:0045941	BP	positive regulation of transcription
	4.25E-3	429	169	10	5.9%	2.3%	7.76	GO:0010628	BP	positive regulation of gene expression
	1.91E-3	48	169	4	2.4%	8.3%	7.65	REAC:72676	re	eIF3 and eIF1A bind to the 40S subunit
	1.56E-3	21	169	3	1.8%	14.3%	7.56	REAC:182971	re	EGFR downregulation
	2.30E-3	64	169	4	2.4%	6.2%	7.46	GO:0030099	BP	myeloid cell differentiation
	1.78E-3	28	169	3	1.8%	10.7%	7.43	GO:0016605	CC	PML body

Sig.	*p*-value	T	Q	T&Q	T&Q/Q	T&Q/T	T&Q/*p*	Term	Type	Description
	2.39E-3	51	169	4	2.4%	7.8%	7.42	REAC:72695	re	Formation of the ternary complex, and subsequently, the 43S complex
	2.39E-3	51	169	4	2.4%	7.8%	7.42	REAC:72691	re	Formation of the 43S pre-initiation complex
	2.43E-3	65	169	4	2.4%	6.2%	7.41	GO:0015935	CC	small ribosomal subunit
	1.52E-3	7	169	2	1.2%	28.6%	7.18	GO:0060263	BP	regulation of respiratory burst
	1.84E-3	6	169	2	1.2%	33.3%	6.99	REAC:183051	re	Cbl ubiquitinates Sprouty
	1.84E-3	6	169	2	1.2%	33.3%	6.99	REAC:183089	re	Cbl binds and ubiquitinates Phospho-Sprouty
	1.84E-3	6	169	2	1.2%	33.3%	6.99	REAC:183084	re	Cbl escapes Cdc42-mediated inhibition by down-regulating the adaptor molecule betaPix
	3.83E-3	58	169	4	2.4%	6.9%	6.95	REAC:72621	re	Ribosomal scanning
	3.83E-3	58	169	4	2.4%	6.9%	6.95	REAC:157849	re	Formation of translation initiation complexes containing mRNA that does not circularize
	4.08E-3	59	169	4	2.4%	6.8%	6.89	REAC:156808	re	Formation of translation initiation complexes yielding circularized Ceruloplasmin mRNA in a 'closed-loop' conformation
	4.08E-3	59	169	4	2.4%	6.8%	6.89	REAC:72649	re	Translation initiation complex formation
	4.08E-3	59	169	4	2.4%	6.8%	6.89	REAC:72697	re	Start codon recognition
	4.08E-3	59	169	4	2.4%	6.8%	6.89	REAC:72619	re	eIF2:GTP is hydrolyzed, eIFs are released
	4.08E-3	59	169	4	2.4%	6.8%	6.89	REAC:72702	re	Ribosomal scanning and start codon recognition
	4.34E-3	60	169	4	2.4%	6.7%	6.83	REAC:72662	re	Activation of the mRNA upon binding of the cap-binding complex and eIFs, and subsequent binding to 43S
	4.34E-3	60	169	4	2.4%	6.7%	6.83	REAC:156823	re	Association of phospho-L13a with GAIT element of Ceruloplasmin mRNA
	3.40E-3	35	169	3	1.8%	8.6%	6.78	GO:0022627	CC	cytosolic small ribosomal subunit
	3.99E-3	37	169	3	1.8%	8.1%	6.62	GO:0022625	CC	cytosolic large ribosomal subunit
	4.63E-3	39	169	3	1.8%	7.7%	6.47	GO:0048660	BP	regulation of smooth muscle cell proliferation
	4.65E-3	12	169	2	1.2%	16.7%	6.06	GO:0018205	BP	peptidyl-lysine modification
	4.65E-3	12	169	2	1.2%	16.7%	6.06	GO:0048638	BP	regulation of developmental growth

Sig.	*p*-value	T	Q	T&Q	T&Q/Q	T&Q/T	T&Q/*p*	Term	Type	Description
!	1.97E-8	11368	209	161	77.0%	1.4%	22.82	GO:0005622	CC	intracellular
!	3.07E-7	10872	209	153	73.2%	1.4%	20.03	GO:0044424	CC	intracellular part
!	4.61E-6	7897	209	118	56.5%	1.5%	17.06	GO:0005515	MF	protein binding
!	2.35E-6	545	209	20	9.6%	3.7%	15.96	GO:0030529	CC	ribonucleoprotein complex
!	2.24E-5	9228	209	130	62.2%	1.4%	15.57	GO:0043229	CC	intracellular organelle
!	2.29E-5	9232	209	130	62.2%	1.4%	15.55	GO:0043226	CC	organelle
	6.85E-5	8203	209	117	56.0%	1.4%	14.35	GO:0043231	CC	intracellular membrane-bounded organelle
	6.98E-5	8206	209	117	56.0%	1.4%	14.33	GO:0043227	CC	membrane-bounded organelle
	7.00E-5	7311	209	107	51.2%	1.5%	14.24	GO:0005737	CC	cytoplasm
	1.64E-4	5463	209	84	40.2%	1.5%	13.15	GO:0034960	BP	cellular biopolymer metabolic process
	3.46E-4	12212	209	156	74.6%	1.3%	13.02	GO:0009987	BP	cellular process
	1.90E-4	5569	209	85	40.7%	1.5%	13.01	GO:0044260	BP	cellular macromolecule metabolic process
!	3.11E-5	304	209	13	6.2%	4.3%	12.94	GO:0005840	CC	ribosome
	2.09E-4	5499	209	84	40.2%	1.5%	12.9	GO:0043283	BP	biopolymer metabolic process
	2.67E-4	5621	209	85	40.7%	1.5%	12.67	GO:0043170	BP	macromolecule metabolic process
	2.41E-4	2584	209	47	22.5%	1.8%	12.18	GO:0010467	BP	gene expression
	4.48E-4	5450	209	82	39.2%	1.5%	12.12	GO:0005634	CC	nucleus
	3.39E-4	2772	209	49	23.4%	1.8%	11.88	GO:0032991	CC	macromolecular complex
	9.15E-5	130	209	8	3.8%	6.2%	11.38	GO:0033279	CC	ribosomal subunit
	1.73E-4	410	209	14	6.7%	3.4%	11.3	GO:0005525	MF	GTP binding
	2.36E-3	15188	209	181	86.6%	1.2%	11.25	GO:0008150	BP	biological_process
	1.45E-4	261	209	11	5.3%	4.2%	11.24	GO:0003924	MF	GTPase activity
	2.55E-4	701	209	19	9.1%	2.7%	11.22	GO:0003723	MF	RNA binding
	2.47E-3	13287	209	163	78.0%	1.2%	11.1	GO:0005488	MF	binding
	2.16E-4	419	209	14	6.7%	3.3%	11.08	GO:0019001	MF	guanyl nucleotide binding
	2.16E-4	419	209	14	6.7%	3.3%	11.08	GO:0032561	MF	guanyl ribonucleotide binding
	1.97E-3	7093	209	98	46.9%	1.4%	10.81	GO:0044237	BP	cellular metabolic process
	5.54E-4	1058	209	24	11.5%	2.3%	10.68	GO:0005739	CC	mitochondrion
	4.84E-4	564	209	16	7.7%	2.8%	10.41	GO:0044429	CC	mitochondrial part
	4.43E-4	450	209	14	6.7%	3.1%	10.36	GO:0006412	BP	translation
	4.00E-3	6883	209	94	45.0%	1.4%	10.06	GO:0044238	BP	primary metabolic process
	7.43E-4	709	209	17	8.1%	2.4%	10.04	MI:hsa-miR-488	mi	MI:hsa-miR-488
	2.36E-3	2332	209	40	19.1%	1.7%	9.74	GO:0043228	CC	non-membrane-bounded organelle
	2.36E-3	2332	209	40	19.1%	1.7%	9.74	GO:0043232	CC	intracellular non-membrane-bounded organelle
	2.90E-3	3055	209	49	23.4%	1.6%	9.73	GO:0044267	BP	cellular protein metabolic process
	1.19E-4	2	209	2	1.0%	100.0%	9.73	GO:0001812	BP	positive regulation of type I hypersensitivity

Sig.	*p*-value	T	Q	T&Q	T&Q/Q	T&Q/T	T&Q/*p*	Term	Type	Description
	1.19E-4	2	209	2	1.0%	100.0%	9.73	GO:0019767	MF	IgE receptor activity
	1.19E-4	2	209	2	1.0%	100.0%	9.73	GO:0001810	BP	regulation of type I hypersensitivity
	1.19E-4	2	209	2	1.0%	100.0%	9.73	GO:0033025	BP	regulation of mast cell apoptosis
	2.64E-4	29	209	4	1.9%	13.8%	9.63	GO:0042990	BP	regulation of transcription factor import into nucleus
	6.68E-4	264	209	10	4.8%	3.8%	9.61	GO:0003735	MF	structural constituent of ribosome
	3.38E-3	3078	209	49	23.4%	1.6%	9.58	GO:0019538	BP	protein metabolic process
	4.47E-3	4174	209	62	29.7%	1.5%	9.54	GO:0044446	CC	intracellular organelle part
	1.33E-3	748	209	17	8.1%	2.3%	9.46	MI:hsa-miR-411	mi	MI:hsa-miR-411
	4.97E-3	4193	209	62	29.7%	1.5%	9.43	GO:0044422	CC	organelle part
	5.65E-4	87	209	7	3.3%	8.0%	9.42	REAC:192704	re	Synthesis of PB1-F2
	6.06E-4	88	209	7	3.3%	8.0%	9.35	REAC:72673	re	Release of 40S and 60S subunits from the 80S ribosome
	6.06E-4	88	209	7	3.3%	8.0%	9.35	REAC:192841	re	Viral Protein Synthesis
	6.06E-4	88	209	7	3.3%	8.0%	9.35	REAC:192823	re	Viral mRNA Translation
	6.06E-4	88	209	7	3.3%	8.0%	9.35	REAC:156912	re	Peptide transfer from P-site tRNA to the A-site tRNA
	4.35E-3	3117	209	49	23.4%	1.6%	9.33	GO:0032502	BP	developmental process
	6.49E-4	89	209	7	3.3%	7.9%	9.29	REAC:72672	re	The 60S subunit joins the translation initiation complex
	6.49E-4	89	209	7	3.3%	7.9%	9.29	REAC:156923	re	Hydrolysis of eEF1A:GTP
	6.49E-4	89	209	7	3.3%	7.9%	9.29	REAC:156907	re	Aminoacyl-tRNA binds to the ribosome at the A-site
	6.49E-4	89	209	7	3.3%	7.9%	9.29	REAC:72671	re	eIF5B:GTP is hydrolyzed and released
	6.95E-4	90	209	7	3.3%	7.8%	9.22	REAC:72764	re	Eukaryotic Translation Termination
	6.95E-4	90	209	7	3.3%	7.8%	9.22	REAC:141691	re	GTP bound eRF3:eRF1 complex binds the peptidyl tRNA:mRNA:80S Ribosome complex
	6.95E-4	90	209	7	3.3%	7.8%	9.22	REAC:156902	re	Peptide chain elongation
	6.95E-4	90	209	7	3.3%	7.8%	9.22	REAC:141671	re	Polypeptide release from the eRF3-GDP:eRF1:mRNA:80S Ribosome complex
	6.95E-4	90	209	7	3.3%	7.8%	9.22	REAC:156915	re	Translocation of ribosome by 3 bases in the 3' direction
	6.95E-4	90	209	7	3.3%	7.8%	9.22	REAC:141673	re	GTP Hydrolysis by eRF3 bound to the eRF1:mRNA:polypeptide:80S Ribosome complex
	3.74E-3	1990	209	37	17.7%	1.9%	9.2	TF:M00137_3	tf	NNNRTAATNANNN:3
	1.24E-3	390	209	12	5.7%	3.1%	9.18	GO:0007010	BP	cytoskeleton organization
	3.38E-4	13	209	3	1.4%	23.1%	9.09	GO:0042992	BP	negative regulation of transcription factor import into nucleus
	8.47E-4	93	209	7	3.3%	7.5%	9.02	REAC:156842	re	Eukaryotic Translation Elongation
!	8.16E-4	88	209	6	2.9%	6.8%	8.9	KEGG:03010	ke	Ribosome
	1.12E-3	173	209	8	3.8%	4.6%	8.87	TF:M00136_2	tf	NNGAATATKCANNNN:2
	1.71E-3	405	209	12	5.7%	3.0%	8.86	GO:0005740	CC	mitochondrial envelope
	2.86E-4	2	209	2	1.0%	100.0%	8.85	REAC:139898	re	Caspase-8 activates BID by cleavage
	7.17E-4	65	209	5	2.4%	7.7%	8.85	GO:0015935	CC	small ribosomal subunit

Sig.	*p*-value	T	Q	T&Q	T&Q/Q	T&Q/T	T&Q/*p*	Term	Type	Description
	2.93E-3	928	209	20	9.6%	2.2%	8.83	GO:0016070	BP	RNA metabolic process
	8.88E-4	102	209	6	2.9%	5.9%	8.82	GO:0006414	BP	translational elongation
	2.14E-3	583	209	14	6.7%	2.4%	8.79	MI:hsa-miR-616*	mi	MI:hsa-miR-616*
	4.62E-3	1535	209	30	14.4%	2.0%	8.78	TF:M00187_4	tf	GYCACGTGNC:4
	1.25E-3	176	209	8	3.8%	4.5%	8.76	TF:M00136_3	tf	NNGAATATKCANNNN:3
	2.15E-3	434	209	13	6.2%	3.0%	8.71	TF:M00513_3	tf	CBCTGACGTCANCS:3
	6.87E-4	37	209	4	1.9%	10.8%	8.67	GO:0042306	BP	regulation of protein import into nucleus
	1.23E-3	99	209	7	3.3%	7.1%	8.65	REAC:72689	re	Formation of a pool of free 40S subunits
	3.53E-4	3	209	2	1.0%	66.7%	8.64	GO:0002866	BP	positive regulation of acute inflammatory response to antigenic stimulus
	3.53E-4	3	209	2	1.0%	66.7%	8.64	GO:0002885	BP	positive regulation of hypersensitivity
	3.53E-4	3	209	2	1.0%	66.7%	8.64	GO:0043306	BP	positive regulation of mast cell degranulation
	3.53E-4	3	209	2	1.0%	66.7%	8.64	GO:0019763	MF	immunoglobulin receptor activity
	3.53E-4	3	209	2	1.0%	66.7%	8.64	GO:0030346	MF	protein phosphatase 2B binding
	3.53E-4	3	209	2	1.0%	66.7%	8.64	GO:0043302	BP	positive regulation of leukocyte degranulation
	5.30E-4	15	209	3	1.4%	20.0%	8.64	GO:0042308	BP	negative regulation of protein import into nucleus
	4.45E-3	1308	209	25	12.0%	1.9%	8.63	GO:0048522	BP	positive regulation of cellular process
	1.88E-3	303	209	10	4.8%	3.3%	8.58	GO:0043065	BP	positive regulation of apoptosis
	3.25E-3	815	209	17	8.1%	2.1%	8.56	MI:mmu-miR-878-5p	mi	MI:mmu-miR-878-5p
	3.09E-3	742	209	16	7.7%	2.2%	8.55	MI:hsa-miR-620	mi	MI:hsa-miR-620
	2.02E-3	306	209	10	4.8%	3.3%	8.51	GO:0010942	BP	positive regulation of cell death
	2.02E-3	306	209	10	4.8%	3.3%	8.51	GO:0043068	BP	positive regulation of programmed cell death
	7.79E-4	17	209	3	1.4%	17.6%	8.26	GO:0046823	BP	negative regulation of nucleocytoplasmic transport
	1.37E-3	75	209	5	2.4%	6.7%	8.2	GO:0022626	CC	cytosolic ribosome
	2.41E-3	213	209	8	3.8%	3.8%	8.11	GO:0030036	BP	actin cytoskeleton organization
	1.22E-3	43	209	4	1.9%	9.3%	8.1	GO:0033157	BP	regulation of intracellular protein transport
	4.73E-3	706	209	15	7.2%	2.1%	8.06	MI:hsa-miR-127-3p	mi	MI:hsa-miR-127-3p
	3.19E-3	326	209	10	4.8%	3.1%	8.05	GO:0007417	BP	central nervous system development
	2.28E-3	110	209	7	3.3%	6.4%	8.03	REAC:157279	re	3' -UTR-mediated translational regulation
	2.28E-3	110	209	7	3.3%	6.4%	8.03	REAC:156827	re	L13a-mediated translational silencing of Ceruloplasmin expression
	2.40E-3	111	209	7	3.3%	6.3%	7.98	REAC:72706	re	GTP hydrolysis and joining of the 60S ribosomal subunit
	7.01E-4	4	209	2	1.0%	50.0%	7.96	GO:0022011	BP	myelination in the peripheral nervous system
	7.01E-4	4	209	2	1.0%	50.0%	7.96	GO:0002864	BP	regulation of acute inflammatory response to antigenic stimulus
	7.01E-4	4	209	2	1.0%	50.0%	7.96	GO:0043304	BP	regulation of mast cell degranulation
	7.01E-4	4	209	2	1.0%	50.0%	7.96	GO:0002883	BP	regulation of hypersensitivity
	7.01E-4	4	209	2	1.0%	50.0%	7.96	GO:0032292	BP	ensheathment of axons in the peripheral nervous system
	1.45E-3	45	209	4	1.9%	8.9%	7.92	GO:0017015	BP	regulation of transforming growth factor beta receptor signaling pathway

Sig.	*p*-value	T	Q	T&Q	T&Q/Q	T&Q/T	T&Q/*p*	Term	Type	Description
	4.62E-3	458	209	12	5.7%	2.6%	7.86	GO:0051094	BP	positive regulation of developmental process
	1.27E-3	20	209	3	1.4%	15.0%	7.77	GO:0007569	BP	cell aging
	8.50E-4	3	209	2	1.0%	66.7%	7.76	REAC:196108	re	Pregnenolone biosynthesis
	1.70E-3	47	209	4	1.9%	8.5%	7.76	GO:0046822	BP	regulation of nucleocytoplasmic transport
	3.49E-3	151	209	8	3.8%	5.3%	7.74	REAC:168273	re	Influenza Viral RNA Transcription and Replication
	3.75E-3	229	209	8	3.8%	3.5%	7.67	GO:0030029	BP	actin filament-based process
	3.41E-3	118	209	7	3.3%	5.9%	7.63	REAC:72613	re	Eukaryotic Translation Initiation
	3.41E-3	118	209	7	3.3%	5.9%	7.63	REAC:72737	re	Cap-dependent Translation Initiation
	1.47E-3	21	209	3	1.4%	14.3%	7.62	GO:0032387	BP	negative regulation of intracellular transport
	1.47E-3	21	209	3	1.4%	14.3%	7.62	GO:0008633	BP	activation of pro-apoptotic gene products
	4.11E-3	155	209	8	3.8%	5.2%	7.57	REAC:168255	re	Influenza Life Cycle
	3.30E-3	132	209	6	2.9%	4.5%	7.51	GO:0031667	BP	response to nutrient levels
	1.16E-3	5	209	2	1.0%	40.0%	7.45	GO:0043300	BP	regulation of leukocyte degranulation
	1.16E-3	5	209	2	1.0%	40.0%	7.45	GO:0002863	BP	positive regulation of inflammatory response to antigenic stimulus
	1.16E-3	5	209	2	1.0%	40.0%	7.45	GO:0033005	BP	positive regulation of mast cell activation
	1.16E-3	5	209	2	1.0%	40.0%	7.45	GO:0014044	BP	Schwann cell development
	1.16E-3	5	209	2	1.0%	40.0%	7.45	GO:0019863	MF	IgE binding
	4.99E-3	160	209	8	3.8%	5.0%	7.38	REAC:168254	re	Influenza Infection
	4.66E-3	188	209	7	3.3%	3.7%	7.31	GO:0007517	BP	muscle development
	4.71E-3	125	209	7	3.3%	5.6%	7.3	REAC:72766	re	Translation
	4.10E-3	138	209	6	2.9%	4.3%	7.29	GO:0009991	BP	response to extracellular stimulus
	4.10E-3	138	209	6	2.9%	4.3%	7.29	GO:0001817	BP	regulation of cytokine production
	4.25E-3	139	209	6	2.9%	4.3%	7.25	GO:0060341	BP	regulation of cellular localization
	2.19E-3	24	209	3	1.4%	12.5%	7.22	GO:0030532	CC	small nuclear ribonucleoprotein complex
	1.68E-3	4	209	2	1.0%	50.0%	7.08	REAC:202724	re	P-selectin binds P-selectin ligand
	1.68E-3	4	209	2	1.0%	50.0%	7.08	REAC:75108	re	Activation, myristolyation of BID and translocation to mitochondria
	1.73E-3	6	209	2	1.0%	33.3%	7.05	GO:0033032	BP	regulation of myeloid cell apoptosis
	1.73E-3	6	209	2	1.0%	33.3%	7.05	GO:0045921	BP	positive regulation of exocytosis
	1.73E-3	6	209	2	1.0%	33.3%	7.05	GO:0033003	BP	regulation of mast cell activation
	1.73E-3	6	209	2	1.0%	33.3%	7.05	GO:0002886	BP	regulation of myeloid leukocyte mediated immunity
	3.69E-3	58	209	4	1.9%	6.9%	6.99	GO:0032386	BP	regulation of intracellular transport
	3.93E-3	59	209	4	1.9%	6.8%	6.93	GO:0043523	BP	regulation of neuron apoptosis
	3.43E-3	28	209	3	1.4%	10.7%	6.77	GO:0006749	BP	glutathione metabolic process
	3.43E-3	28	209	3	1.4%	10.7%	6.77	GO:0043524	BP	negative regulation of neuron apoptosis
	2.40E-3	7	209	2	1.0%	28.6%	6.73	GO:0014037	BP	Schwann cell differentiation
	2.40E-3	7	209	2	1.0%	28.6%	6.73	GO:0002891	BP	positive regulation of immunoglobulin mediated immune response

Sig.	*p*-value	T	Q	T&Q	T&Q/Q	T&Q/T	T&Q/*p*	Term	Type	Description
	2.40E-3	7	209	2	1.0%	28.6%	6.73	GO:0002714	BP	positive regulation of B cell mediated immunity
	4.19E-3	30	209	3	1.4%	10.0%	6.57	GO:0042542	BP	response to hydrogen peroxide
	4.60E-3	31	209	3	1.4%	9.7%	6.48	GO:0006879	BP	cellular iron ion homeostasis
	3.18E-3	8	209	2	1.0%	25.0%	6.44	GO:0002861	BP	regulation of inflammatory response to antigenic stimulus
	3.18E-3	8	209	2	1.0%	25.0%	6.44	GO:0002675	BP	positive regulation of acute inflammatory response
	4.11E-3	6	209	2	1.0%	33.3%	6.19	REAC:171026	re	Guanine nucleotide exchange on Ral
	5.04E-3	10	209	2	1.0%	20.0%	5.98	GO:0042994	BP	cytoplasmic sequestering of transcription factor

Sig.	*p*-value	T	Q	T&Q	T&Q/Q	T&Q/T	T&Q/*p*	Term	Type	Description
	1.65E-4	642	78	11	14.1%	1.7%	11.11	TF:M00981_4	tf	NTGACGTNA:4
	1.52E-4	529	78	10	12.8%	1.9%	11.09	TF:M00981_2	tf	NTGACGTNA:2
	2.32E-4	668	78	11	14.1%	1.6%	10.77	TF:M00917_3	tf	CNNTGACGTMA:3
	2.66E-4	567	78	10	12.8%	1.8%	10.53	TF:M00039_0	tf	TGACGTMA:0
	2.70E-4	568	78	10	12.8%	1.8%	10.52	TF:M00514_4	tf	CVTGACGYMABG:4
	3.41E-4	818	78	12	15.4%	1.5%	10.47	TF:M00916_3	tf	NNTKACGTCANNNS:3
	2.59E-4	362	78	8	10.3%	2.2%	10.34	TF:M00178_4	tf	NSTGACGTMANN:4
	4.01E-4	712	78	11	14.1%	1.5%	10.22	TF:M00041_0	tf	TGACGTYA:0
	4.57E-4	497	78	9	11.5%	1.8%	9.89	TF:M00513_4	tf	CBCTGACGTCANCS:4
	7.06E-4	1197	78	13	16.7%	1.1%	9.82	GO:0006996	BP	organelle organization
	7.97E-4	773	78	11	14.1%	1.4%	9.53	TF:M00917_4	tf	CNNTGACGTMA:4
	1.20E-3	943	78	12	15.4%	1.3%	9.21	TF:M00916_4	tf	NNTKACGTCANNNS:4
	8.61E-4	434	78	8	10.3%	1.8%	9.14	TF:M00513_3	tf	CBCTGACGTCANCS:3
	1.36E-3	466	78	8	10.3%	1.7%	8.68	TF:M00917_2	tf	CNNTGACGTMA:2
	4.61E-3	2211	78	17	21.8%	0.8%	8.21	GO:0016043	BP	cellular component organization
	1.99E-3	385	78	7	9.0%	1.8%	8.17	TF:M00981_0	tf	NTGACGTNA:0
	2.76E-3	809	78	9	11.5%	1.1%	8.09	MI:hsa-miR-383	mi	MI:hsa-miR-383
	6.29E-4	7	78	2	2.6%	28.6%	8.06	REAC:111453	re	BH3-only proteins associate with and inactivate anti-apoptotic BCL-2 members
	2.97E-3	818	78	9	11.5%	1.1%	8.02	MI:hsa-miR-486-3p	mi	MI:hsa-miR-486-3p
	3.44E-3	836	78	9	11.5%	1.1%	7.87	MI:mmu-miR-883b-3p	mi	MI:mmu-miR-883b-3p
	3.90E-3	1017	78	10	12.8%	1.0%	7.85	MI:hsa-miR-519b-3p	mi	MI:hsa-miR-519b-3p
	3.26E-3	675	78	8	10.3%	1.2%	7.81	MI:hsa-miR-933	mi	MI:hsa-miR-933
	2.50E-3	297	78	6	7.7%	2.0%	7.78	TF:M00178_3	tf	NSTGACGTMANN:3
	4.07E-3	438	78	7	9.0%	1.6%	7.45	TF:M00514_3	tf	CVTGACGYMABG:3
	4.82E-3	532	78	7	9.0%	1.3%	7.28	GO:0034621	BP	cellular macromolecular complex subunit organization
	3.92E-3	139	78	4	5.1%	2.9%	6.93	TF:M00514_2	tf	CVTGACGYMABG:2
	4.40E-3	169	78	4	5.1%	2.4%	6.81	GO:0004721	MF	phosphoprotein phosphatase activity
	2.66E-3	14	78	2	2.6%	14.3%	6.62	REAC:114452	re	Activation of BH3-only proteins
	3.04E-3	21	78	2	2.6%	9.5%	6.49	GO:0008633	BP	activation of pro-apoptotic gene products
	3.93E-3	1	78	1	1.3%	100.0%	5.54	GO:0015734	BP	taurine transport
	3.93E-3	1	78	1	1.3%	100.0%	5.54	GO:0001762	BP	beta-alanine transport
	3.93E-3	1	78	1	1.3%	100.0%	5.54	GO:0005369	MF	taurine:sodium symporter activity
	3.93E-3	1	78	1	1.3%	100.0%	5.54	GO:0042412	BP	taurine biosynthetic process
	3.93E-3	1	78	1	1.3%	100.0%	5.54	GO:0017172	MF	cysteine dioxygenase activity
	3.93E-3	1	78	1	1.3%	100.0%	5.54	GO:0046439	BP	L-cysteine metabolic process
	3.93E-3	1	78	1	1.3%	100.0%	5.54	GO:0001761	MF	beta-alanine transmembrane transporter activity

Sig.	*p*-value	T	Q	T&Q	T&Q/Q	T&Q/T	T&Q/*p*	Term	Type	Description
	3.93E-3	1	78	1	1.3%	100.0%	5.54	GO:0005368	MF	taurine transmembrane transporter activity
	3.93E-3	1	78	1	1.3%	100.0%	5.54	GO:0035148	BP	lumen formation
	3.93E-3	1	78	1	1.3%	100.0%	5.54	GO:0030047	BP	actin modification
	4.63E-3	1	78	1	1.3%	100.0%	5.38	TF:M00235_0	tf	KNNKNNTYGCGTGCMS:0

Sig.	*p*-value	T	Q	T&Q	T&Q/Q	T&Q/T	T&Q/*p*	Term	Type	Description
	3.86E-4	11	47	2	4.3%	18.2%	8.55	REAC:140875	re	Common Pathway
	1.45E-3	293	47	5	10.6%	1.7%	8.15	TF:M00216_4	tf	NCTATAAAAR:4
	2.10E-3	597	47	6	12.8%	1.0%	7.96	MI:hsa-miR-661	mi	MI:hsa-miR-661
	2.78E-3	29	47	2	4.3%	6.9%	6.58	REAC:140877	re	Formation of Fibrin Clot (Clotting Cascade)
	4.03E-3	40	47	2	4.3%	5.0%	6.21	GO:0004623	MF	phospholipase A2 activity
	2.37E-3	1	47	1	2.1%	100.0%	6.04	GO:0051882	BP	mitochondrial depolarization
	2.37E-3	1	47	1	2.1%	100.0%	6.04	GO:0005760	CC	gamma DNA polymerase complex
	2.37E-3	1	47	1	2.1%	100.0%	6.04	GO:0019402	BP	galactitol metabolic process
	2.37E-3	1	47	1	2.1%	100.0%	6.04	GO:0043265	CC	ectoplasm
	2.37E-3	1	47	1	2.1%	100.0%	6.04	GO:0015670	BP	carbon dioxide transport
	2.85E-3	1	47	1	2.1%	100.0%	5.86	REAC:70355	re	ATP + D-galactose => ADP + D-galactose 1-phosphate
	2.85E-3	1	47	1	2.1%	100.0%	5.86	REAC:140806	re	antithrombin III + heparin -> antithrombin III:heparin
	4.73E-3	2	47	1	2.1%	50.0%	5.35	GO:0006264	BP	mitochondrial DNA replication
	4.73E-3	2	47	1	2.1%	50.0%	5.35	GO:0004335	MF	galactokinase activity
	4.73E-3	2	47	1	2.1%	50.0%	5.35	GO:0019031	CC	viral envelope
	4.73E-3	2	47	1	2.1%	50.0%	5.35	GO:0030337	MF	DNA polymerase processivity factor activity
	4.73E-3	2	47	1	2.1%	50.0%	5.35	GO:0007100	BP	mitotic centrosome separation
	4.73E-3	2	47	1	2.1%	50.0%	5.35	GO:0008969	MF	phosphohistidine phosphatase activity
	4.73E-3	2	47	1	2.1%	50.0%	5.35	GO:0006059	BP	hexitol metabolic process
	4.73E-3	2	47	1	2.1%	50.0%	5.35	GO:0051300	BP	spindle pole body organization
	4.73E-3	2	47	1	2.1%	50.0%	5.35	GO:0019400	BP	alditol metabolic process
	4.73E-3	2	47	1	2.1%	50.0%	5.35	GO:0043626	CC	PCNA complex
	4.73E-3	2	47	1	2.1%	50.0%	5.35	GO:0004373	MF	glycogen (starch) synthase activity
	4.73E-3	2	47	1	2.1%	50.0%	5.35	GO:0032042	BP	mitochondrial DNA metabolic process

Sig.	*p*-value	T	Q	T&Q	T&Q/Q	T&Q/T	T&Q/*p*	Term	Type	Description
	1.22E-3	3350	66	23	34.8%	0.7%	9.84	TF:M00122_4	tf	NNRNCACGTGNYNN:4
	1.52E-3	2967	66	21	31.8%	0.7%	9.53	TF:M00965_3	tf	YGAMCTNNASTRACCYN:3
	8.93E-4	988	66	11	16.7%	1.1%	9.42	TF:M00122_1	tf	NNRNCACGTGNYNN:1
	1.63E-3	17	66	2	3.0%	11.8%	7.11	GO:0030149	BP	sphingolipid catabolic process
	3.10E-3	82	66	3	4.5%	3.7%	6.87	GO:0044242	BP	cellular lipid catabolic process
	2.49E-3	21	66	2	3.0%	9.5%	6.69	GO:0006776	BP	vitamin A metabolic process
	3.78E-3	88	66	3	4.5%	3.4%	6.68	GO:0050660	MF	FAD binding
	4.68E-3	95	66	3	4.5%	3.2%	6.46	GO:0016627	MF	oxidoreductase activity, acting on the CH-CH group of donors
	4.11E-3	27	66	2	3.0%	7.4%	6.19	GO:0006775	BP	fat-soluble vitamin metabolic process
	4.74E-3	29	66	2	3.0%	6.9%	6.04	GO:0046466	BP	membrane lipid catabolic process
	2.85E-3	1	66	1	1.5%	100.0%	5.86	REAC:211930	re	CYP26B1 also deactivates all-trans-retinoic acid by 4-hydroxylation
	2.85E-3	1	66	1	1.5%	100.0%	5.86	REAC:192036	re	5Beta-cholesten-7alpha, 12alpha-diol-3-one is reduced to 5beta-cholestan-3alpha, 7alpha, 12alpha-triol
	2.85E-3	1	66	1	1.5%	100.0%	5.86	REAC:193800	re	5Beta-cholestan-7alpha,12alpha,27-triol-3-one is reduced to 5beta-cholestan-3alpha,7alpha,12alpha,27-tetrol
	2.85E-3	1	66	1	1.5%	100.0%	5.86	REAC:70745	re	isovaleryl-CoA + FAD => beta-methylcrotonyl-CoA + FADH2
	2.85E-3	1	66	1	1.5%	100.0%	5.86	REAC:193841	re	5beta-cholestan-7alpha,27-diol-3-one is reduced to 5beta-cholestan-3alpha,7alpha,27-triol
	2.85E-3	1	66	1	1.5%	100.0%	5.86	REAC:193758	re	5beta-cholestan-7alpha,24(S)-diol-3-one is reduced to 5beta-cholestan-3alpha,7alpha,24(S)-triol
	2.85E-3	1	66	1	1.5%	100.0%	5.86	REAC:202692	re	PIP3 binds to RhoA and activates it
	2.85E-3	1	66	1	1.5%	100.0%	5.86	REAC:193781	re	5Beta-cholestan-7alpha,12alpha,24(S)-triol-3-one is reduced to 5beta-cholestan-3alpha,7alpha,12alpha,24(S)-tetrol
	2.85E-3	1	66	1	1.5%	100.0%	5.86	REAC:192160	re	5beta-cholestan-7alpha-ol-3-one is reduced to 5beta-cholestan-3alpha, 7alpha-diol
	3.55E-3	1	66	1	1.5%	100.0%	5.64	GO:0008470	MF	isovaleryl-CoA dehydrogenase activity
	3.55E-3	1	66	1	1.5%	100.0%	5.64	GO:0051786	MF	all-trans-retinol 13,14-reductase activity
	3.55E-3	1	66	1	1.5%	100.0%	5.64	GO:0050290	MF	sphingomyelin phosphodiesterase D activity
	3.55E-3	1	66	1	1.5%	100.0%	5.64	GO:0002418	BP	immune response to tumor cell
	3.55E-3	1	66	1	1.5%	100.0%	5.64	GO:0008117	MF	sphinganine-1-phosphate aldolase activity
	3.55E-3	1	66	1	1.5%	100.0%	5.64	GO:0047743	MF	chlordecone reductase activity

Sig.	*p*-value	T	Q	T&Q	T&Q/Q	T&Q/T	T&Q/*p*	Term	Type	Description
	8.03E-4	818	41	7	17.1%	0.9%	9.07	MI:hsa-let-7b	mi	MI:hsa-let-7b
	8.53E-4	164	41	4	9.8%	2.4%	8.45	TF:M00460_4	tf	TTCCNRGAANNNNNNNTTCCNNGRR:4
	3.30E-3	1963	41	12	29.3%	0.6%	8.20	TF:M00632_2	tf	AGATADMAGGGA:2
	6.10E-4	19	41	2	4.9%	10.5%	8.10	GO:0015026	MF	coreceptor activity
	2.32E-3	983	41	7	17.1%	0.7%	8.01	MI:hsa-miR-518d-3p	mi	MI:hsa-miR-518d-3p
	2.13E-3	353	41	5	12.2%	1.4%	7.76	TF:M00281_4	tf	NNGTNRCNATRGYAACNN:4
	2.90E-3	757	41	6	14.6%	0.8%	7.63	MI:mmu-miR-468	mi	MI:mmu-miR-468
	3.88E-3	803	41	6	14.6%	0.7%	7.34	MI:hsa-let-7c	mi	MI:hsa-let-7c
	2.95E-3	230	41	4	9.8%	1.7%	7.21	TF:M00341_3	tf	VCCGGAAGNGCR:3
	4.63E-3	261	41	4	9.8%	1.5%	6.76	TF:M00281_3	tf	NNGTNRCNATRGYAACNN:3
	3.14E-3	53	41	2	4.9%	3.8%	6.46	KEGG:05130	ke	Pathogenic Escherichia coli infection - EHEC
	3.14E-3	53	41	2	4.9%	3.8%	6.46	KEGG:05131	ke	Pathogenic Escherichia coli infection - EPEC
	1.94E-3	1	41	1	2.4%	100.0%	6.25	GO:0015215	MF	nucleotide transmembrane transporter activity
	1.94E-3	1	41	1	2.4%	100.0%	6.25	GO:0045065	BP	cytotoxic T cell differentiation
	1.94E-3	1	41	1	2.4%	100.0%	6.25	GO:0048210	BP	Golgi vesicle fusion to target membrane
	1.94E-3	1	41	1	2.4%	100.0%	6.25	GO:0030302	BP	deoxynucleotide transport
	1.94E-3	1	41	1	2.4%	100.0%	6.25	GO:0015670	BP	carbon dioxide transport
	1.94E-3	1	41	1	2.4%	100.0%	6.25	GO:0030233	MF	deoxynucleotide transmembrane transporter activity
	4.58E-3	33	41	2	4.9%	6.1%	6.08	REAC:198203	re	PI3K/AKT signalling
	4.62E-3	39	41	2	4.9%	5.1%	6.07	TF:M00460_2	tf	TTCCNRGAANNNNNNNTTCCNNGRR:2
	2.61E-3	1	41	1	2.4%	100.0%	5.95	TF:M00235_0	tf	KNNKNNTYGCGTGCMS:0
	3.21E-3	1	41	1	2.4%	100.0%	5.74	REAC:76518	re	Dehydrogenation of Sparteine to form 2-Dehydrosparteine
	3.21E-3	1	41	1	2.4%	100.0%	5.74	REAC:202692	re	PIP3 binds to RhoA and activates it
	3.21E-3	1	41	1	2.4%	100.0%	5.74	REAC:211966	re	CYP2D6 4-hydroxylates debrisoquine
	3.21E-3	1	41	1	2.4%	100.0%	5.74	REAC:140806	re	antithrombin III + heparin -> antithrombin III:heparin
	3.87E-3	2	41	1	2.4%	50.0%	5.55	GO:0006862	BP	nucleotide transport

Key

Sig indicates significance (!) under multiple hypothesis testing. ***p*-value** all terms with *p*-value < 0.005 are listed. **T** is the total number of genes over the human genome that are annotated by this term. **Q** is the total number of recognised genes in the signature. **T&Q** is the total number of genes in the signature that are annotated by this term. **T&Q/Q** is the proportion of genes in the signature that are annotated by this term. **T&Q/T** is the proportion of genes in T that occur in this signature. **Term** is the term's identifier; clicking on the link will open a webpage reference for the term. **Type** – Gene Ontology (CC: cellular components; MF: molecular function; BP: biological process), Reactome pathway (re), KEGG pathway (ke), transcription factor (tf), and microRNA (mi). **Description** is a brief description of the term.
