# Supplementary material for: A Transcription Factor Map as Revealed by a Genome-Wide Gene Expression Analysis of Whole-Blood mRNA Transcriptome in Multiple Sclerosis
Source: PLoS One. 2010 Dec 1;5(12):e14176. doi: 10.1371/journal.pone.0014176 (PMC2995726; doi:10.1371/journal.pone.0014176)
Supplement: Figure S1 — Stars correlation plots. Correlation plots for all high cardinality stars. (8.18 MB PDF) [file pone.0014176.s008.pdf]

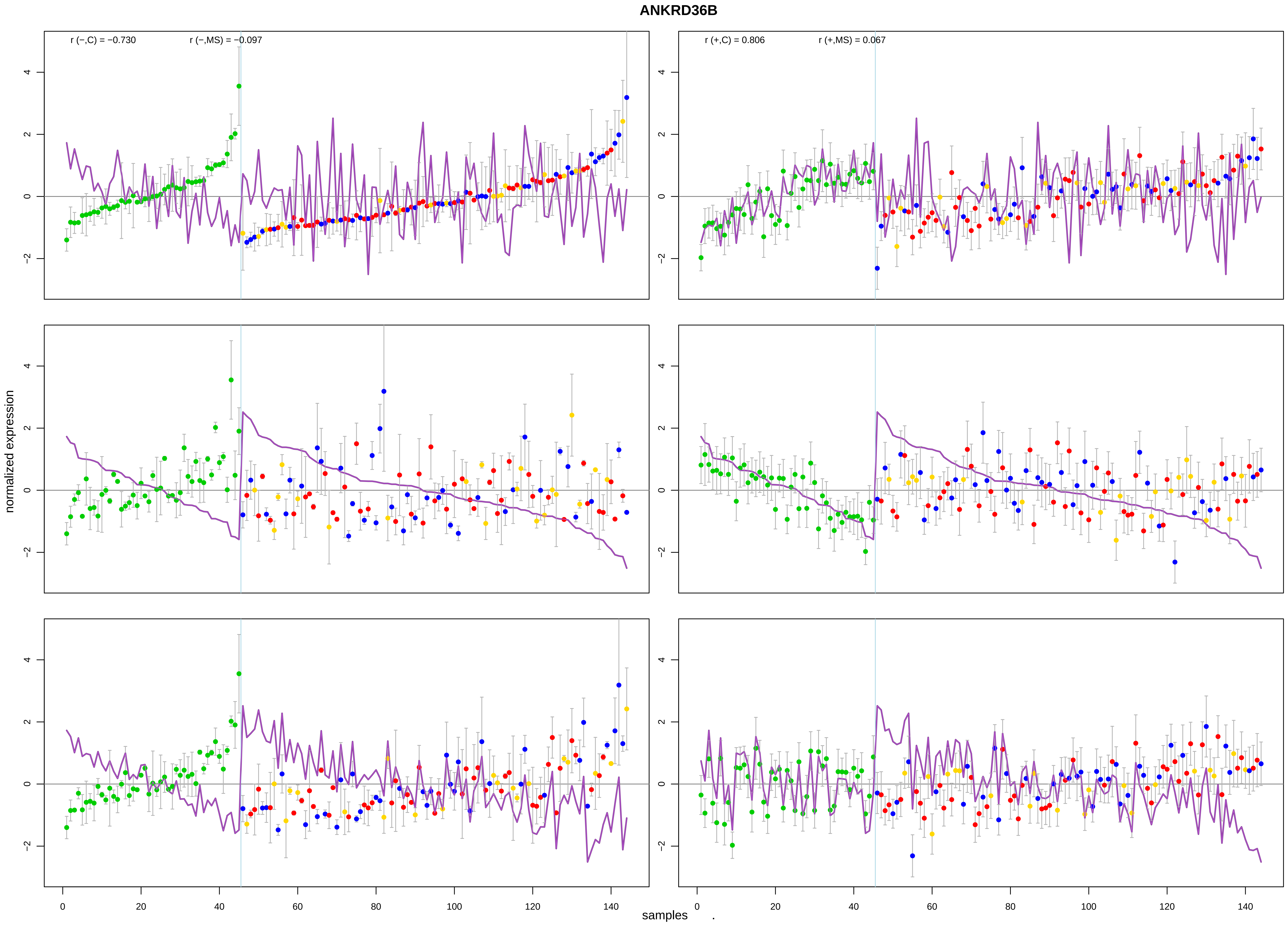

ANKRD36B
r (−,C) = −0.730
r (−,MS) = −0.097
r (+,C) = 0.806
r (+,MS) = 0.067
normalized expression
samples

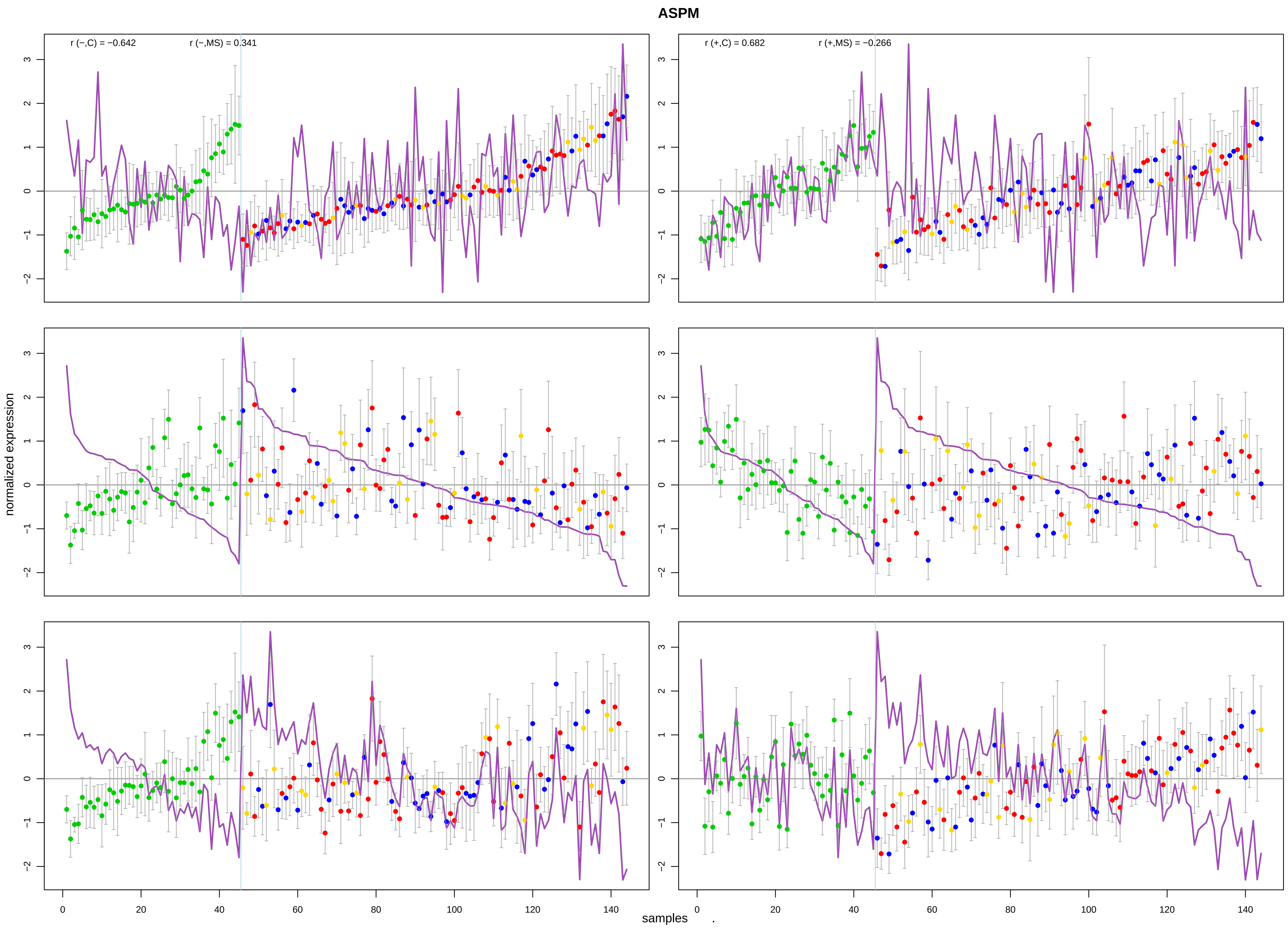
ASPM
r (−,C) = −0.642
r (−,MS) = 0.341
r (+,C) = 0.682
r (+,MS) = −0.266
normalized expression
samples

C20ORF134

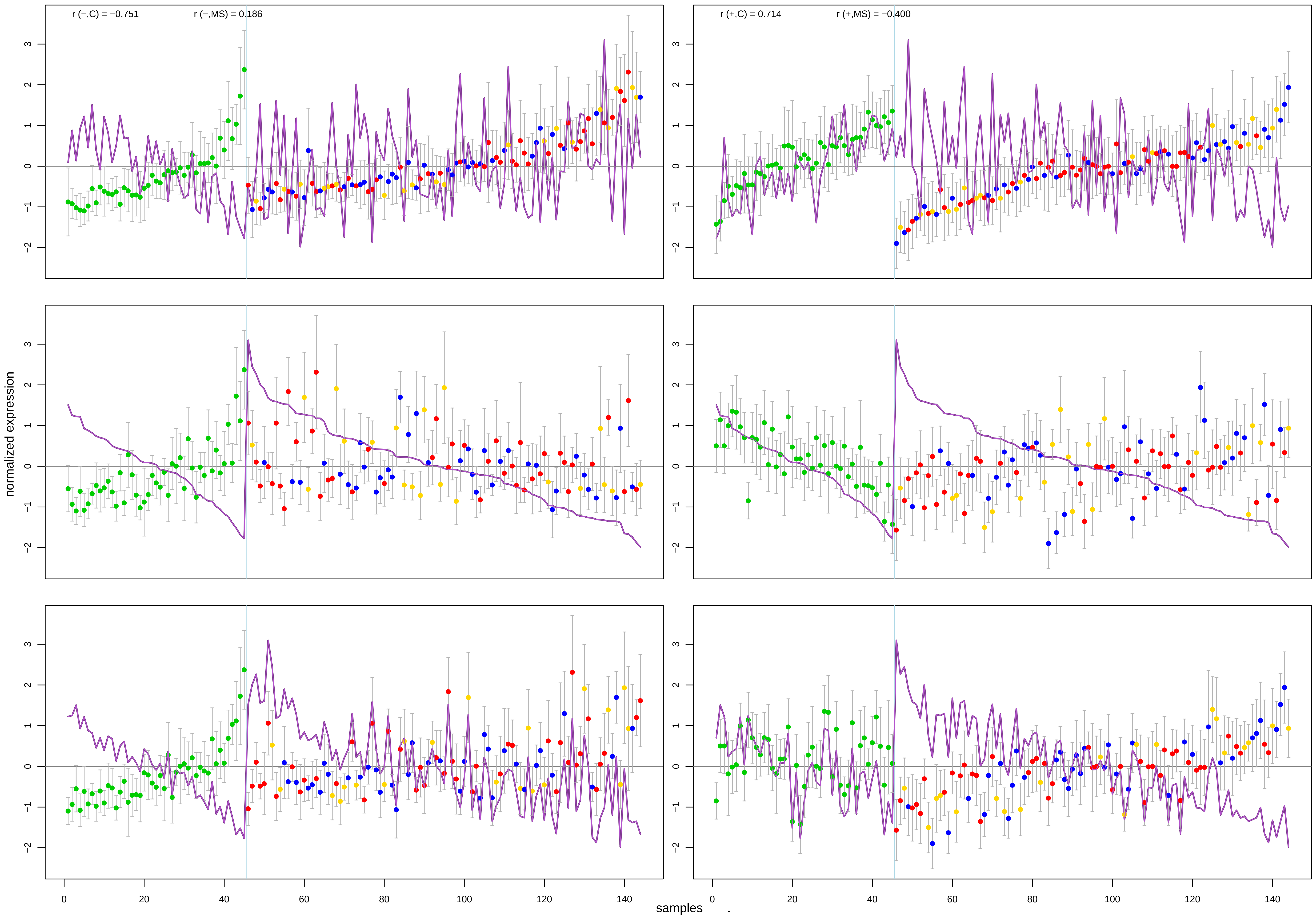
r (−,C) = −0.751
r (−,MS) = 0.186
r (+,C) = 0.714
r (+,MS) = −0.400
normalized expression
samples

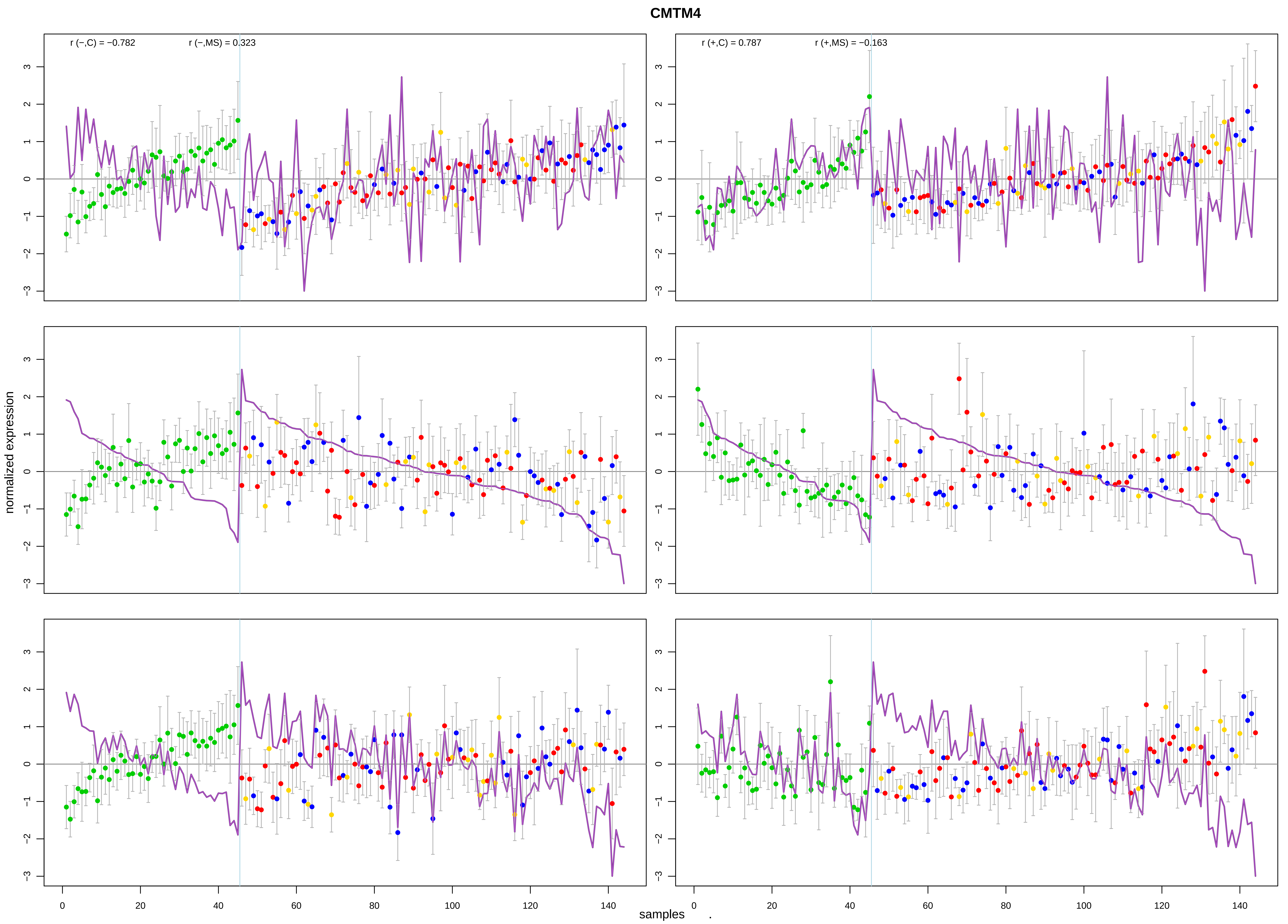
CMTM4
r (−,C) = −0.782
r (−,MS) = 0.323
r (+,C) = 0.787
r (+,MS) = −0.163
normalized expression
samples

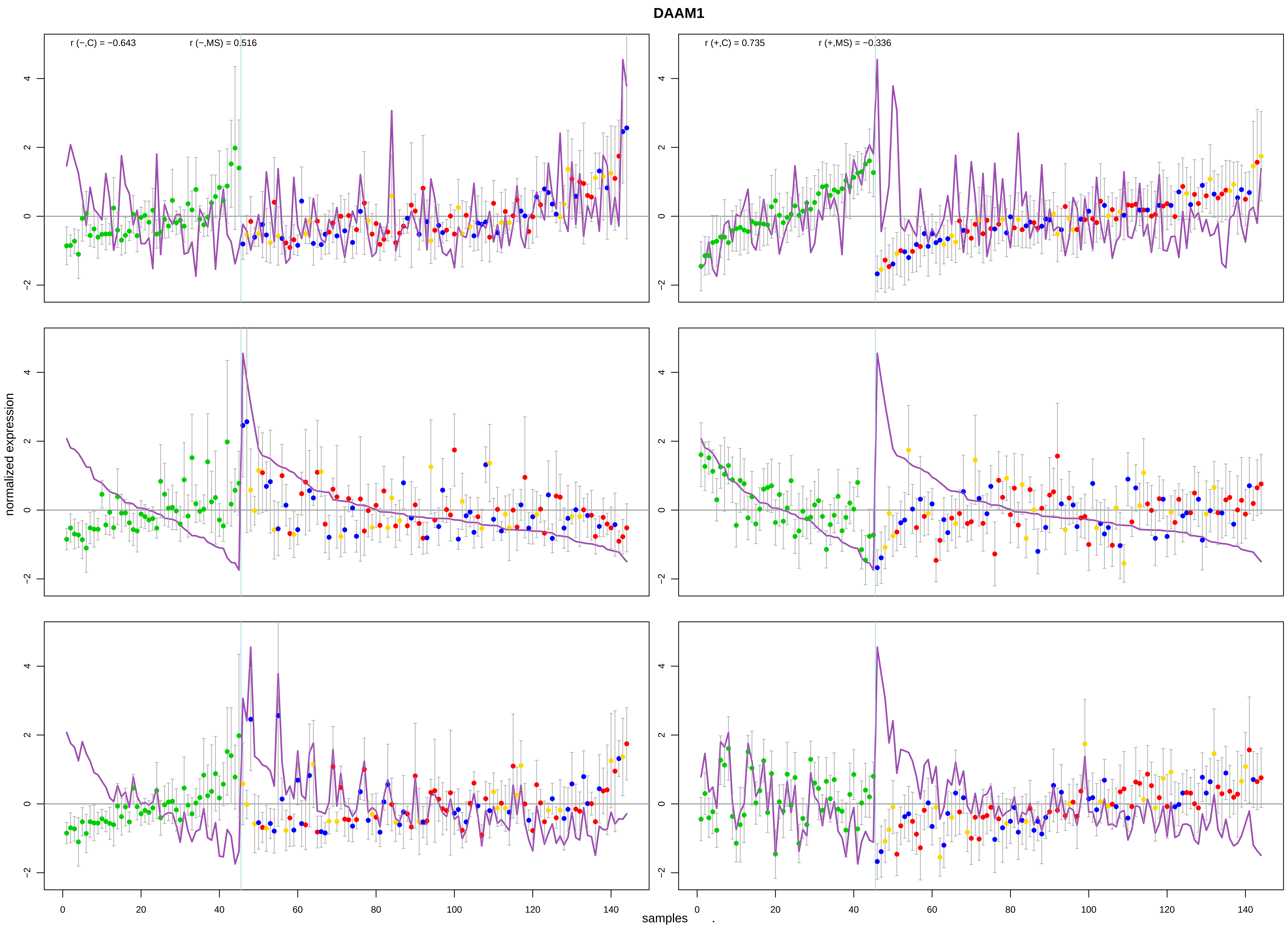
DAAM1
r (−,C) = −0.643
r (−,MS) = 0.516
r (+,C) = 0.735
r (+,MS) = −0.336
normalized expression
samples

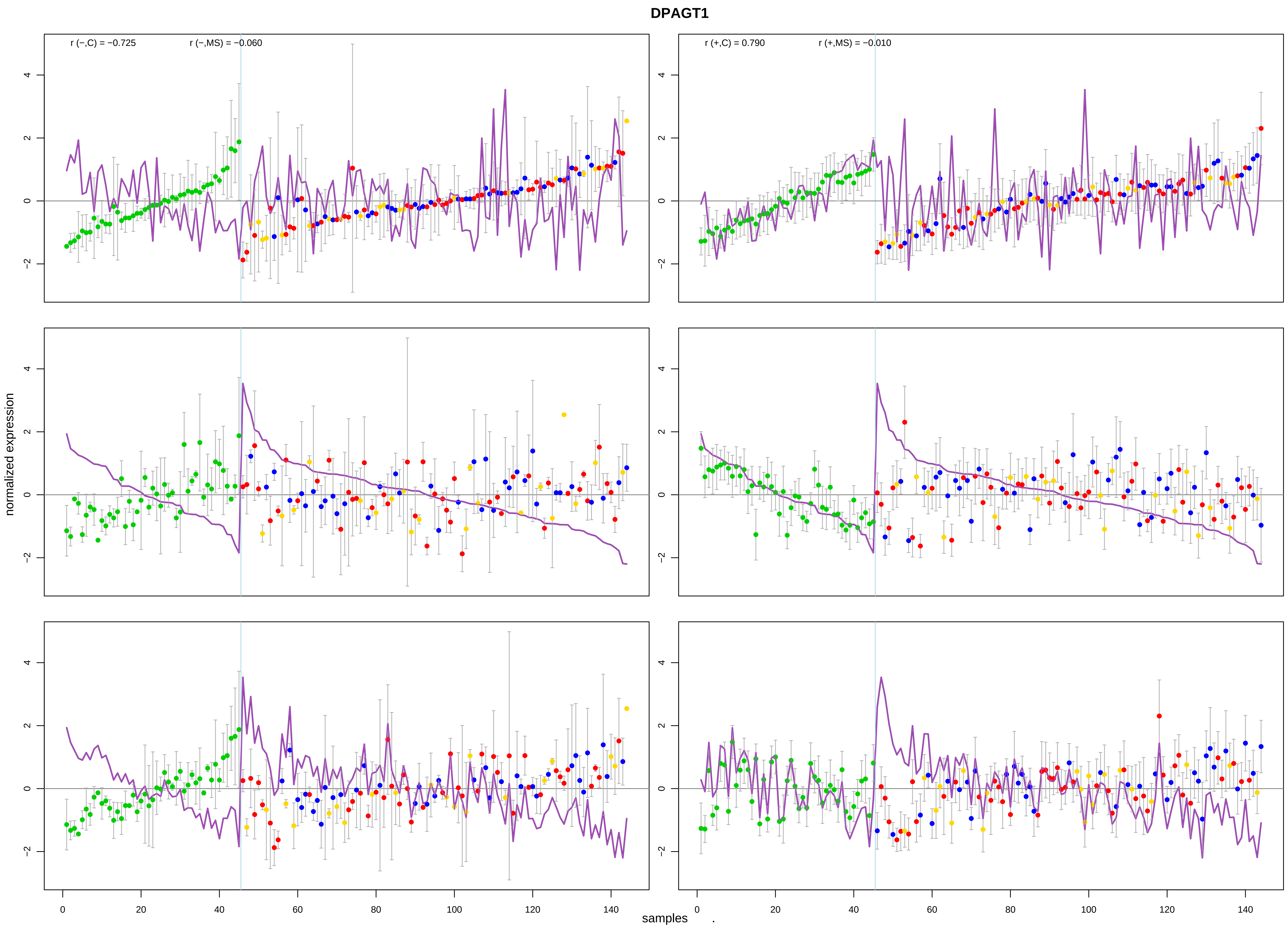
DPAGT1
r (−,C) = −0.725
r (−,MS) = −0.060
r (+,C) = 0.790
r (+,MS) = −0.010
normalized expression
samples
4
2
0
−2
0
20
40
60
80
100
120
140

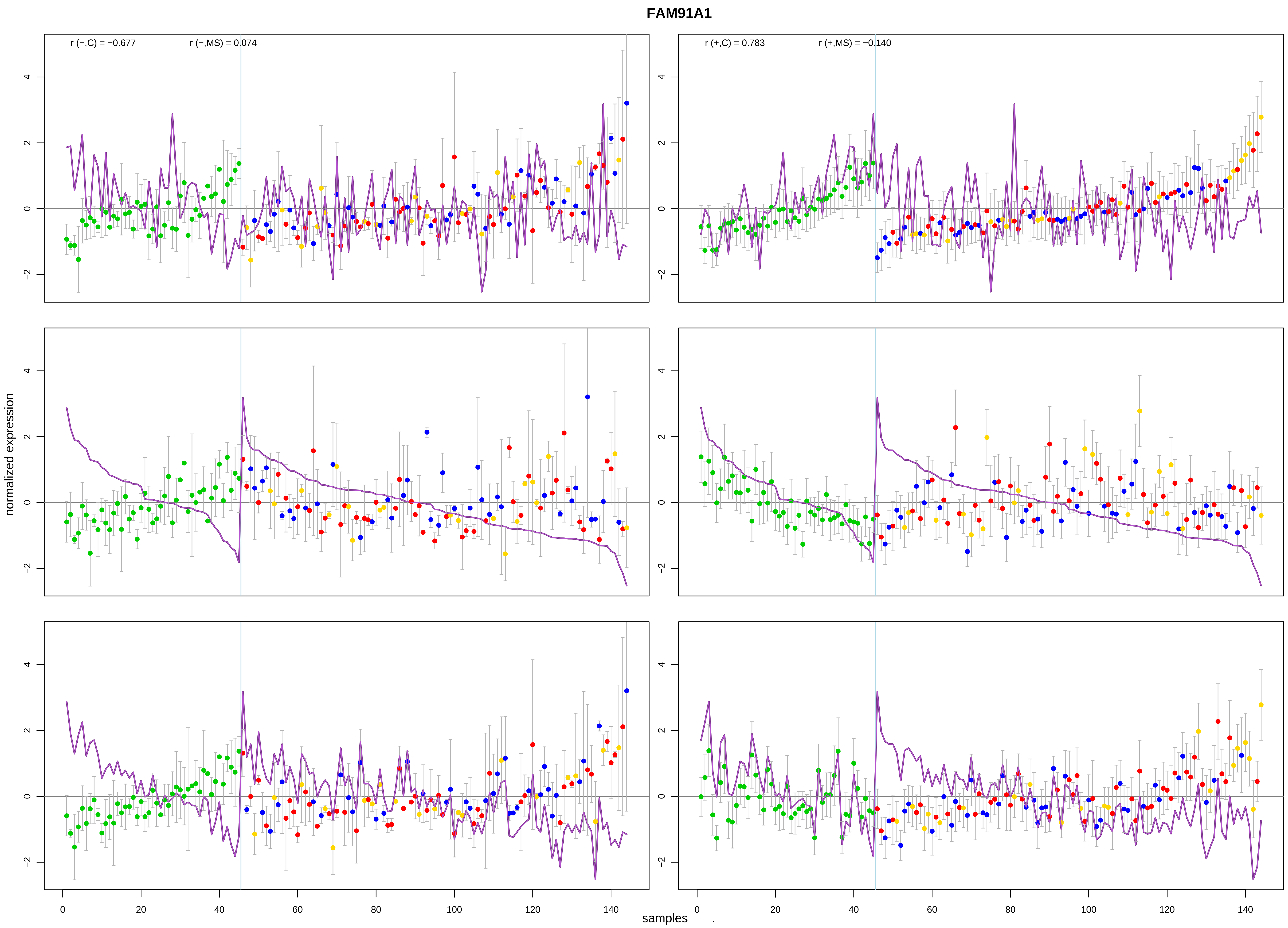

FAM91A1
r (−,C) = −0.677
r (−,MS) = 0.074
r (+,C) = 0.783
r (+,MS) = −0.140
normalized expression
samples

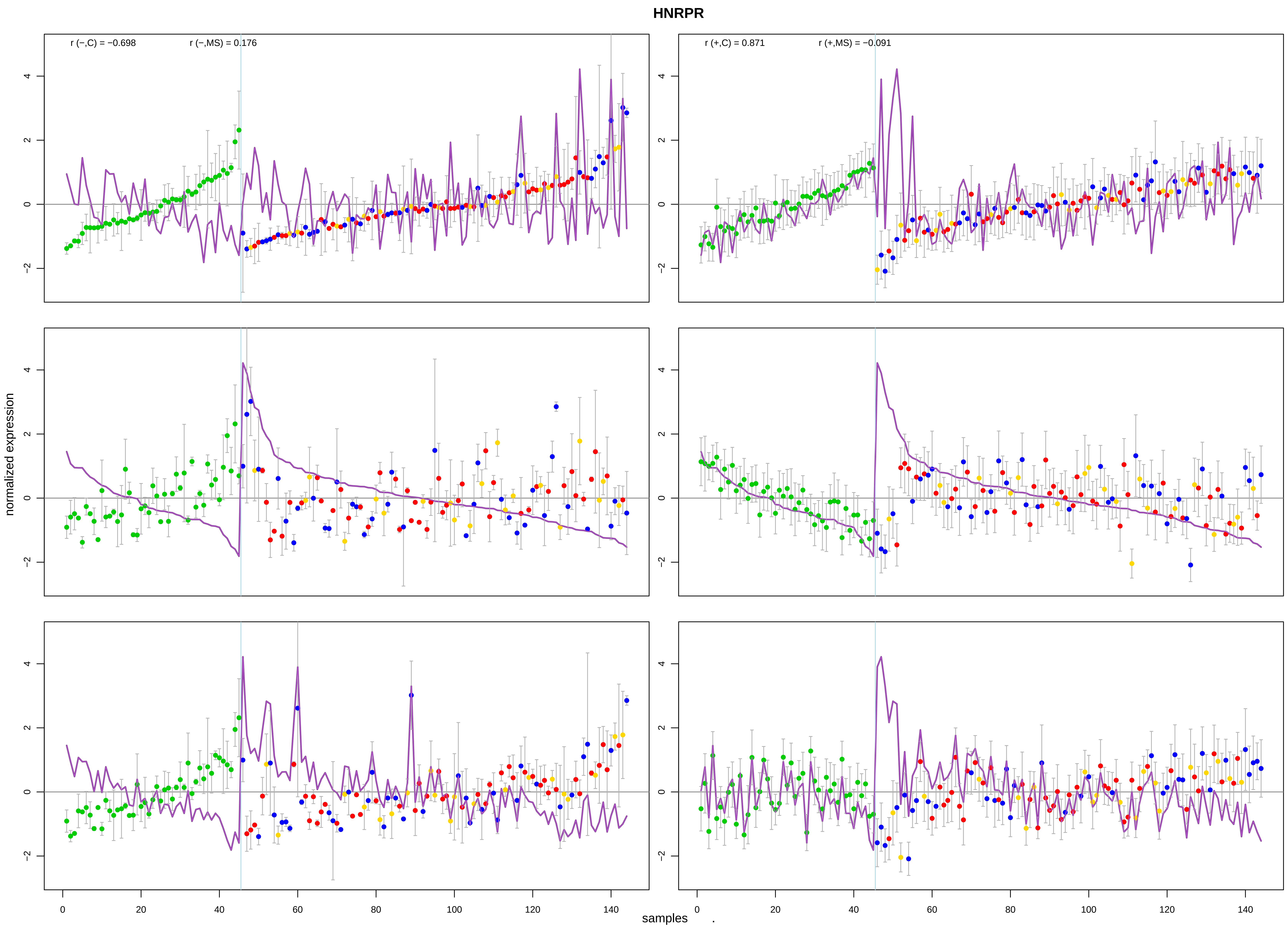

HNRPR
r (−,C) = −0.698
r (−,MS) = 0.176
r (+,C) = 0.871
r (+,MS) = −0.091
normalized expression
samples

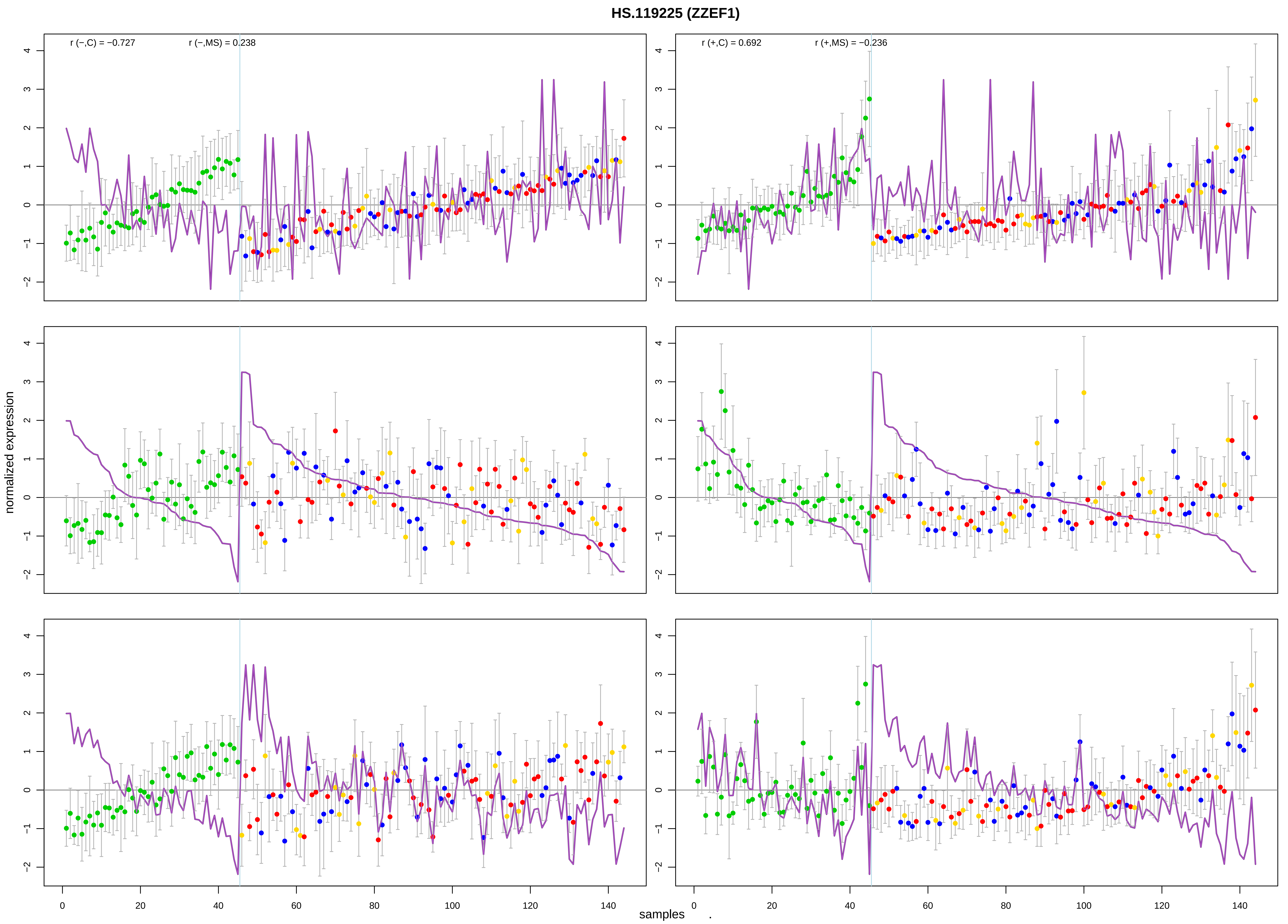

HS.119225 (ZZEF1)
r (−,C) = −0.727
r (−,MS) = 0.238
r (+,C) = 0.692
r (+,MS) = −0.236
normalized expression
samples

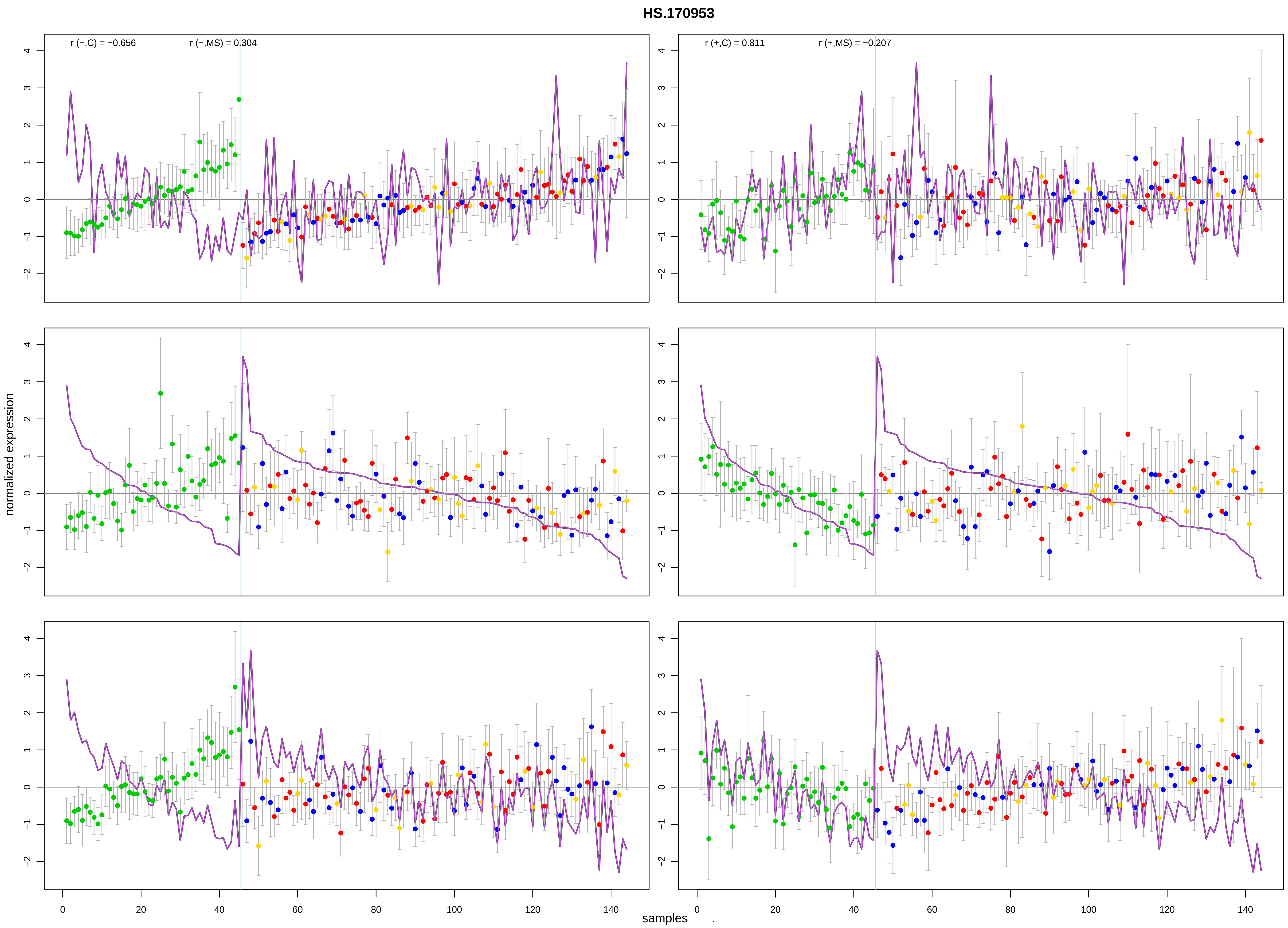
HS.170953
r (−,C) = −0.656
r (−,MS) = 0.304
r (+,C) = 0.811
r (+,MS) = −0.207
normalized expression
samples

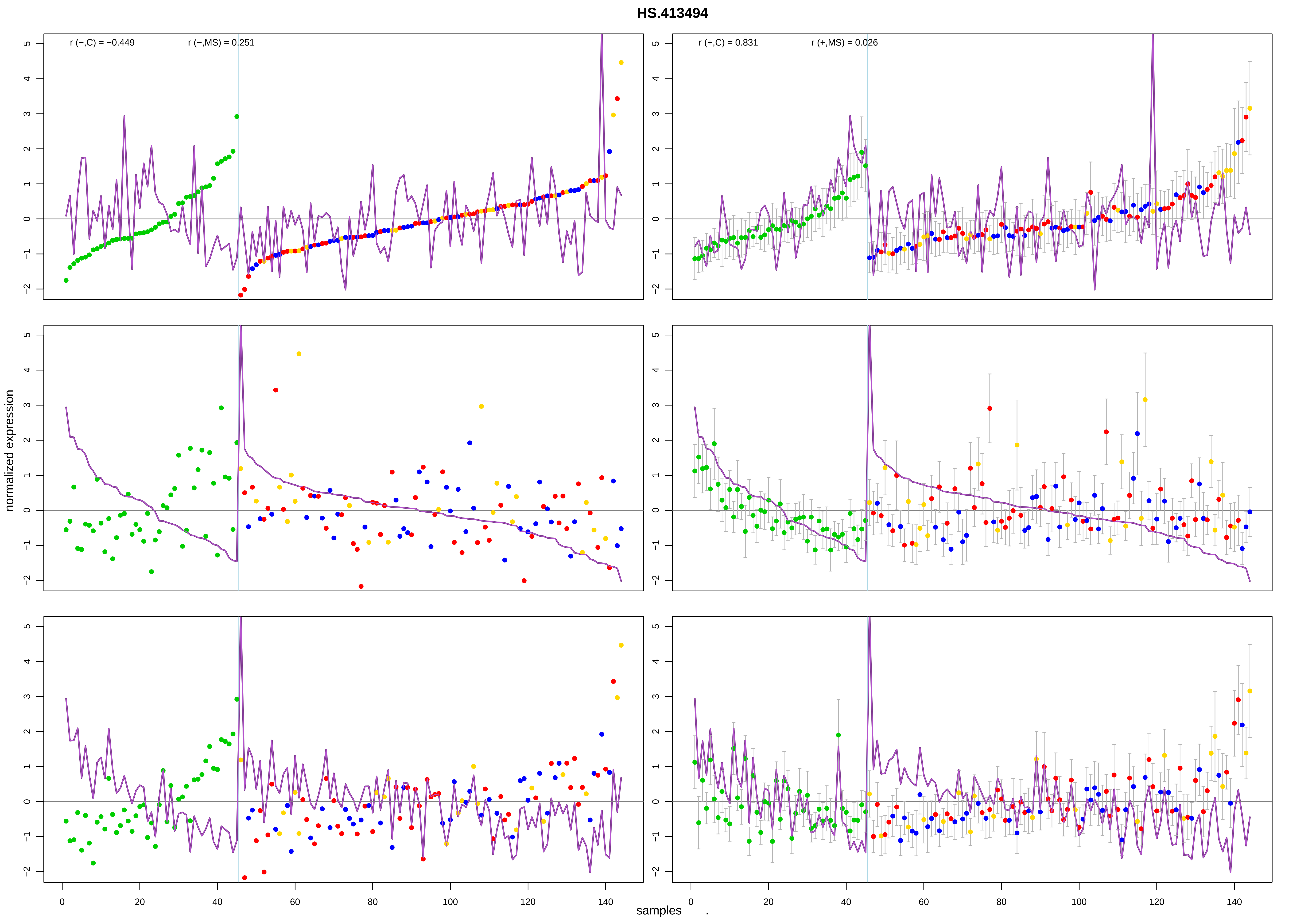
HS.413494
r (−,C) = −0.449
r (−,MS) = 0.251
r (+,C) = 0.831
r (+,MS) = 0.026
normalized expression
samples

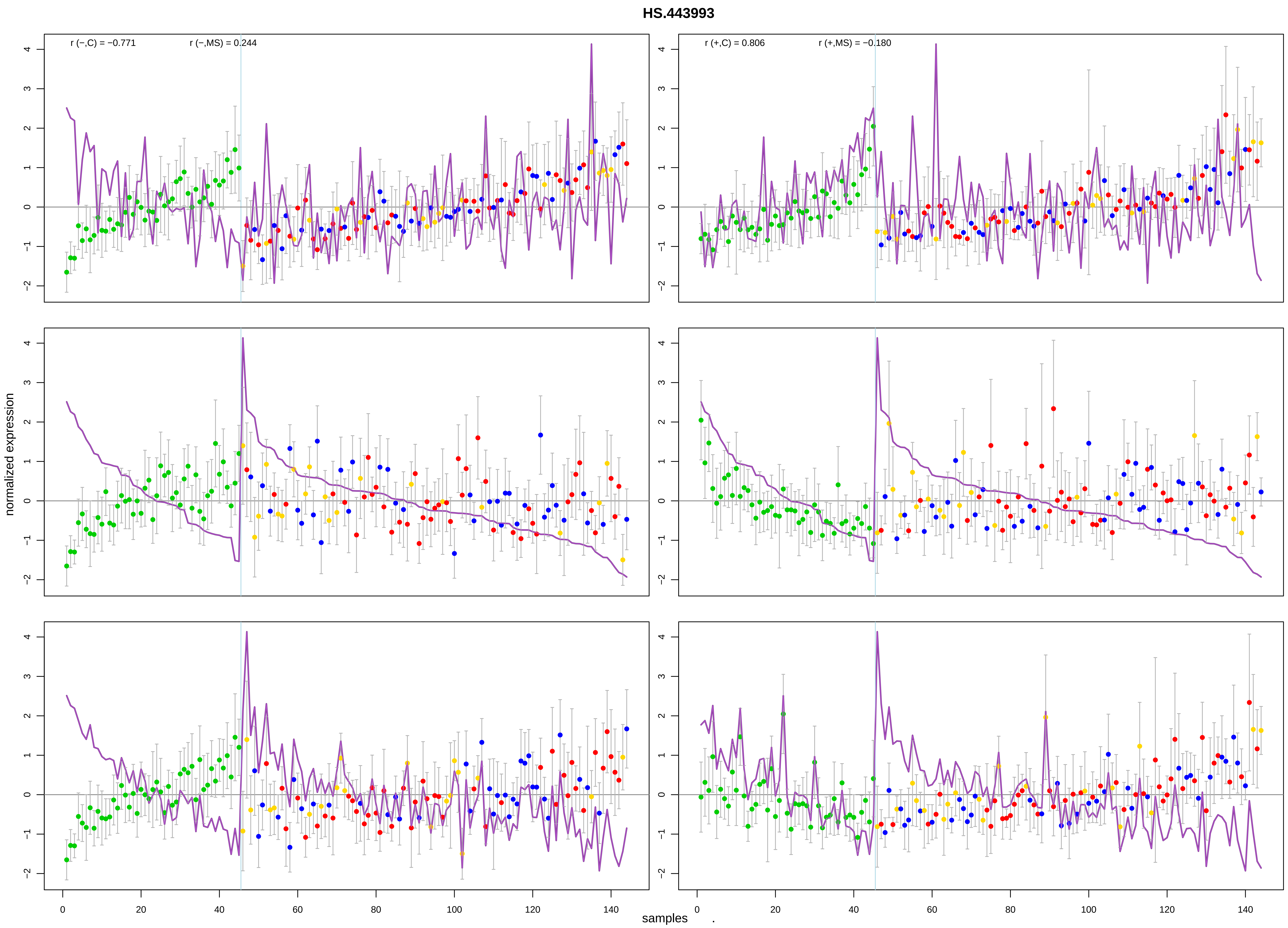
HS.443993
r (−,C) = −0.771
r (−,MS) = 0.244
r (+,C) = 0.806
r (+,MS) = −0.180
normalized expression
samples

HS.495215 (TCRA)

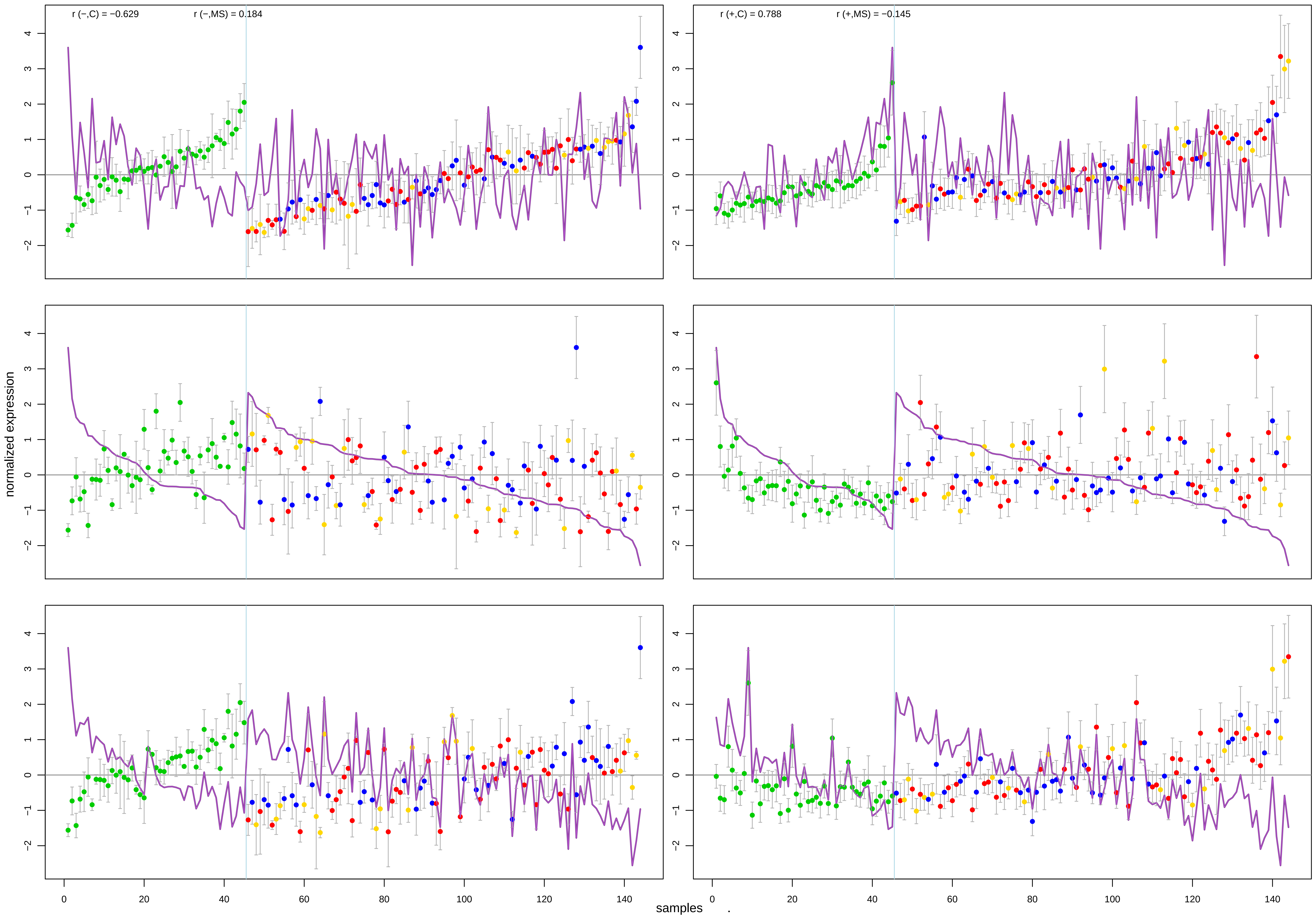

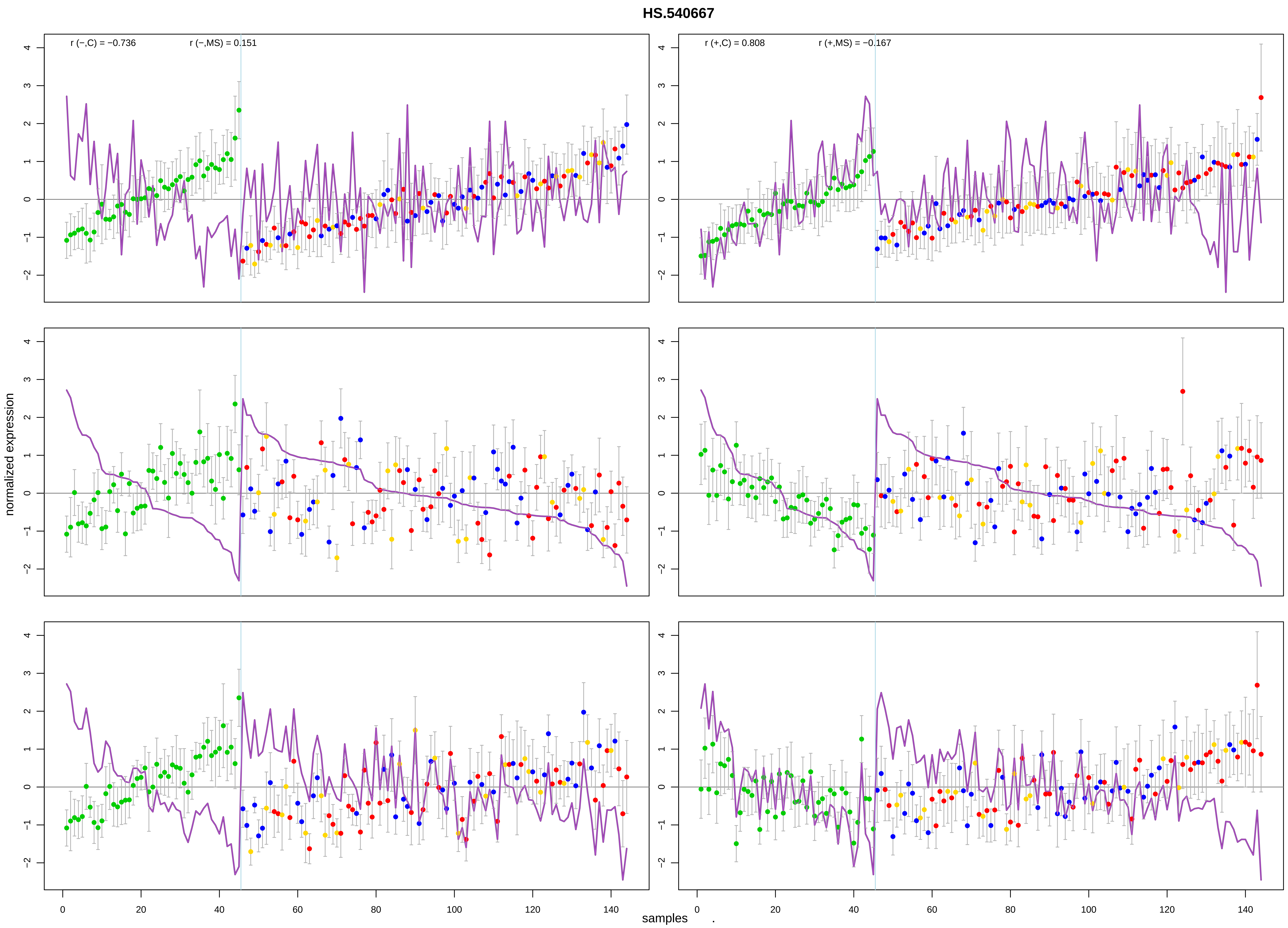
HS.540667
r (−,C) = −0.736
r (−,MS) = 0.151
r (+,C) = 0.808
r (+,MS) = −0.167
normalized expression
samples

HS.541221

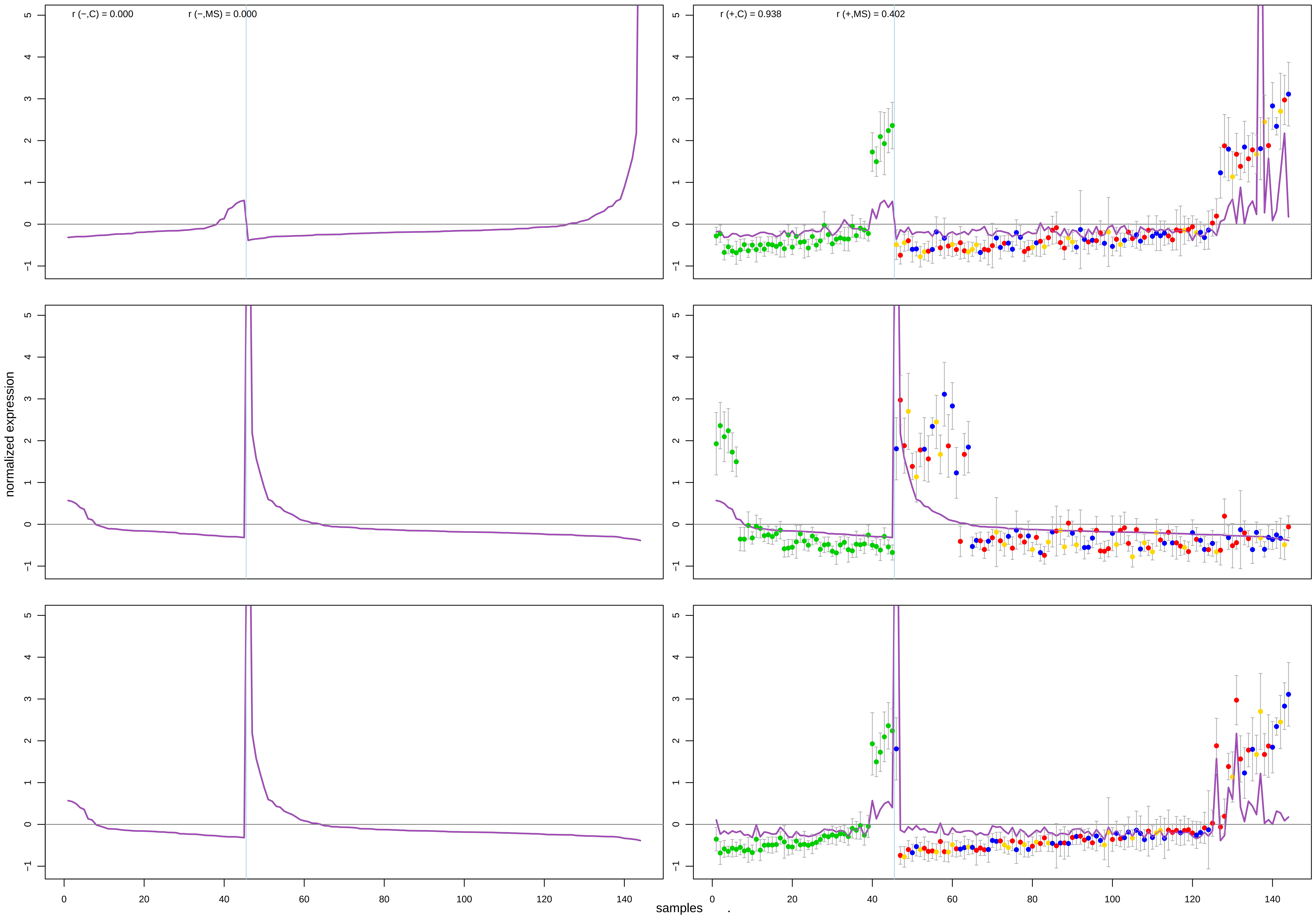
r (−,C) = 0.000
r (−,MS) = 0.000
r (+,C) = 0.938
r (+,MS) = 0.402
normalized expression
samples

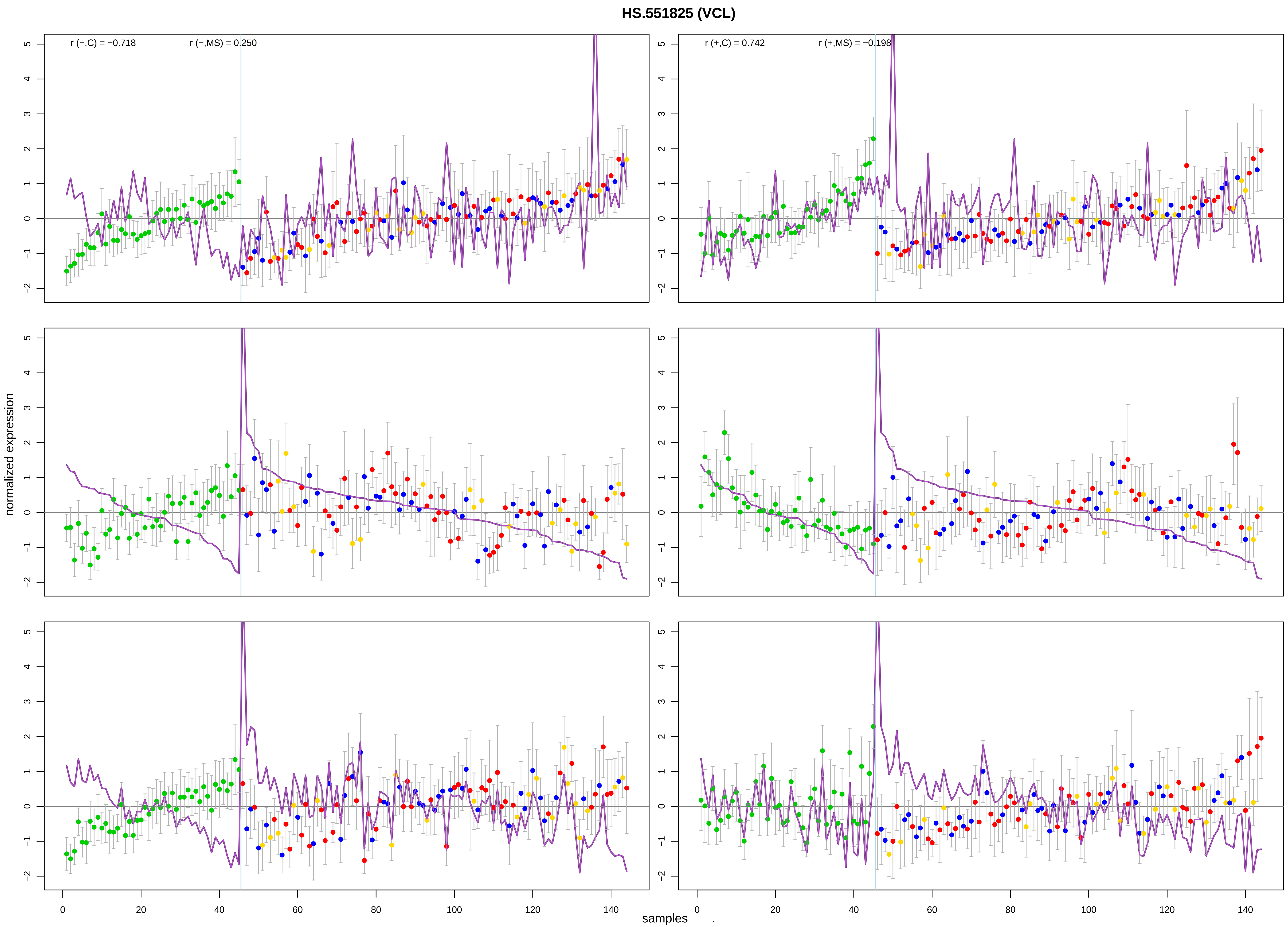

HS.551825 (VCL)
r (−,C) = −0.718
r (−,MS) = 0.250
r (+,C) = 0.742
r (+,MS) = −0.198
normalized expression
samples

HS.571125

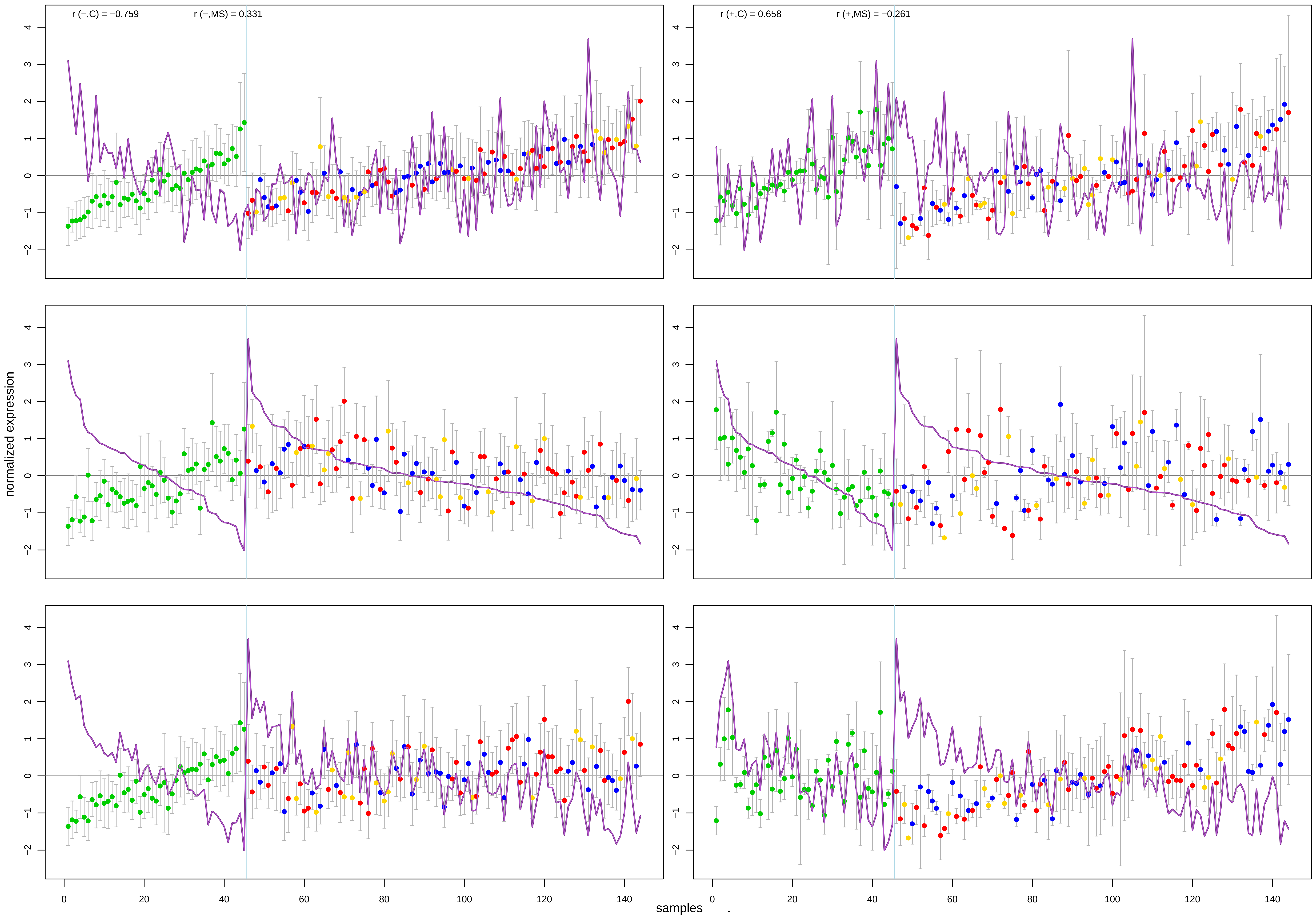
r (−,C) = −0.759
r (−,MS) = 0.331
r (+,C) = 0.658
r (+,MS) = −0.261
normalized expression
samples

HS.574542 (ALG8)

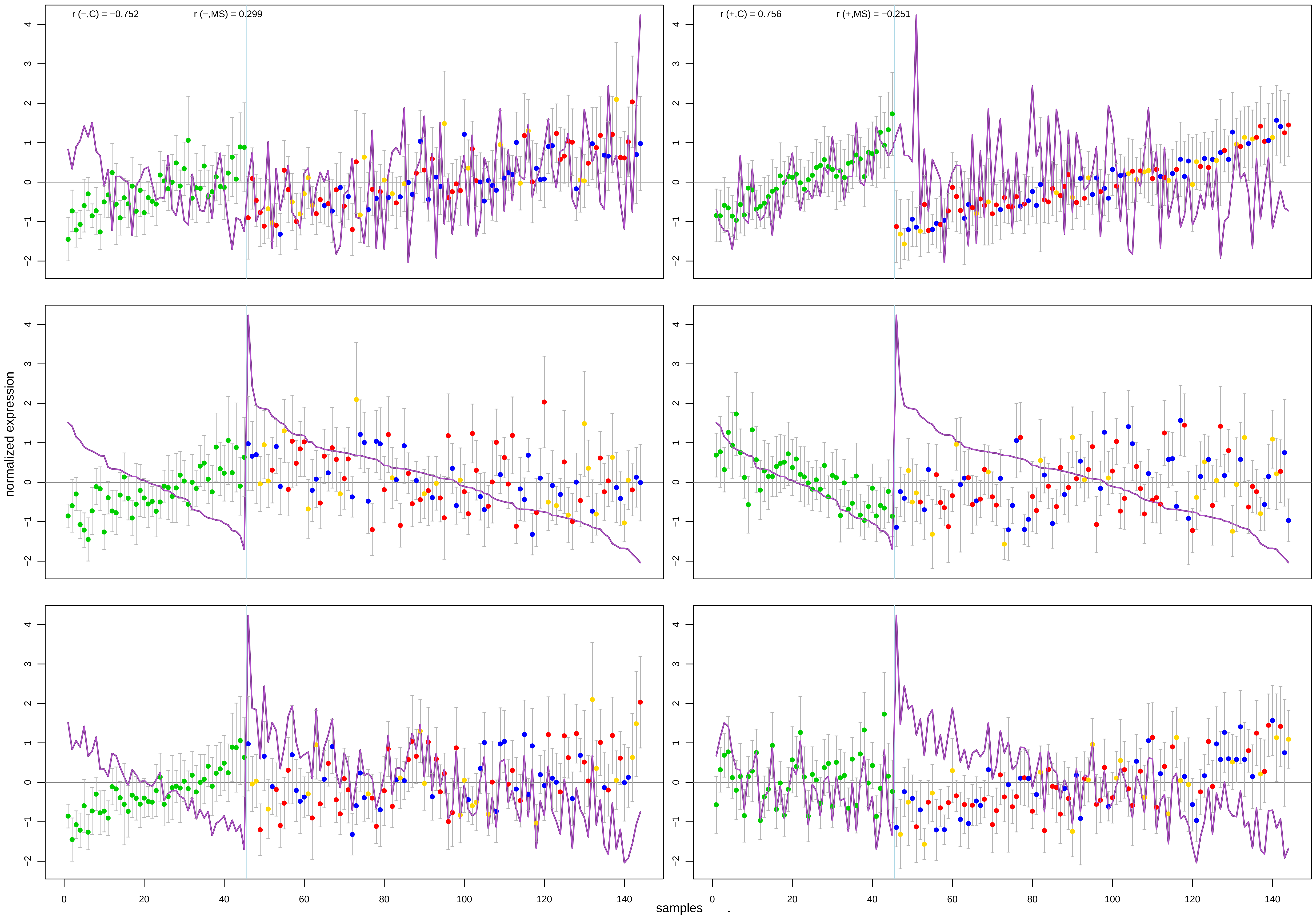

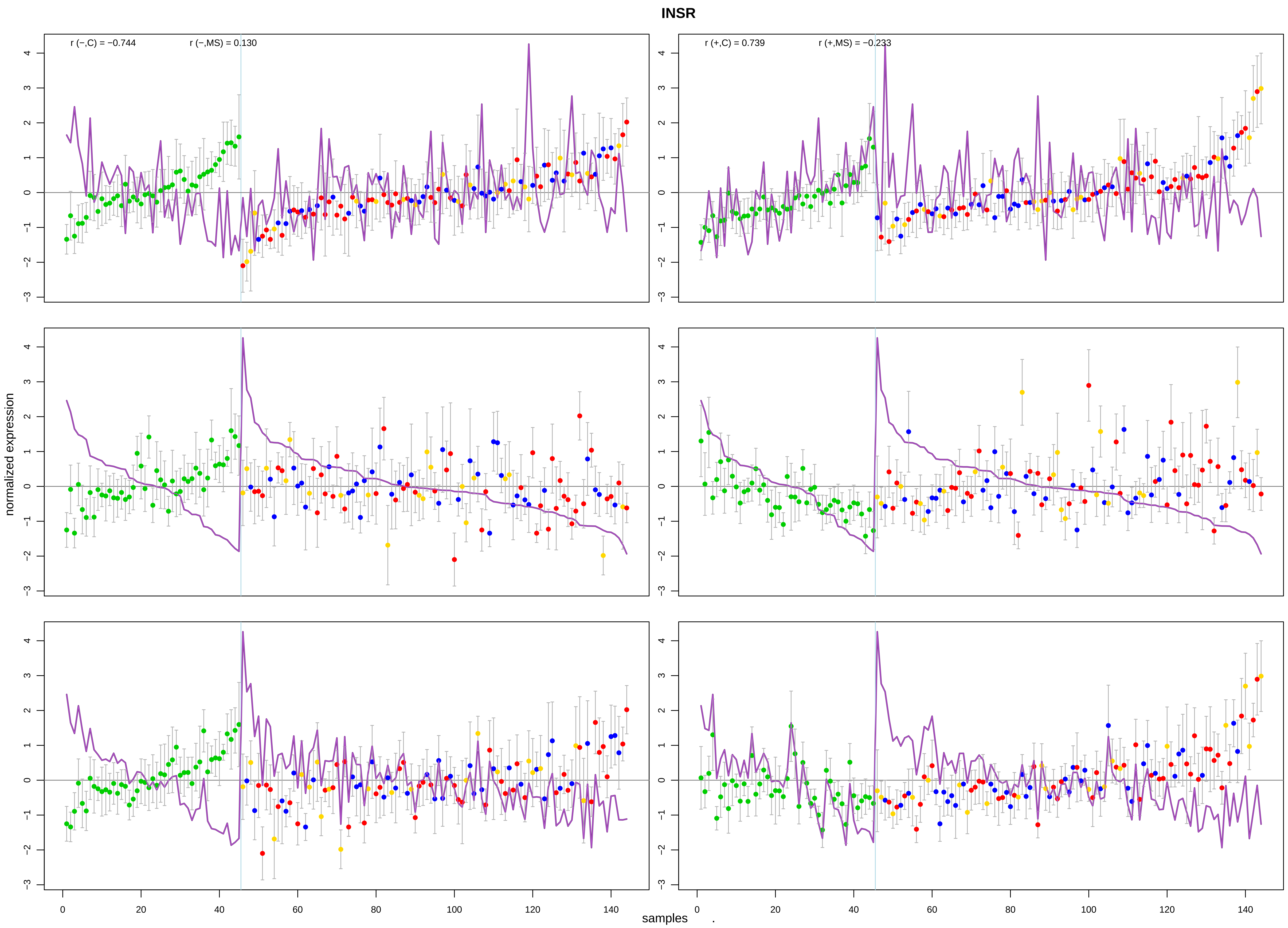

INSR
r (−,C) = −0.744
r (−,MS) = 0.130
r (+,C) = 0.739
r (+,MS) = −0.233
normalized expression
samples

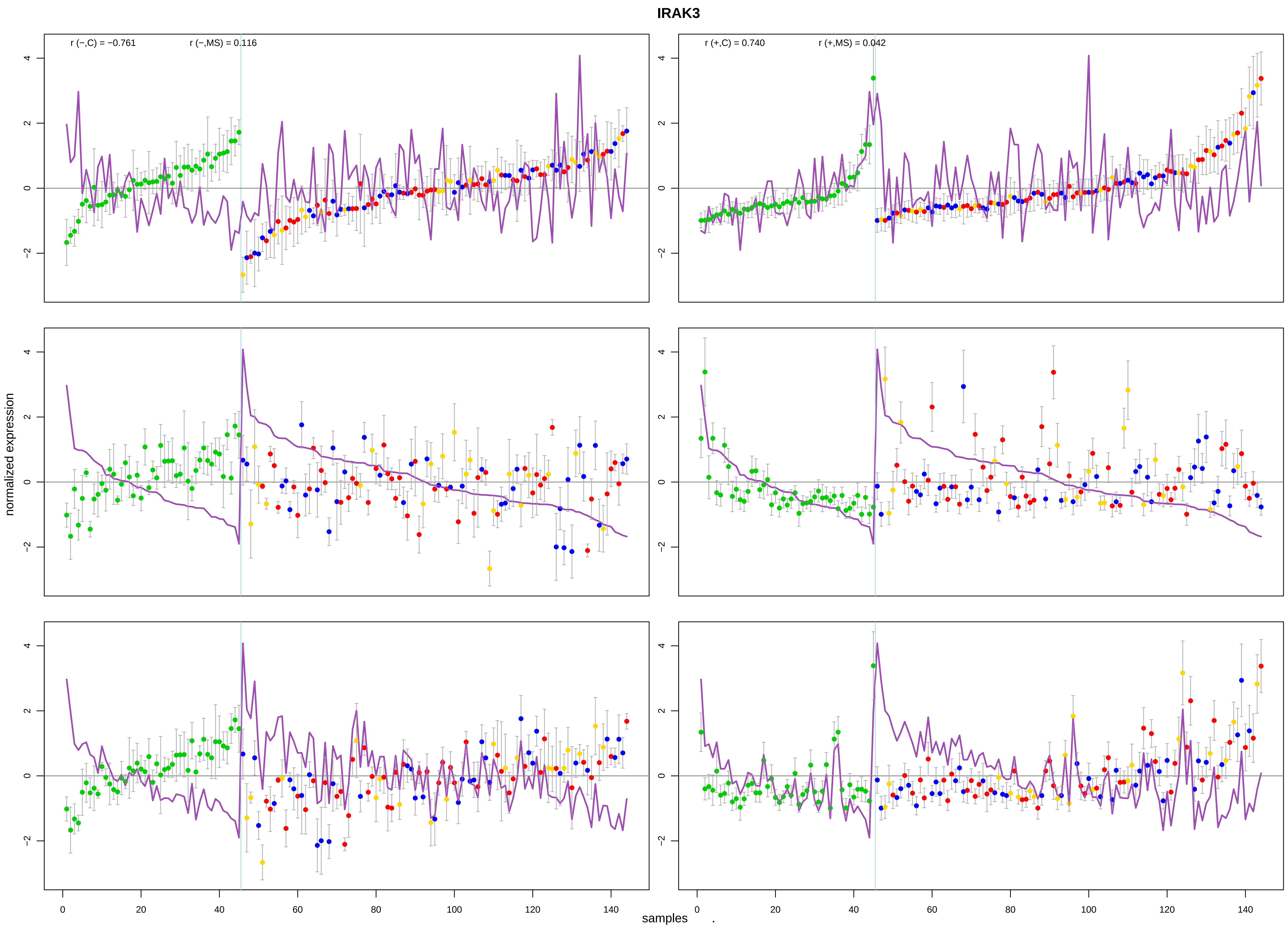

IRAK3
r (−,C) = −0.761
r (−,MS) = 0.116
r (+,C) = 0.740
r (+,MS) = 0.042
normalized expression
samples

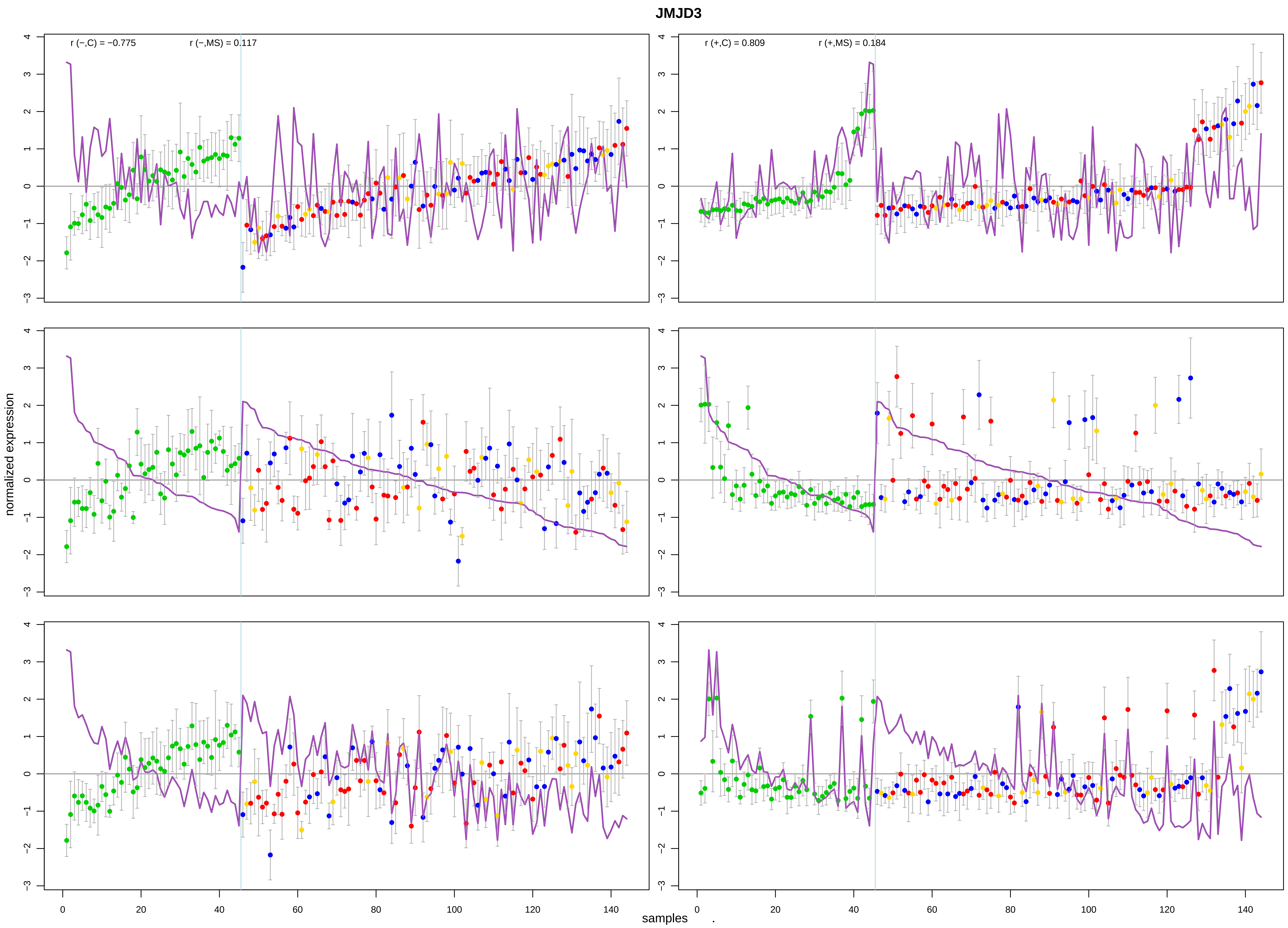

JMJD3
r (−,C) = −0.775
r (−,MS) = 0.117
r (+,C) = 0.809
r (+,MS) = 0.184
normalized expression
samples

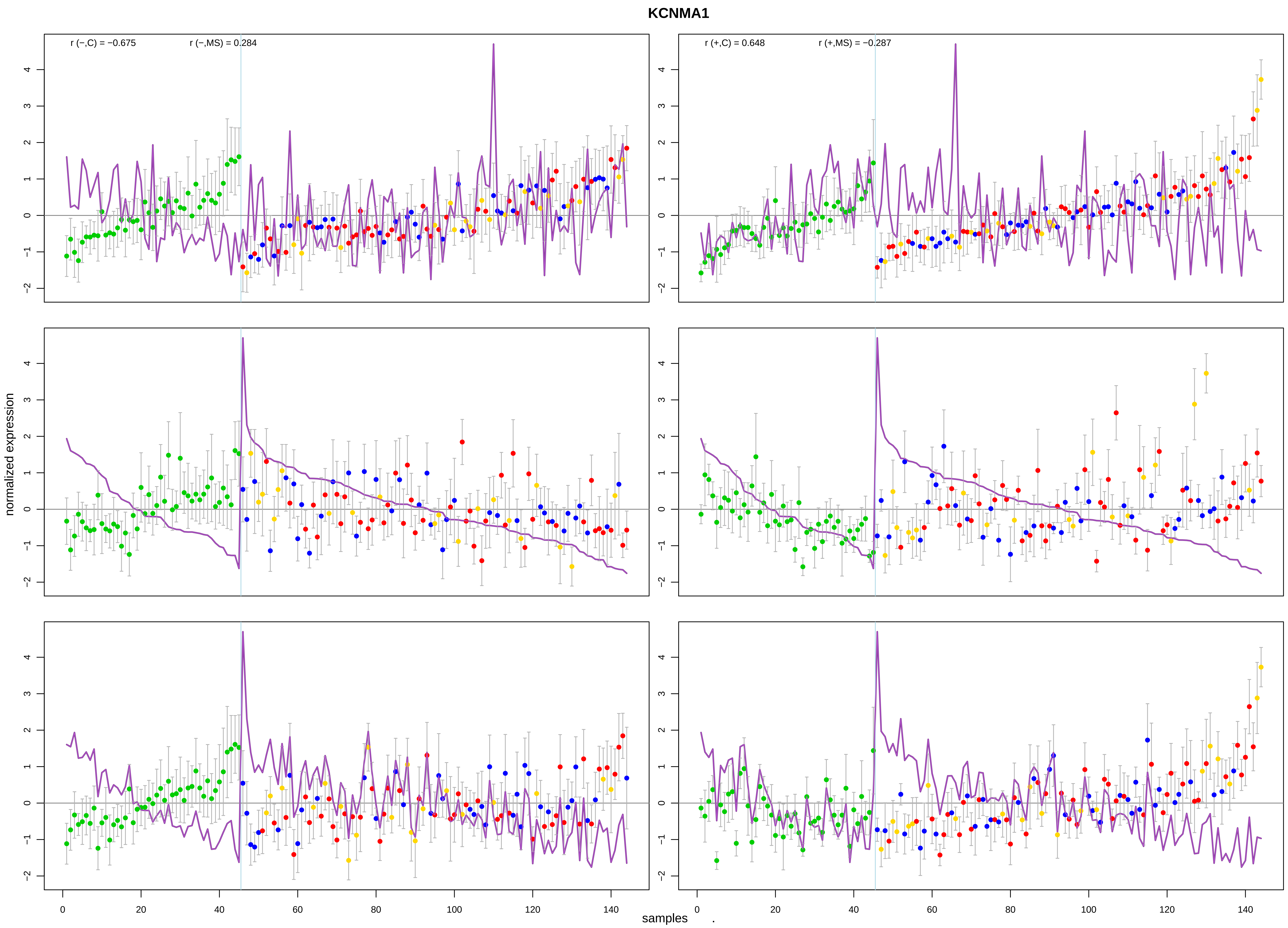
KCNMA1
r (−,C) = −0.675
r (−,MS) = 0.284
r (+,C) = 0.648
r (+,MS) = −0.287
normalized expression
samples

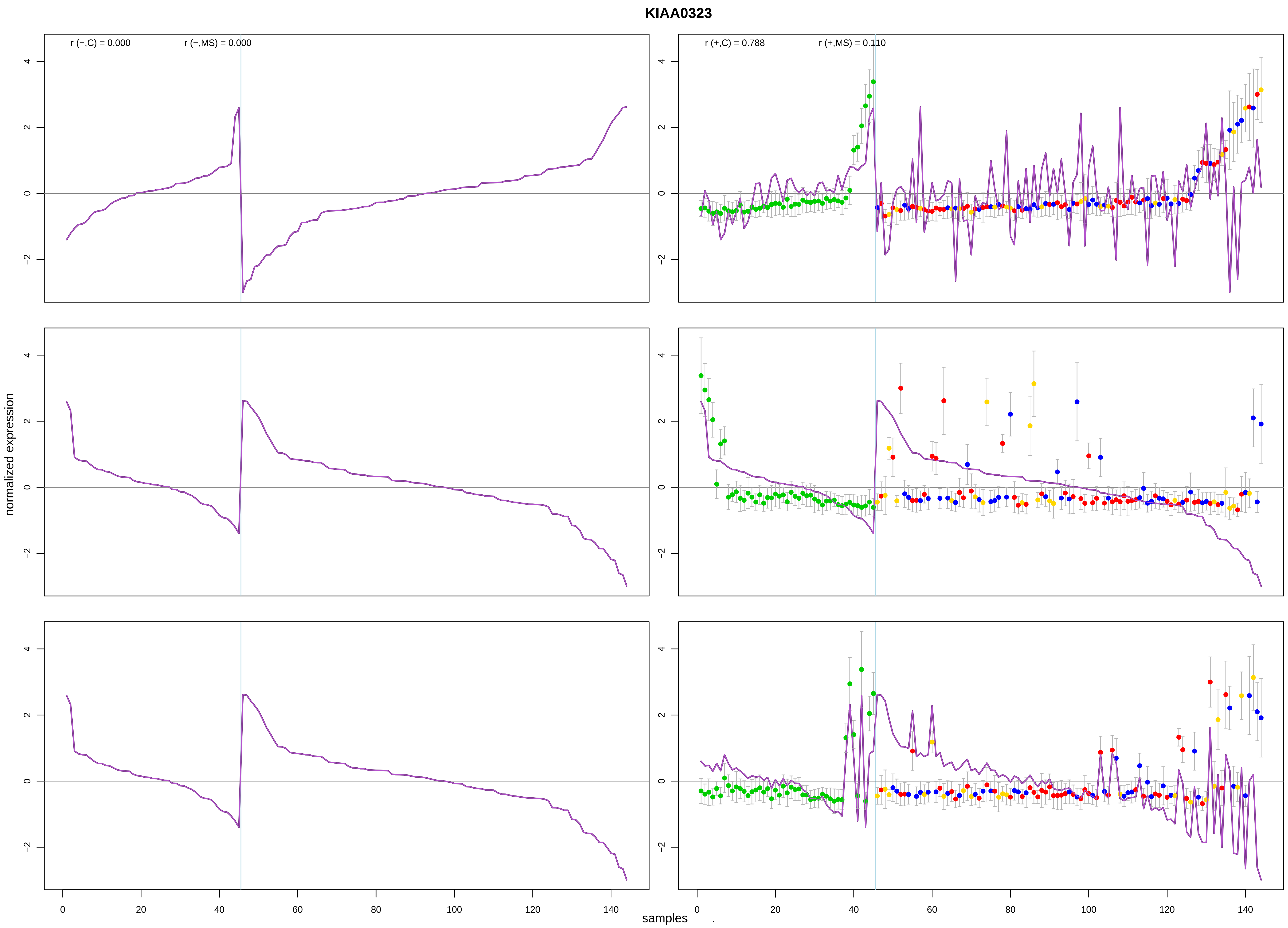
KIAA0323
r (−,C) = 0.000
r (−,MS) = 0.000
r (+,C) = 0.788
r (+,MS) = 0.110
normalized expression
samples
0
20
40
60
80
100
120
140
4
2
0
−2

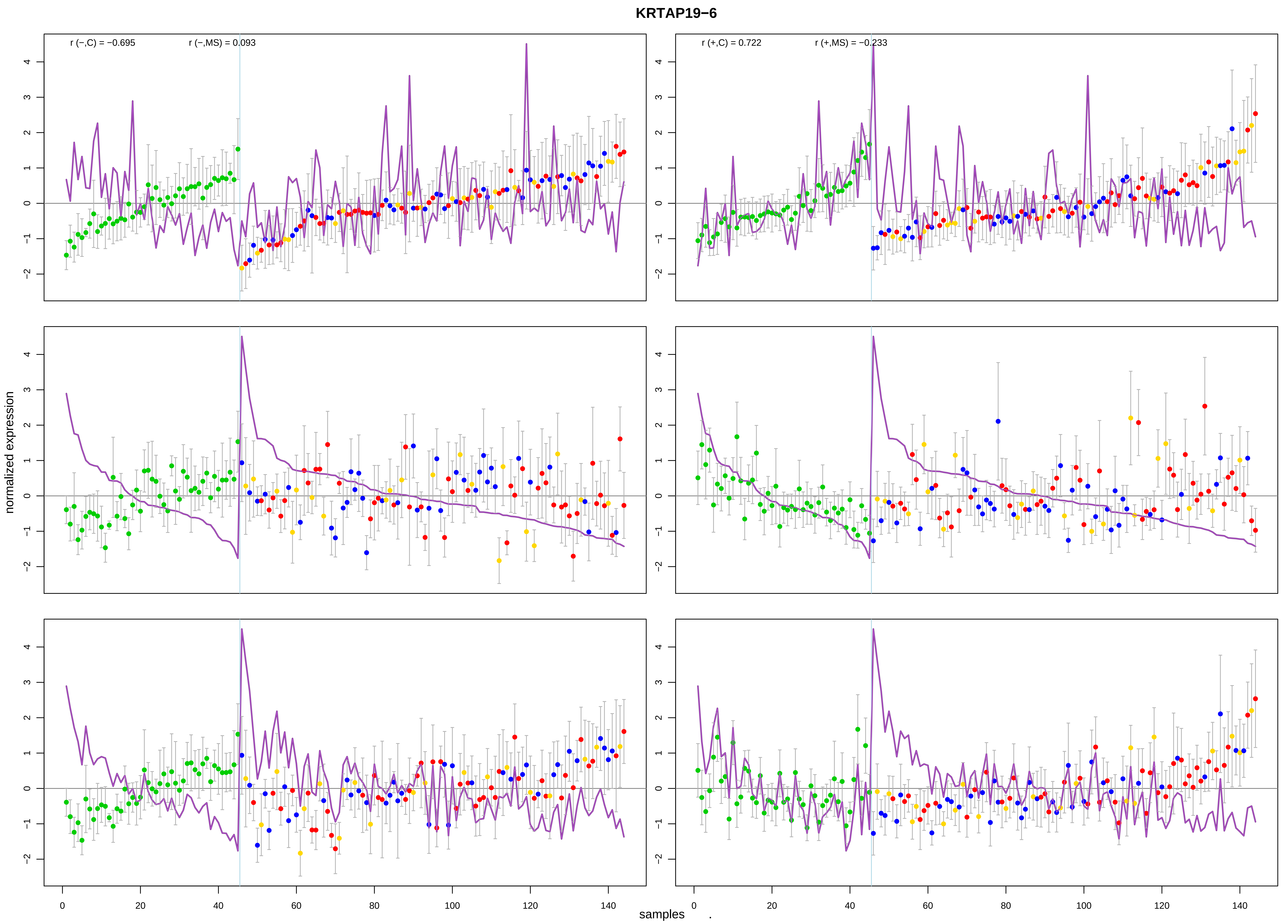
KRTAP19−6
r (−,C) = −0.695
r (−,MS) = 0.093
r (+,C) = 0.722
r (+,MS) = −0.233
normalized expression
samples

LOC387870

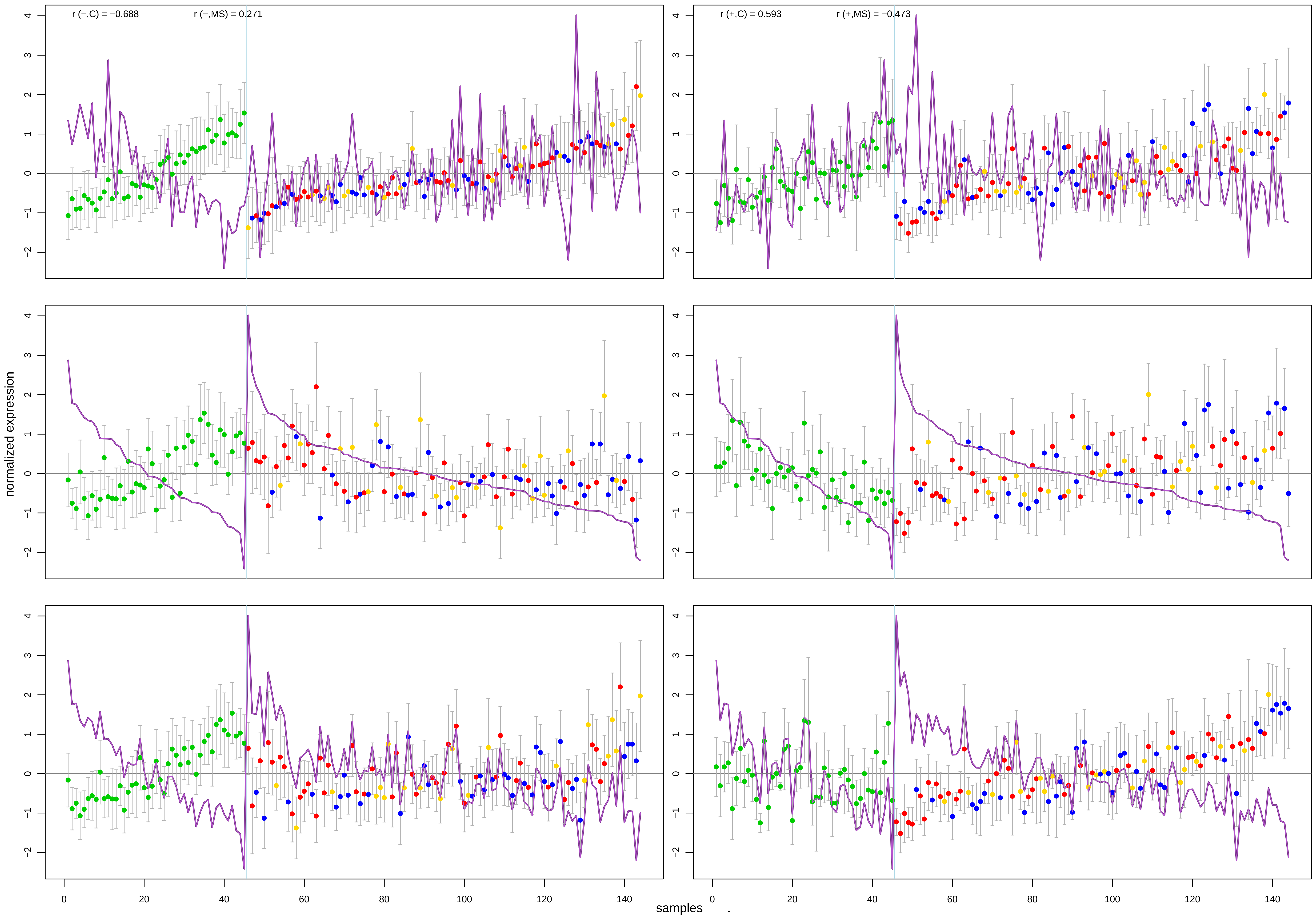

r (−,C) = −0.688
r (−,MS) = 0.271
r (+,C) = 0.593
r (+,MS) = −0.473
normalized expression
samples

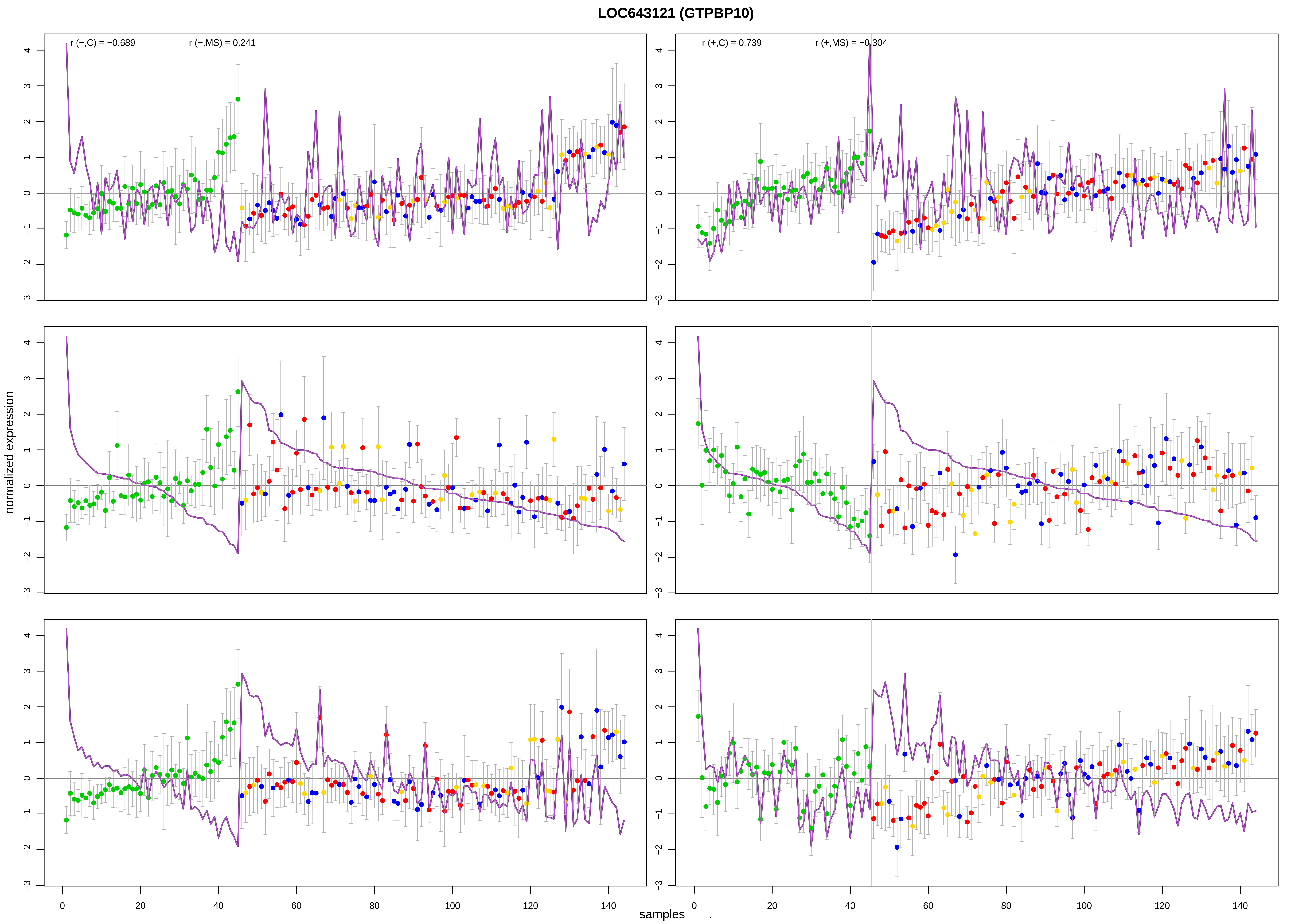

LOC643121 (GTPBP10)
r (−,C) = −0.689
r (−,MS) = 0.241
r (+,C) = 0.739
r (+,MS) = −0.304
normalized expression
samples

LOC648716

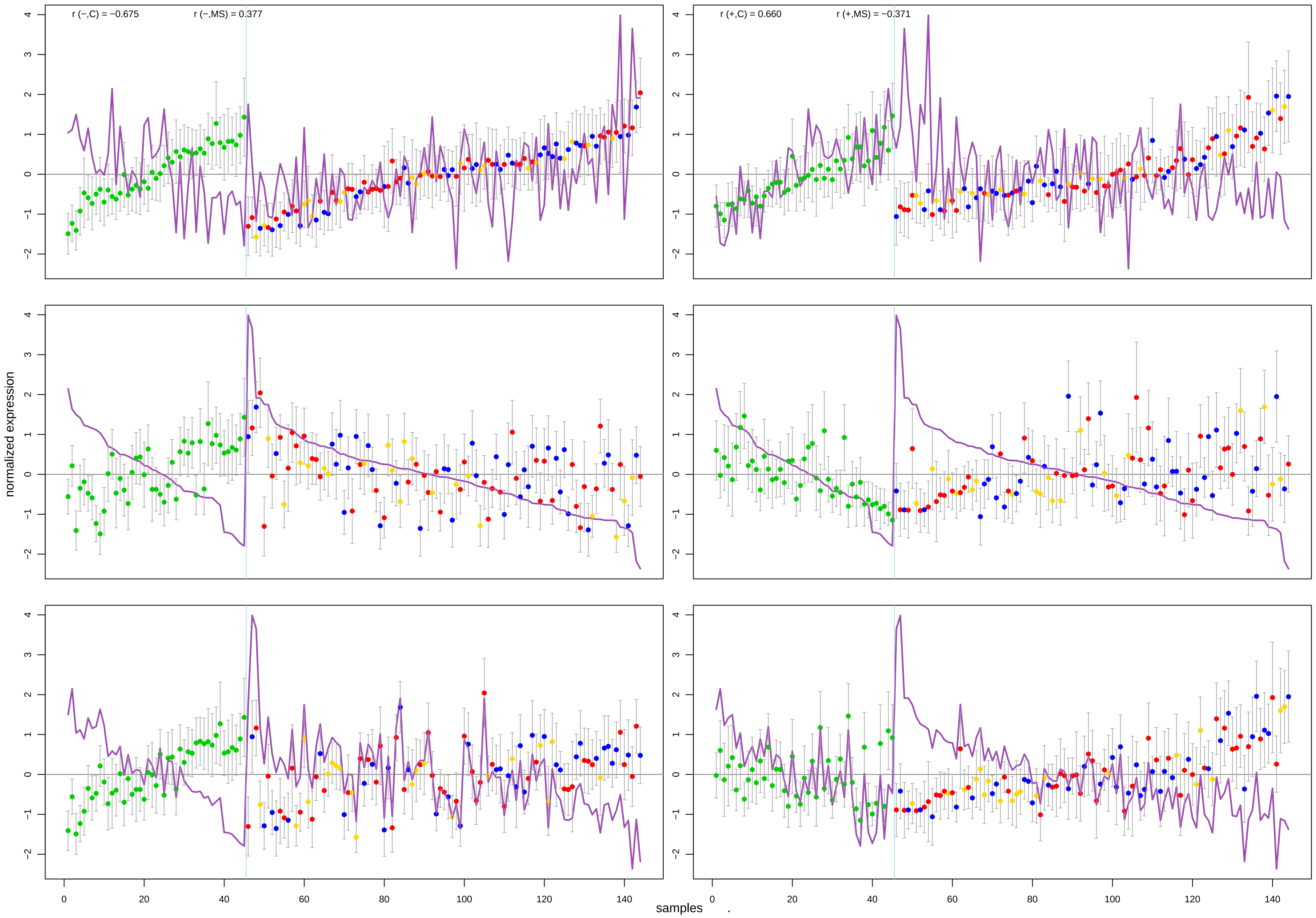

r (−,C) = −0.675
r (−,MS) = 0.377
r (+,C) = 0.660
r (+,MS) = −0.371
normalized expression
samples

LOC648814

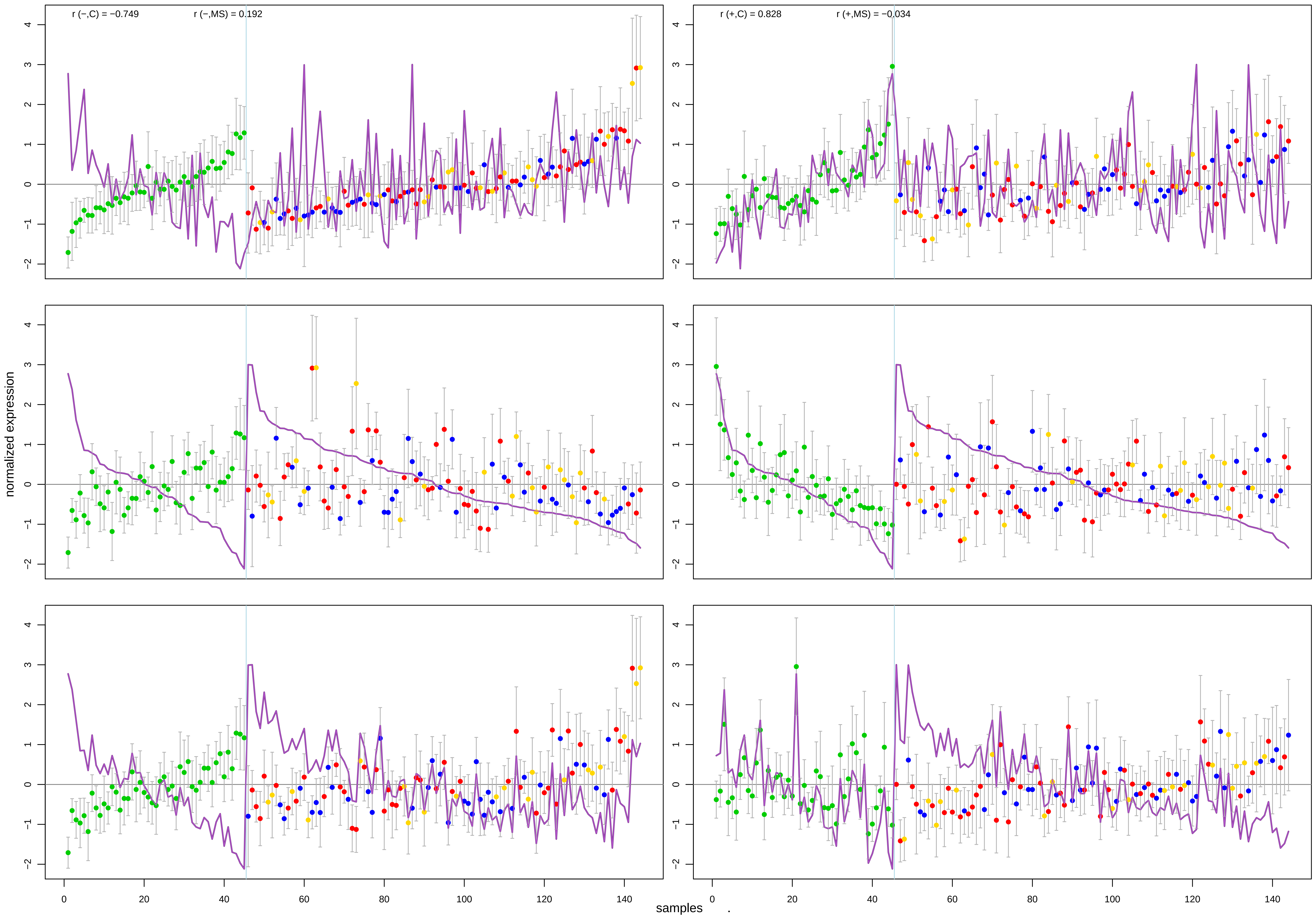

r (−,C) = −0.749
r (−,MS) = 0.192
r (+,C) = 0.828
r (+,MS) = −0.034
normalized expression
samples

LOC651316

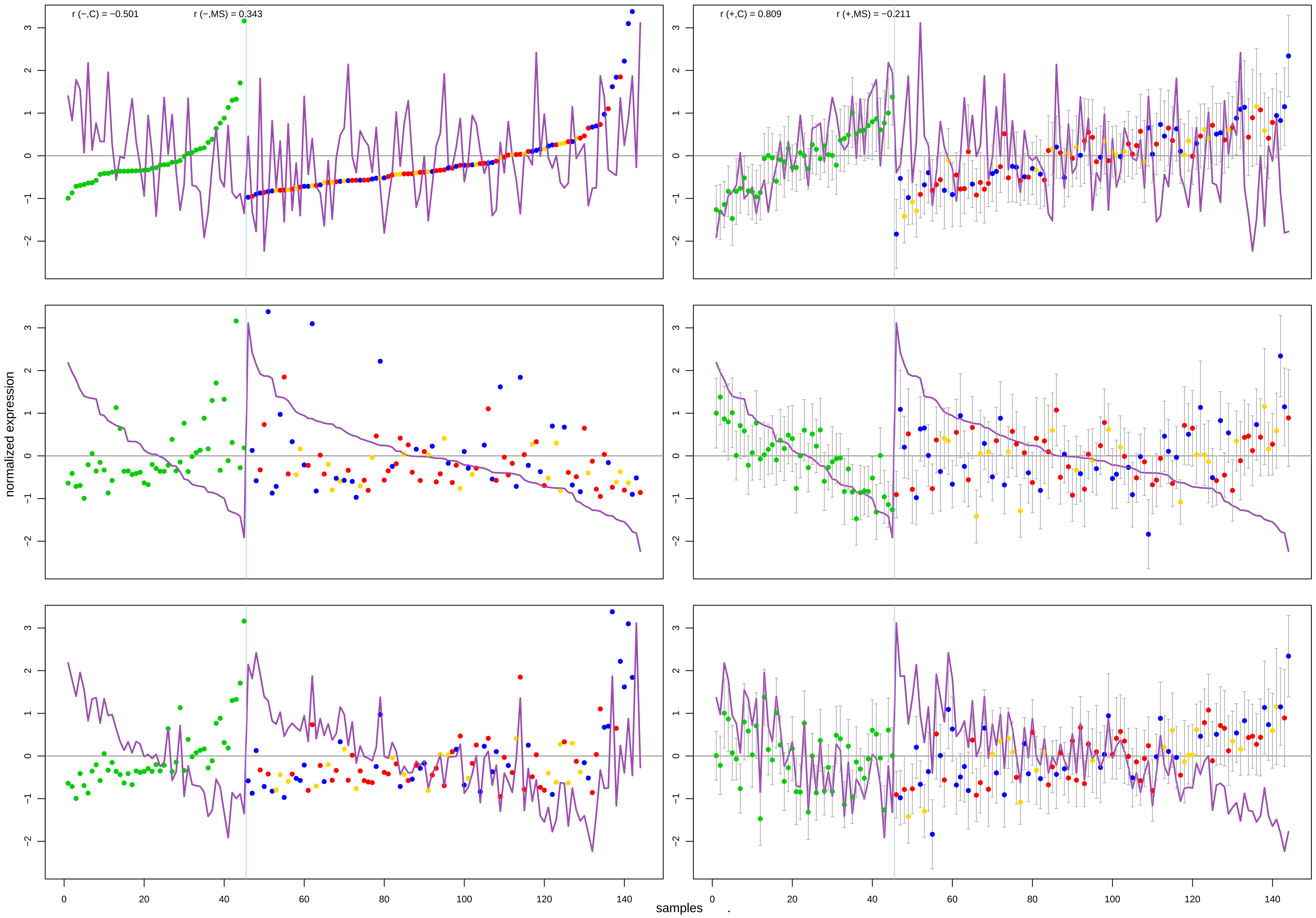

r (−,C) = −0.501
r (−,MS) = 0.343
r (+,C) = 0.809
r (+,MS) = −0.211
normalized expression
samples

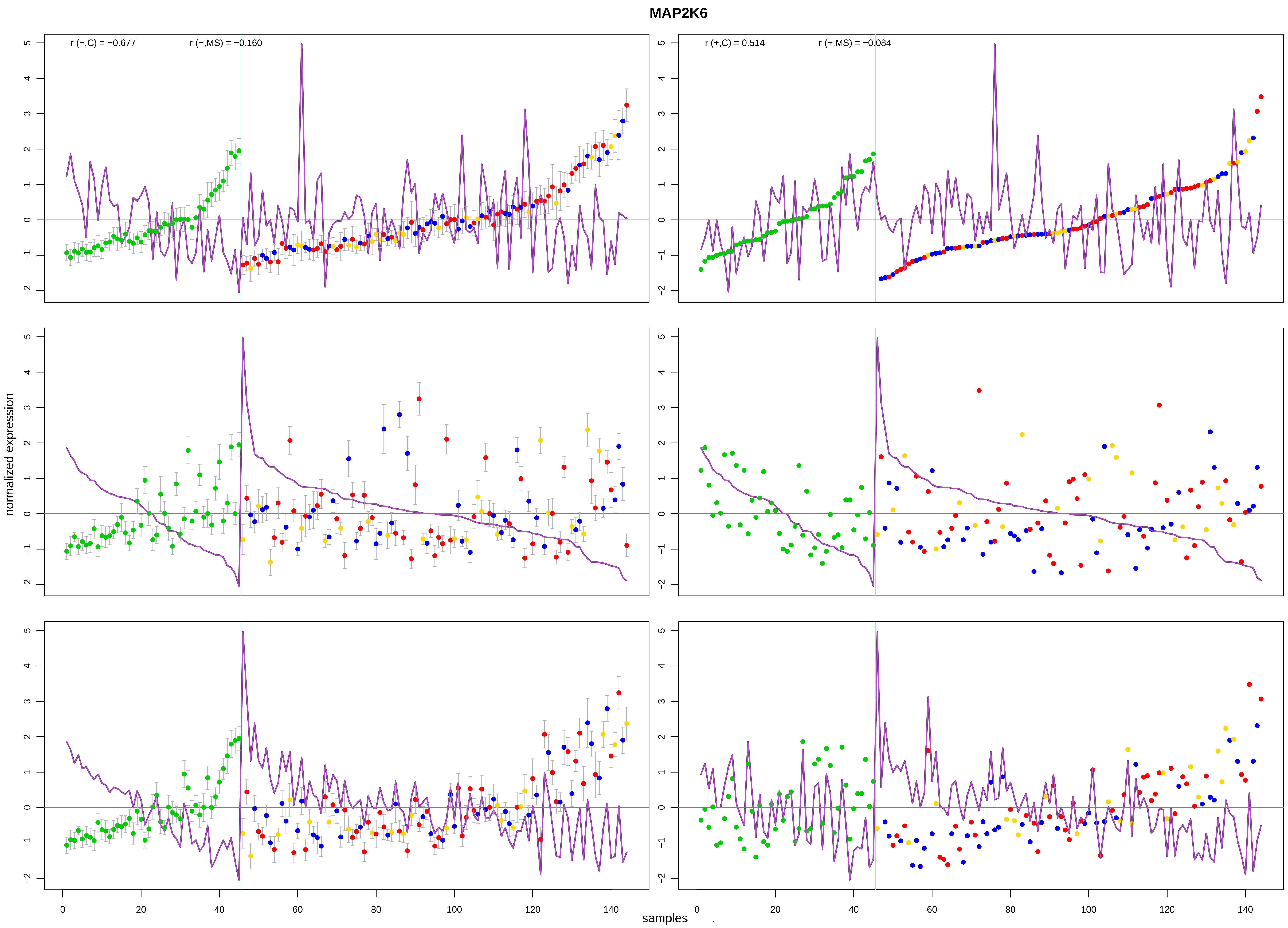

MAP2K6
r (−,C) = −0.677
r (−,MS) = −0.160
r (+,C) = 0.514
r (+,MS) = −0.084
normalized expression
samples

MAPK3

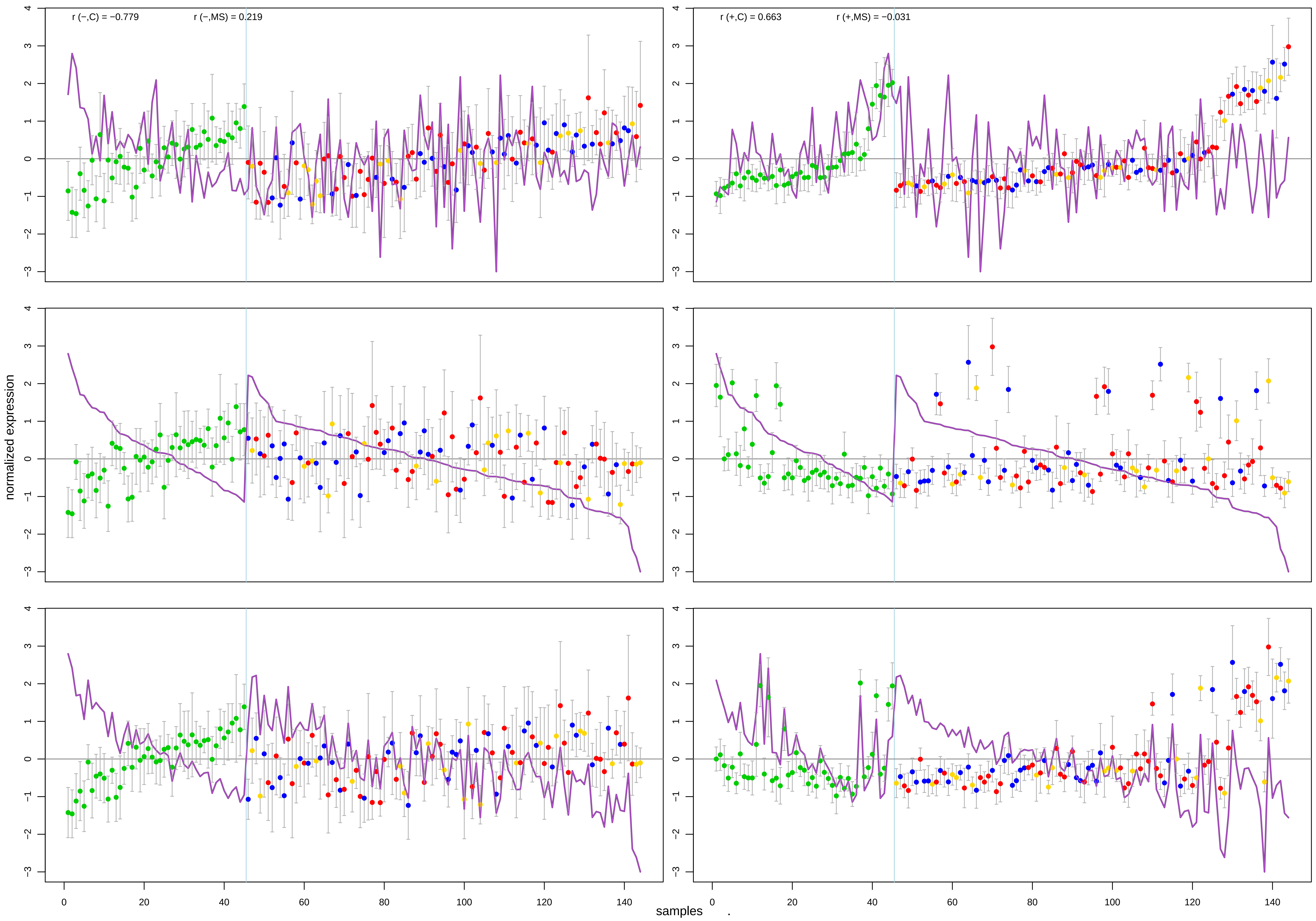

r (−,C) = −0.779
r (−,MS) = 0.219
r (+,C) = 0.663
r (+,MS) = −0.031
normalized expression
samples

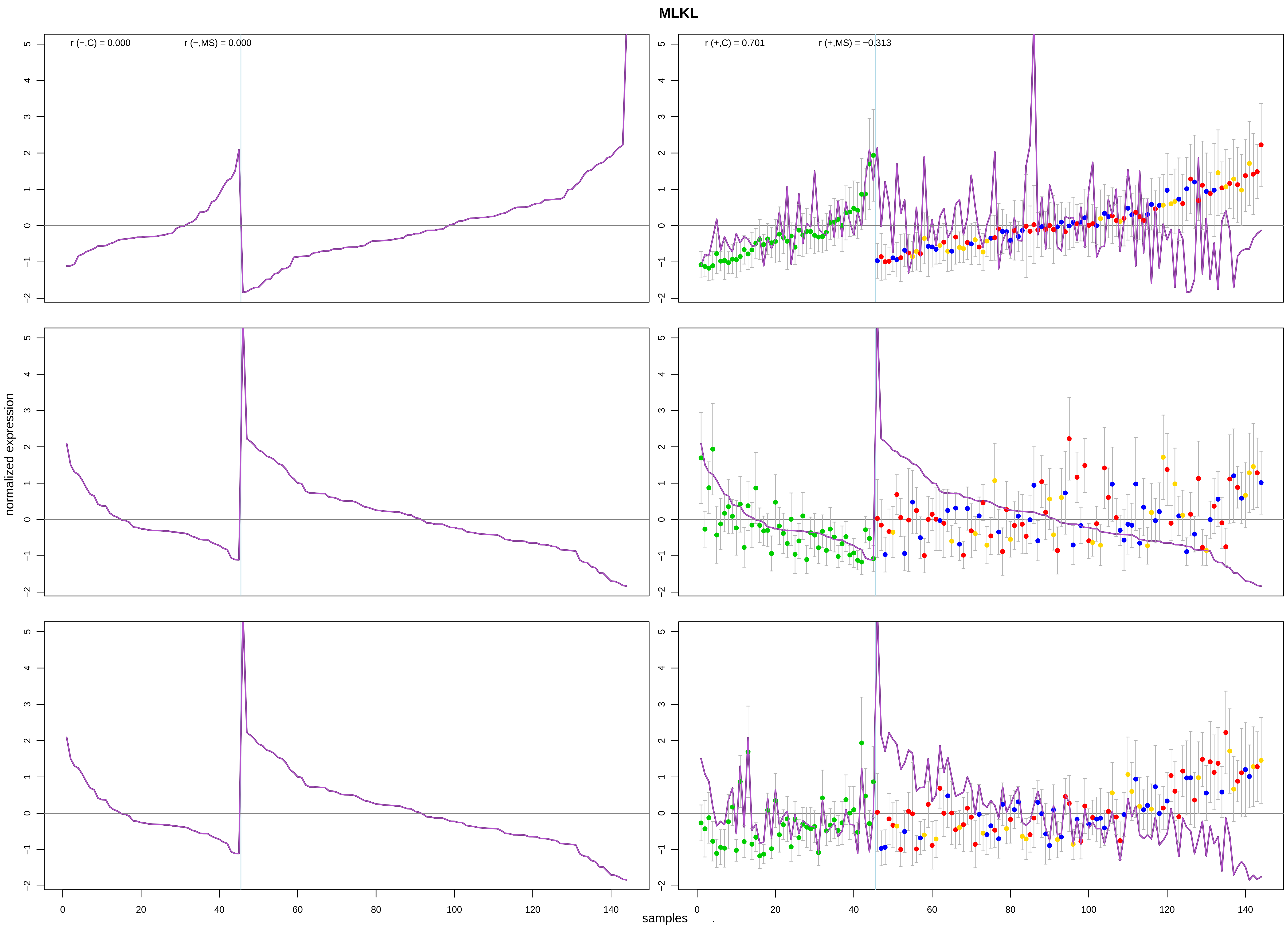
MLKL
r (−,C) = 0.000
r (−,MS) = 0.000
r (+,C) = 0.701
r (+,MS) = −0.313
normalized expression
samples

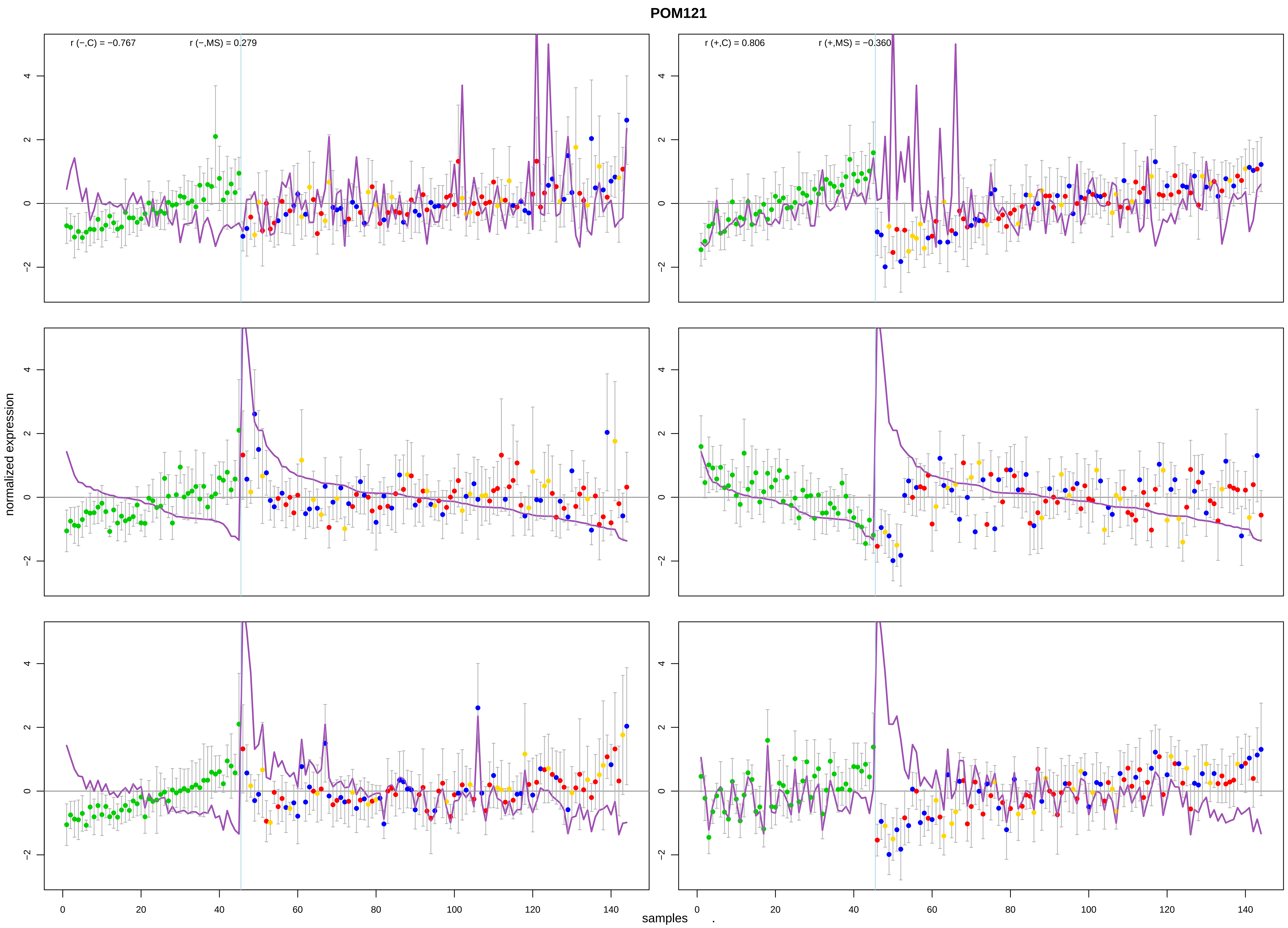
POM121
r (−,C) = −0.767
r (−,MS) = 0.279
r (+,C) = 0.806
r (+,MS) = −0.360
normalized expression
samples

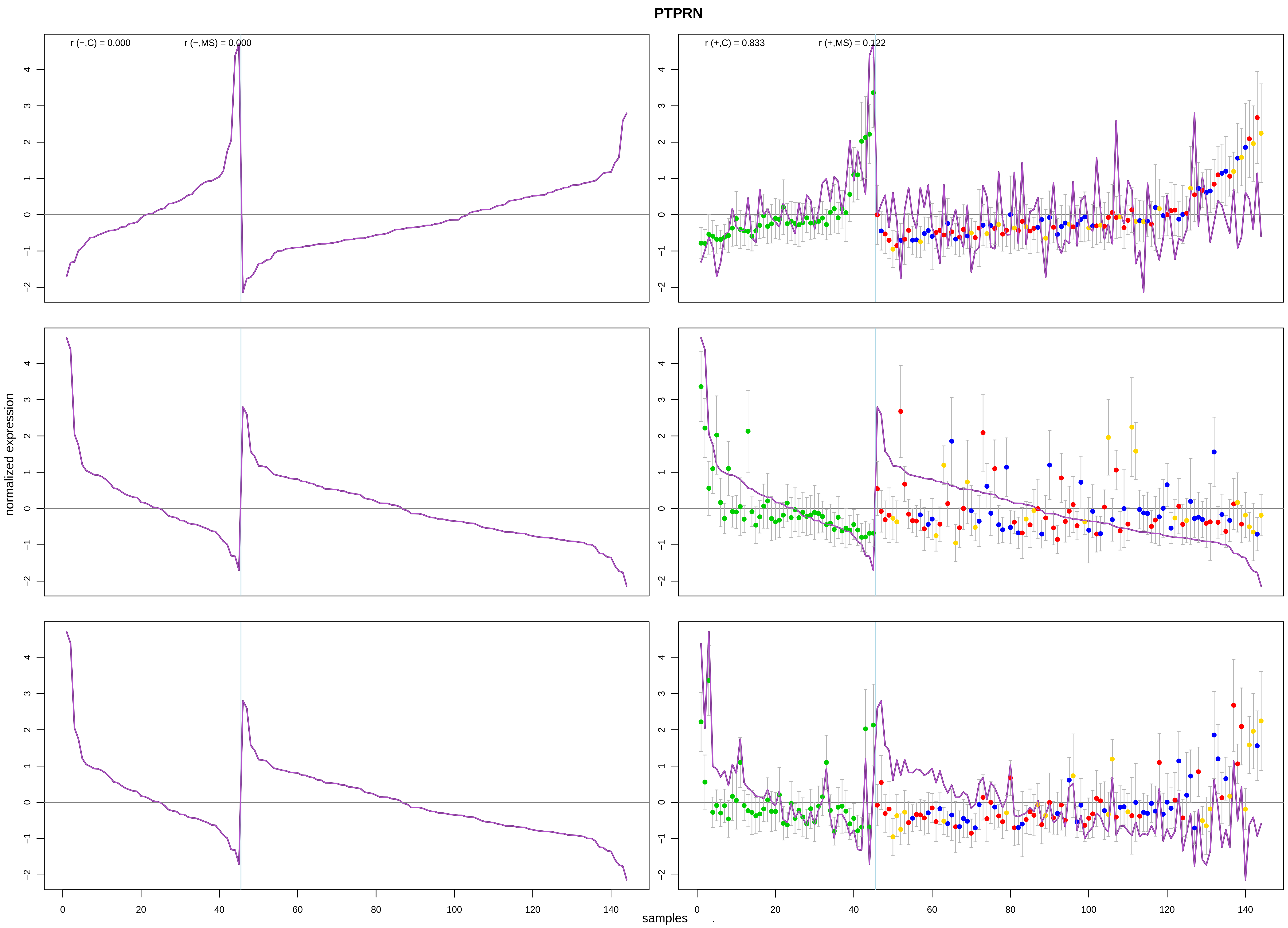

PTPRN
r (−,C) = 0.000
r (−,MS) = 0.000
r (+,C) = 0.833
r (+,MS) = 0.122
normalized expression
samples

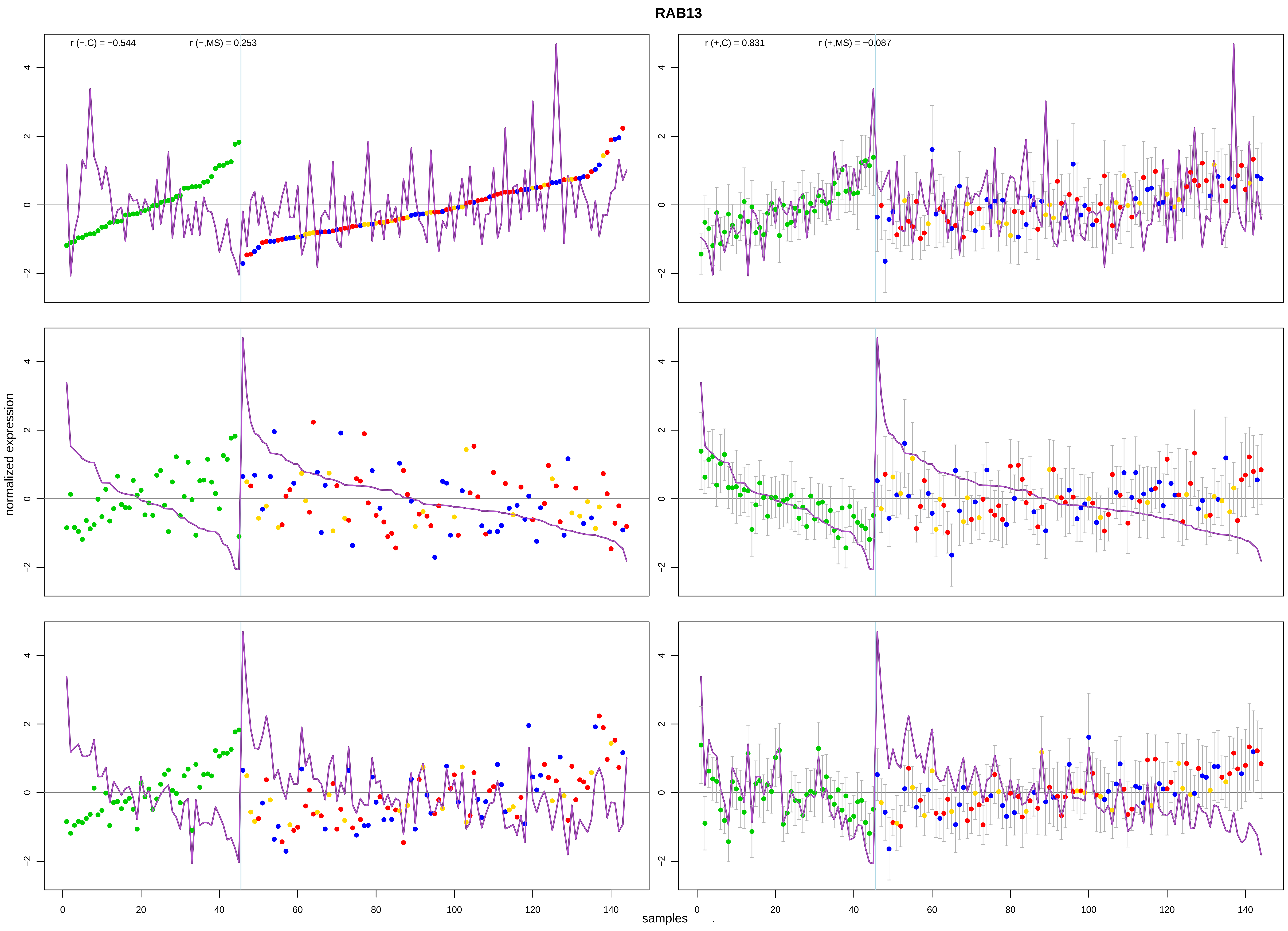

RAB13
r (−,C) = −0.544
r (−,MS) = 0.253
r (+,C) = 0.831
r (+,MS) = −0.087
normalized expression
samples

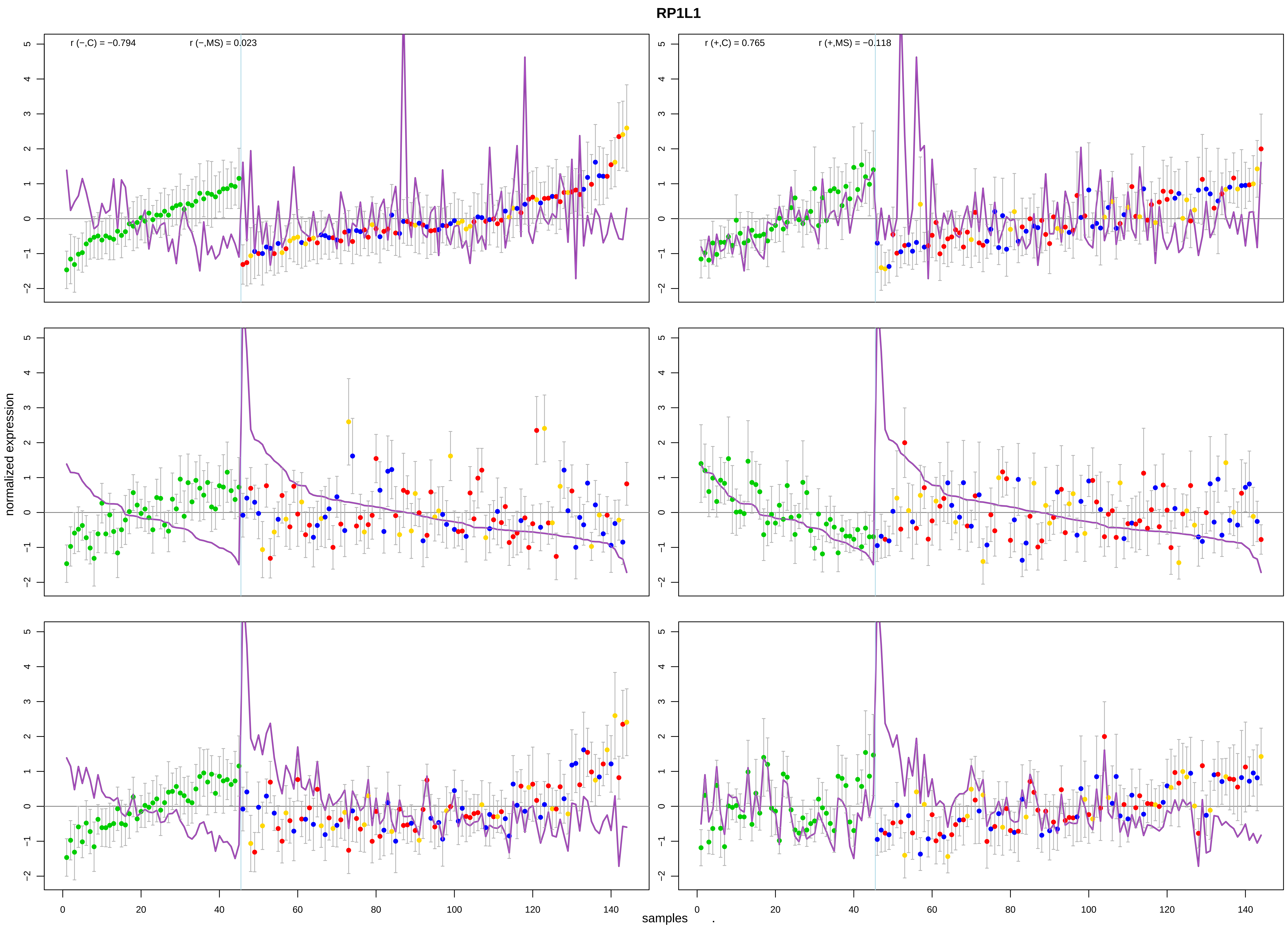

RP1L1
r (−,C) = −0.794
r (−,MS) = 0.023
r (+,C) = 0.765
r (+,MS) = −0.118
normalized expression
samples

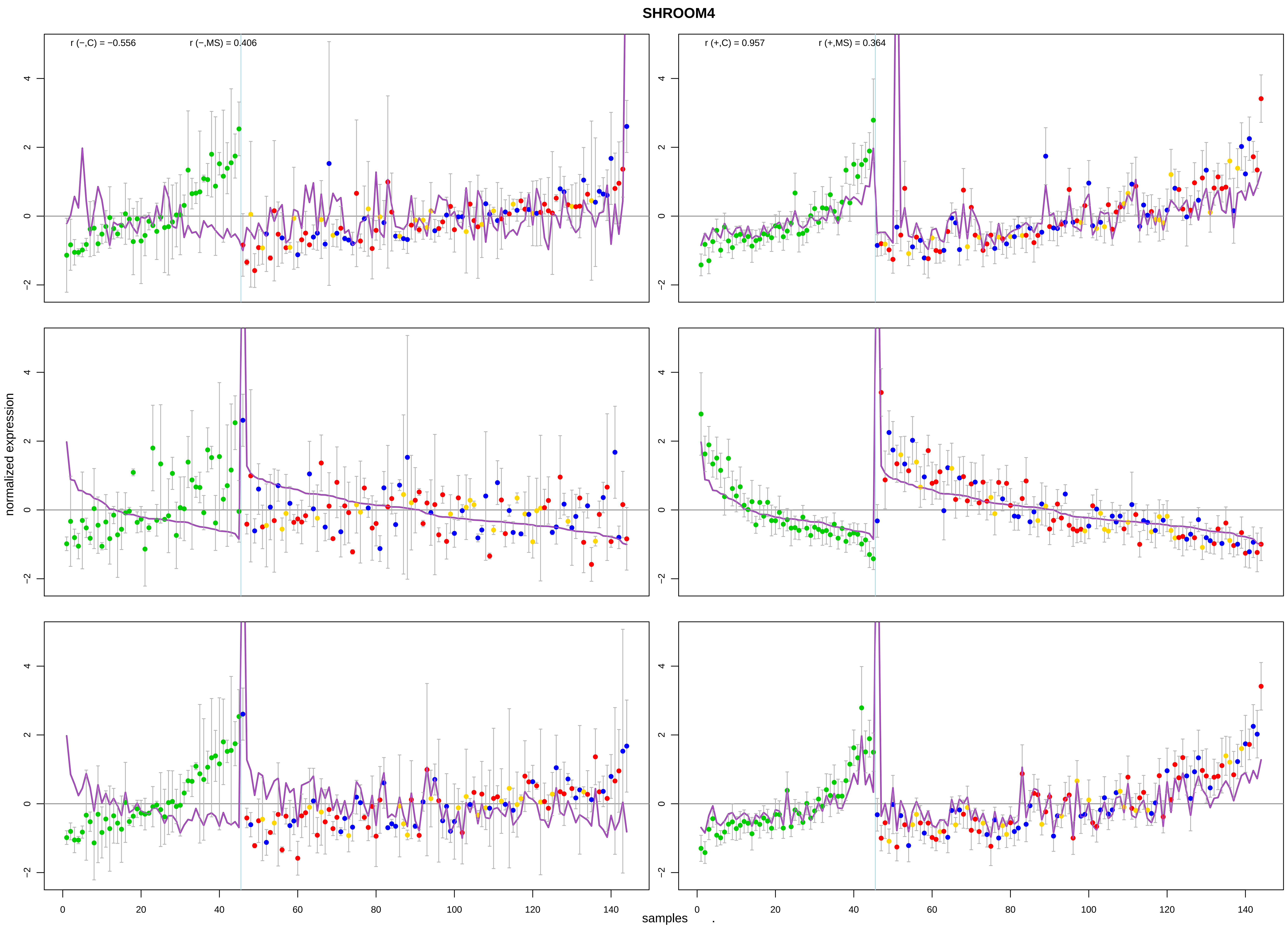

SHROOM4
r (−,C) = −0.556
r (−,MS) = 0.406
r (+,C) = 0.957
r (+,MS) = 0.364
normalized expression
samples

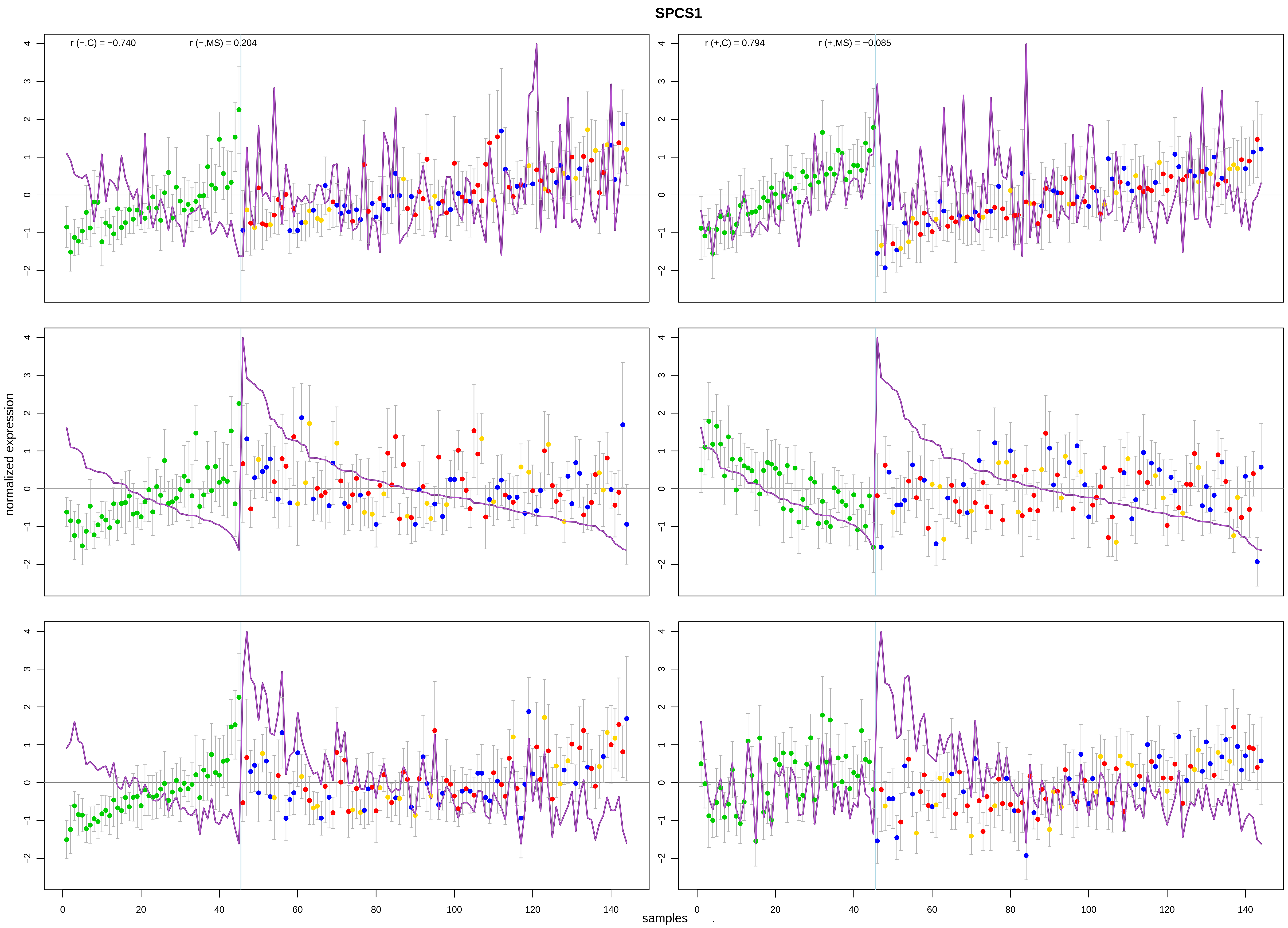

SPCS1
r (−,C) = −0.740
r (−,MS) = 0.204
r (+,C) = 0.794
r (+,MS) = −0.085
normalized expression
samples

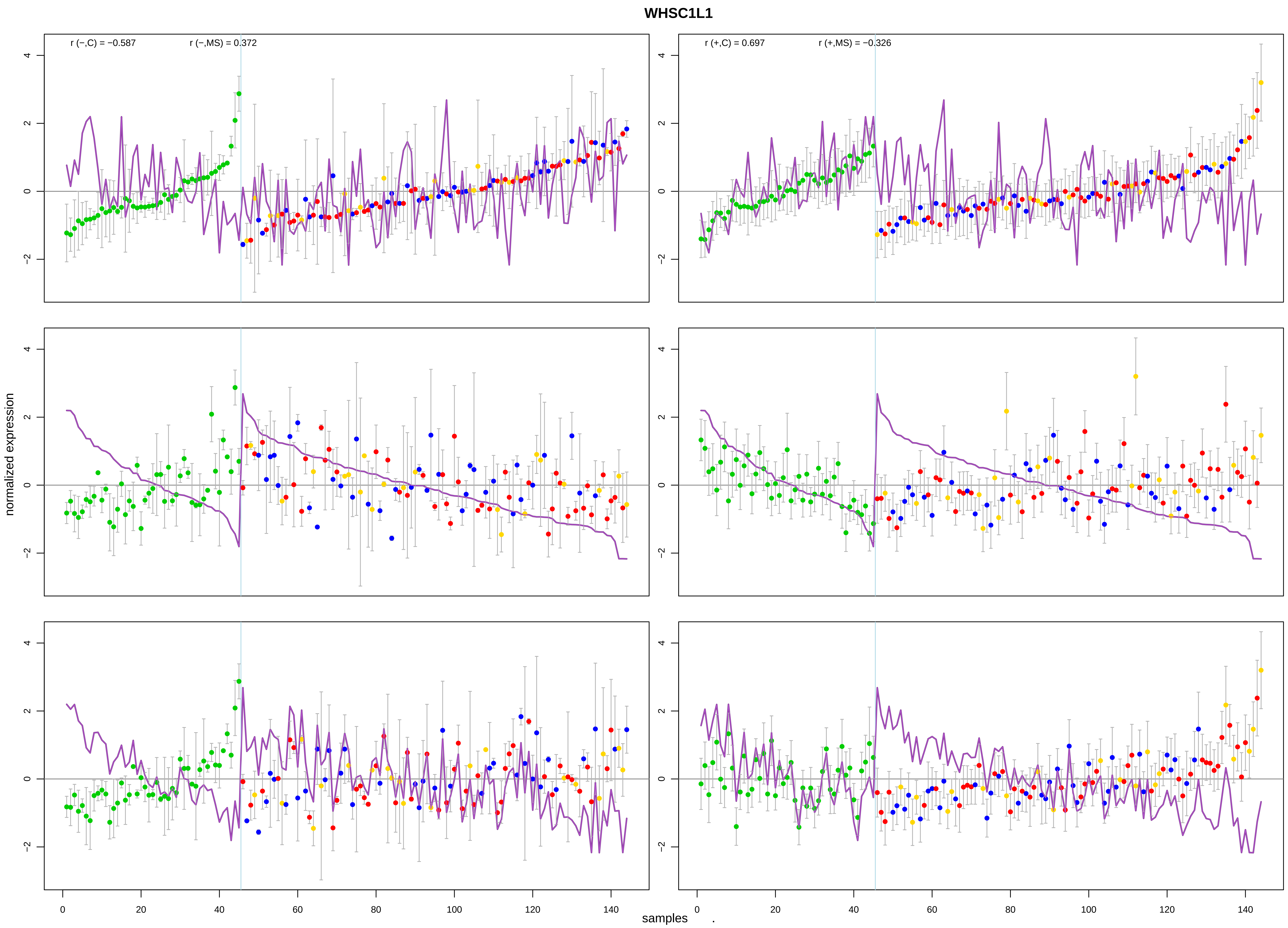
WHSC1L1
r (−,C) = −0.587
r (−,MS) = 0.372
r (+,C) = 0.697
r (+,MS) = −0.326
normalized expression
samples
